# Supplementary material for: Review of systematic reviews of non-pharmacological interventions to improve quality of life in cancer survivors
Source: BMJ Open. 2017 Nov 28;7(11):e015860. doi: 10.1136/bmjopen-2017-015860 (PMC5719270; doi:10.1136/bmjopen-2017-015860)
Supplement: Supplementary data [file bmjopen-2017-015860supp001.pdf]

Annex1: Full search strategy

Component 1: Population

#1. Neoplasms (mesh term) or cancer or cancers or cancerous or carcinoma* or neoplas* or tumor* or tumour* or malignan*

Component 2: Intervention

#2. Counseling (mesh term) or psychotherapy (mesh term) or “cognitive therapy” (mesh term) or “self-help groups” (mesh term) or “mind body therapies” (mesh term) or “behavior therapy” (mesh term) or psychotherapy, group (mesh term) or meditation, (mesh term) or “mindfulness” (mesh term) behaviour therapies, cognitive (mesh term)

#3. (counsel*:ti,ab or psychoeducat*:ti,ab or educat*:ti,ab or coping*:ti,ab or psychological*:ti,ab or psychosocial*:ti,ab or psychotherap*:ti,ab or psychoanalytic*:ti,ab) AND (therap*:ti,ab or treatment*:ti,ab or outcome*:ti,ab or intervention*:ti,ab)
#4. (social: ti,ab or peer: ti,ab or group: ti,ab) AND (support: ti,ab)
#5. self:ti,ab AND help:ti,ab
#6. (cognitive:ti,ab or behav*:ti,ab) AND (treatment*:ti,ab or therap*:ti,ab)
#7. “CBT”:ti,ab
#8. (Family:ti,ab or couple:ti,ab) AND (therap*:ti,ab)
#9. meditation:ti,ab or mindfulness:ti,ab
#10. #2 or#3or#4or#5or#6or#7or#8+or#9

Component 3: Outcome

#11. “quality of life” (mesh term) or “well being”: ti,ab or “QoL” (all fields) or “quality of life”: ti,ab

FULL PICO:

#1 AND #10 AND #11

Filters: Humans, English language, Reviews, Age group
