# Supplementary material for: Review of systematic reviews of non-pharmacological interventions to improve quality of life in cancer survivors
Source: BMJ Open. 2017 Nov 28;7(11):e015860. doi: 10.1136/bmjopen-2017-015860 (PMC5719270; doi:10.1136/bmjopen-2017-015860)
Supplement: Supplementary data [file bmjopen-2017-015860supp002.pdf]

ONLINE SUPPLMENT: Excluded papers from the full paper search

**Author**	**Date**	**Reference**	**Reason for exclusion**
Kirshbaum et al.	2007	Kirshbaum MN. A review of the benefits of whole body exercise during and after treatment for breast cancer. *Journal of clinical nursing*. 2007.	This review was excluded as it was not a systematic review.
Badr et al.	2013	Badr H, Krebs P. A systematic review and meta-analysis of psychosocial interventions for couples coping with cancer*. Psycho-Oncology.* 2013.	This review was excluded as it was not cancer survivors, who have completed active treatment.
Jones et al.	2006	Jones LW, Demark-Wahnefried W. Diet, exercise, and complementary therapies after primary treatment for cancer. *The lancet oncology.* 2006.	This review was excluded as it was not systematic.
Burden et al.	2014	Burden S, Gibson DJ, Todd C, Gratton EK, Pilling M, Lal S. Dietary interventions for adult cancer survivors. *The Cochrane Library.* 2014.	This review was excluded as it was a protocol paper. We emailed the authors however, we were unable to find paper.
Keesing et al.	2015	Martin TA, Moran-Kelly RM, Roberts LM, Powe JG, Farrell SN, Singleton J. Effectiveness of individualized survivorship care plans on quality of life of adult female breast cancer survivors: a systematic review (provisional abstract*). JBI Database of systematic reviews and implementation reports.* 2015.	This paper was not a non-pharmacological psychosocial intervention.
Luckett et al.	2011	Luckett T, Britton B, Clover K, Rankin NM. Evidence for interventions to improve psychological outcomes in people with head and neck cancer: a systematic review of the literature. *Supportive care in cancer*. 2011.	This review was excluded as it was not cancer survivors, who have completed active treatment.
Friendenreich and Courneya	1996	Friedenreich CM, Courneya KS. Exercise as rehabilitation for cancer patients (structured abstract). *Clinical Journal of Sport Medicine*. 1996.	This review was excluded as it was a structured abstract only. We emailed the authors however, we were unable to find paper
Cheng et al.	2014	Cheng KK, Lim YT, Koh ZM, Tam WW. Home-based multidimensional survivorship programmes for breast cancer survivors. *The Cochrane Library.* 2014.	This review was excluded as it was a protocol paper. We emailed the authors however, we were unable to find paper.

de Boer et al.	2011	De Boer AG, Taskila T, Tamminga S, Frings-Dresen M, Feuerstein M, Verbeek J. Interventions to en-hance return-to-work for cancer patients. *Cochrane Database Syst Rev.* 2011.	This paper was excluded as QOL was not main outcome and it was not cancer survivors who had completed active treatment.
Solloway et al.	2016	Yan JH, Pan L, Zhang XM, Sun CX, Cui GH. Lack of efficacy of Tai Chi in improving quality of life in breast cancer survivors: a systematic review and meta-analysis (provisional abstract). *Asian Pacific journal of cancer prevention: APJCP*. 2014.	This review was excluded as it was a provisional abstract only. We emailed the authors however, we were unable to find paper
Ledesma et al.	2009	Ledesma D, Kumano H. Mindfulness-based stress reduction and cancer: a meta-analysis. *Psycho-Oncology.* 2009.	This review was excluded as it was not cancer survivors, who have completed active treatment.
Scott et al.	2013	Scott DA, Mills M, Black A, Cantwell M, Campbell A, Cardwell CR, Porter S, Donnelly M. Multidimensional rehabilitation programmes for adult cancer survivors. *The Cochrane Library.* 2013.	This paper was excluded as QOL was not main outcome
Khan et al.	2013	Khan F, Amatya B, Ng L, Drummond K, Olver J. Multidisciplinary rehabilitation after primary brain tumour treatment. *Cochrane Database syst rev.* 2013.	This review was excluded as it was not cancer survivors, who have completed active treatment.
Budhrani et al.	2014	Budhrani P. Optimal Timing of Mindfulness-Based Stress Reduction in Cancer: Research Synthesis and State of the Science. *The Journal of Alternative and Complementary Medicine.* 2014.	We were unable to access the full paper. Authors confirmed this was a presentation rather than a paper.
Oldervoll et al.	2004	Oldervoll LM, Kaasa S, Hjermstad MJ, Lund JÅ, Loge JH. Physical exercise results in the improved subjective well-being of a few or is effective rehabilitation for all cancer patients? (provisional abstract). *European Journal of Cancer.* 2004.	This review was excluded as it was not cancer survivors, who have completed active treatment. It included only two primary papers with cancer survivors, and not analysed separately.
Casellas-Grau et al.	2014	Casellas-Grau A, Font A, Vives J. Positive psychology interventions in breast cancer. A systematic review. *Psycho-Oncology*. 2014.	This review was excluded as it was not cancer survivors, who have completed active treatment.

Hulbert-Williams et al.	2015	Hulbert-Williams NJ, Storey L, Wilson KG. Psychological interventions for patients with cancer: psychological flexibility and the potential utility of Acceptance and Commitment Therapy. *European journal of cancer care.* 2015.	This review was excluded as it was not cancer survivors, who have completed active treatment.
Harder et al.	2012	Harder H, Parlour L, Jenkins V. Randomised controlled trials of yoga interventions for women with breast cancer: a systematic literature review. *Supportive care in cancer.* 2012.	This review was excluded as it was not cancer survivors, who have completed active treatment.
De Backer et al.	2009	De Backer IC, Schep G, Backx FJ, Vreugdenhil G, Kuipers H. Resistance training in cancer survivors: a systematic review (provisional abstract). *International journal of sports medicine.* 2009.	This paper was excluded as QOL was not main outcome.
Archer et al.	2015	Archer S, Buxton S, Sheffield D. The effect of creative psychological interventions on psychological outcomes for adult cancer patients: a systematic review of randomised controlled trials. *Psycho-Oncology.* 2015.	This review was excluded as it was not cancer survivors, who have completed active treatment.
Piet et al.	2012	Piet J, Würtzen H, Zachariae R. The effect of mindfulness-based therapy on symptoms of anxiety and depression in adult cancer patients and survivors: A systematic review and meta-analysis. *Journal of Consulting and Clinical Psychology.* 2012.	This paper was excluded as QOL was not main outcome.
Cramp et al.	2010	Cramp F, James A, Lambert J. The effects of resistance training on quality of life in cancer: a systematic literature review and meta-analysis. *Supportive care in cancer*. 2010.	This review was excluded as it was not cancer survivors, who have completed active treatment.
Stan et al.	2012	Stan DL, Collins NM, Olsen MM, Croghan I, Pruthi S. The evolution of mindfulness-based physical interventions in breast cancer survivors. *Evidence-Based Complementary and Alternative Medicine*. 2012.	This paper was excluded as it is not a systematic review
Bouma et al.	2015	Bouma G, Admiraal JM, de Vries EG, Schröder CP, Walenkamp AM, Reyners AK. Internet-based support programs to alleviate psychosocial and physical	This review was excluded as it was not cancer survivors, who have completed active treatment.

		symptoms in cancer patients: a literature analysis. *Critical reviews in oncology/hematology.* 2015.	
Bourke et al.	2016	Bourke L, Smith D, Steed L, Hooper R, Carter A, Catto J, Albertsen PC, Tombal B, Payne HA, Rosario DJ. Exercise for men with prostate cancer: a systematic review and meta-analysis. *European urology.* 2016.	This review was excluded as it was not cancer survivors, who have completed active treatment.
Post et al.	2016	Post KE, Flanagan J. Web based survivorship interventions for women with breast cancer: An integrative review. European *Journal of Oncology Nursing*. 2016.	This paper was excluded as QOL was not main outcome.
