# Supplementary material for: Review of systematic reviews of non-pharmacological interventions to improve quality of life in cancer survivors
Source: BMJ Open. 2017 Nov 28;7(11):e015860. doi: 10.1136/bmjopen-2017-015860 (PMC5719270; doi:10.1136/bmjopen-2017-015860)
Supplement: Author's manuscript [file bmjopen-2017-015860.draft_revisions.pdf]

BMJ Open is committed to open peer review. As part of this commitment we make the peer review history of every article we publish publicly available.

When an article is published we post the peer reviewers' comments and the authors' responses online. We also post the versions of the paper that were used during peer review. These are the versions that the peer review comments apply to.

The versions of the paper that follow are the versions that were submitted during the peer review process. They are not the versions of record or the final published versions. They should not be cited or distributed as the published version of this manuscript.

BMJ Open is an open access journal and the full, final, typeset and author-corrected version of record of the manuscript is available on our site with no access controls, subscription charges or pay-per-view fees (http://bmjopen.bmj.com).

If you have any questions on BMJ Open's open peer review process please email editorial.bmjopen@bmj.com

BMJ Open

A Rapid Review of Systematic Reviews of non-pharmacological interventions to improve quality of life and well-being in cancer survivors.

Journal:	*BMJ Open*
Manuscript ID	bmjopen-2017-015860
Article Type:	Research
Date Submitted by the Author:	11-Jan-2017
Complete List of Authors:	Duncan, Morvwen; Barts and The London School of Medicine & Dentistry Queen Mary University of London, Centre for Psychiatry at the Wolfson Institute of Preventive Medicine<br>Deane, Jennifer; Barts and The London School of Medicine & Dentistry Queen Mary University of London, Centre for Psychiatry at the Wolfson Institute of Preventive Medicine<br>Roylance, Rebecca ; University College London<br>Jones, Louise; University College Medical School, Marie Curie Palliative Care Unit, UCL Mental Health Sciences Unit<br>Bourke, Liam; University of Sheffield Medical School<br>Morgan , Adrienne ; Queen Mary University of London - Charterhouse Square Campus, Barts Cancer Institute<br>Chalder, Trudie; King's College London, Psychological Medicine<br>Thaha, Mohamed; Queen Mary's University of London, National Centre for Bowel Research & Surgical Innovation<br>Taylor, Stephanie; Barts and The London School of Medicine & Dentistry Queen Mary University of London, Centre for Psychiatry at the Wolfson Institute of Preventive Medicine<br>Korszun, Ania; Barts and The London School of Medicine and Dentistry, Psychiatry<br>White, Peter; Barts and the London School of Medicine, Queen Mary University of London, Centre for Psychiatry<br>bhui, kamaldeep; Barts and The London School of Medicine & Dentistry Queen Mary University of London, Centre for Psychiatry at the Wolfson Institute of Preventive Medicine
**Primary Subject Heading**:	Oncology
Secondary Subject Heading:	Public health, Patient-centred medicine
Keywords:	ONCOLOGY, PUBLIC HEALTH, REHABILITATION MEDICINE

SCHOLARONE™
Manuscripts

A Rapid Review of Systematic Reviews of non-pharmacological interventions to improve quality of life and well-being in cancer survivors.

Morvwen Duncan, Jennifer Deane, Rebecca Roylance, Louise Jones, Liam Bourke, Adrienne Morgan, Trudie Chalder, Mo Thaha, Stephanie Taylor, Ania Korszun, Peter White, *Kamaldeep Bhui on behalf of SURECAN Investigators.

Morvwen Duncan

Research Assistant

Academic Psychological Medicine, Wolfson Institute of Preventive Medicine, Barts and the London School of Medicine and Dentistry, Queen Mary University of London, EC1A 7BE

Jennifer Deane

Research Assistant

Academic Psychological Medicine, Wolfson Institute of Preventive Medicine, Barts and the London School of Medicine and Dentistry, Queen Mary University of London, EC1A 7BE

Wolfson Institute of Preventive Medicine, Barts and the London School of Medicine and Dentistry, Queen Mary University of London, EC1A 7BE

Rebecca Roylance

Consultant Medical Oncologist and Honorary Senior Lecturer

University College Hospitals NHS Foundation Trust and UCLH BRC

UCLH & UCL.

Louise Jones

Clinical Senior Lecturer

Marie Curie Palliative Research Dept, Division of Psychiatry, UCL.

Liam Bourke

Reader in Clinical Science

Sheffield Hallam University.

Adrienne Morgan

Honorary Senior Lecturer
Centre for Tumour Biology
Barts Cancer Institute - Queen Mary University of London

Trudie Chalder

Professor of Cognitive Behavioural Psychotherapy

Department of Psychological Medicine, King's College London, Denmark Hill, King's College, London, SE59RJ

Mohamed A Thaha

Senior Lecturer & Consultant in Colorectal Surgery

For peer review only

Blizard Institute, National Centre for Bowel Research & Surgical Innovation
Barts and the London School of Medicine & Dentistry, Queen Mary University of London.

Stephanie Taylor Professor in Public Health and Primary Care

Centre for Primary Care and Public Health, Blizard Institute, Barts and The London School of Medicine and Dentistry, London, E1 2AB

Ania Korszun

Professor of Education and Psychiatry

Wolfson Institute of Preventive Medicine, Barts and the London School of Medicine and Dentistry, Queen Mary University of London, EC1M 6BQ

Peter White

Professor of Psychological Medicine

Wolfson Institute of Preventive Medicine, Barts and the London School of Medicine and Dentistry, Queen Mary University of London, EC1A 7BE

* Kamaldeep Bhui

Professor of Cultural Psychiatry & Epidemiology

Wolfson Institute of Preventive Medicine, Barts and the London School of Medicine and Dentistry, Queen Mary University of London, EC1A 7BE

& SURECAN Research Group

*Correspondence to: Kamaldeep Bhui, k.s.bhui@qmul.ac.uk, 020 7882 2012

Key Words: *Cancer, quality of life, interventions,*

Word Count:

Paper excluding abstract, tables and references: 3167

Abstract: 312

For peer review only

ABSTRACT

Objectives

Some two million people in the UK are living with and beyond cancer, with a third reporting a diminished quality of life. The study seeks to identify effective non-pharmacological interventions to improve the quality of life of cancer survivors.

Design A rapid review of published systematic reviews

Data Sources Databases searched included PubMed, Cochrane Central, EMBASE/MEDLINE, Web of Science and Psych INFO.

Study selection

Published systematic reviews of randomised trials of non-pharmacological interventions for people living with and beyond cancer were included; study participants were aged 18 or older and received their cancer diagnosis in adulthood. Interventions located in any healthcare setting were included. Reviews of alternative therapies or not reported in English were excluded. Two researchers independently assessed titles, abstracts and the full text of papers and extracted the data.

Outcomes

The primary outcome of interest was any measure of global (overall) quality of life.

Analytic methods

For peer review only

Study quality was assessed with the R-AMSTAR (Revised Assessment of Multiple Systematic Reviews) and was considered in a thematic narrative synthesis, comparing effectiveness of non-pharmacological interventions and the components.

Results

Of 12, 947 unique titles, 16 publications (five with high methodological quality) met study inclusion criteria. There was little overlap in the primary papers considered across these reviews. Nine reviews covered mixed tumour groups and seven focused on breast cancer. Face-to-face interventions were often combined with online, telephone and paper-based reading materials. The emergent classification of interventions included physical, psychological or behavioural, multidimensional rehabilitation and online approaches. Yoga, specifically, and physical exercise, more generally, were associated with consistent benefits for quality of life, as was cognitive behavioural therapy (CBT).

Conclusions

Exercise-based interventions were shown to be effective in the short (less than 3-8 months) and long term, irrespective of the types of meta-analyses undertaken. CBT also showed benefits, especially in the short term. The evidence for multidisciplinary, online, and educational interventions was equivocal.

For peer review only

ARTICLE SUMMARY

Strengths and limitations of this study

- **This systematic evidence synthesis provides information to carers and patients and professionals about the effective elements of non-pharmacological interventions in cancer survivors**
- **Physical activity and cognitive behaviour therapies show benefits for quality of life outcomes**
- **Longer terms studies are needed and studies of greater methodological quality adopting similar reporting standards**
- **Definitions of survivor varied and more studies are needed for different types of cancer, and for those with poor quality of life**
- **More studies are needed investigating educational, online and multidisciplinary team based interventions**
- **This was a review of reviews, and we did not review individual studies focussed on a specific cancers or staging**

For peer review only

INTRODUCTION

Advances in public awareness, early detection and improved treatments mean that more people are now living with and beyond cancer. For example, Cancer Research UK reports that 50% of people diagnosed with cancer in England and Wales survive 10 years or more, and survival rates have doubled over the last 40 years.[1] This group of survivors includes people at various stages of active treatment, and those in remission, who are gradually restoring their social and occupational roles.

A significant proportion of cancer survivors experience poor quality of life.[2] The main causes of poor quality of life include depression, anxiety, distress, fear of recurrence, lower levels of social support; impacts on relationships, family, and social function; psychological and social needs, and problems coping.[2 3] The process of diagnosis and treatment is traumatic and disruptive. It is not unusual for cancer patients to experience distress. Common experiences in those living with and beyond cancer include reduced physical ability, fatigue, changes in sexual activity and developing other medical conditions that affect function for many years.[2 3] If a person is suffering from fatigue, depression or anxiety they are understandably less motivated to visit friends or engage in social activities; the strain on marital relationships may lead to a loss of support; 25% of people who experience difficulties have broken up with their partner as a result of cancer.[3 4] Thus, the effects of cancer extends beyond the diagnostic and active treatment phases. This review aims to gather the evidence for practitioners, patients and their carers about effective non-pharmacological interventions to improve quality of life in cancer survivors. We sought to summarise the effectiveness of interventions and to provide a component analysis of the content of interventions.

METHODS

This review of reviews examined existing systematic reviews of non-pharmacological interventions that include information on quality of life of those living with and beyond cancer.

For peer review only

Inclusion and exclusion criteria

The study included any systematic reviews that explicitly reported randomised controlled trials. Inclusion criteria were organised in accord with the PICO reporting structure (see Table 1). The population of interest was people living with and beyond cancer, who were aged 18 years or more, and who had received their cancer diagnosis as adults.

We defined non-pharmacological interventions as those that did not involve any drug or medicine, but they could include educational, behavioural, psychosocial approaches or physical activity; we excluded complementary and alternative therapies as defined by the NHS Choices resource [5] However, we included physical activity and psychological approaches that were part of yoga based interventions. Comparators were not specified for the purpose of the inclusion criteria of the review of reviews, but comparators reported in the original reviews were considered in the analysis. The primary outcome was quality of life (QoL) defined by physical, psychological and social functioning. We reported only on studies that used an established and validated measure of global or overall QoL; some of these were cancer specific. In the literature, the terms 'Quality of Life' and 'Health Related QoL' are used interchangeably, therefore both are included under the term QoL in this review. The study settings included any healthcare venue, such as hospital inpatient or outpatient services, community services and included remote e-technology based interventions.

For peer review only

Table 1: Application of the PICO search strategy

**Population**	Participants living beyond cancer, who have completed active treatment with curative intent; aged 18 or more who received their cancer diagnosis in adulthood.
**Intervention**	Non-pharmacological interventions. Psychological, Social and Physical Activity, excluding complementary and alternative therapies or medicines. Including yoga interventions with meditation, activity or mindfulness.
**Outcomes**	Quality of life.
**Setting**	Any healthcare setting: hospital (in-patient or outpatient, community or remote (e.g. using e-technology).
**Study Design**	Systematic reviews that had explicitly searched for RCTs. To be classified as a systematic review the following criteria were met:<br><br>- a systematic search strategy<br><br>- a screening procedure to identity relevant studies<br><br>- systematic data extraction and analysis procedures for RCTs

For peer review only

Data sources

We searched the databases PubMed, Cochrane Central, EMBASE/MEDLINE, Web of Science, The Cumulative Index to Nursing and Allied Health Literature (CINAHL) and Psych INFO from inception to June 2015. The final search strategy is shown in Annex 1. We consulted experts in the field to assess completeness of the list of identified reviews, and where necessary, contacted authors to secure the full text versions.

Study selection

Two authors (MD, JD) independently screened all titles and abstracts of studies identified by the search strategy against inclusion and exclusion criteria, and, where necessary, the full text was read. Discrepancies around inclusion were resolved by discussion or in consultation with a third author

when required (KB). We searched the reference lists of all included reviews to identify any further relevant reviews. The research team was not blinded to authors. Citations were downloaded and managed in an Endnote library.

Data extraction

Two authors (MD, JD) independently extracted data from each of the eligible reviews into a purpose built, pre-designed, structured template. The data extraction forms were then summarised in a table (see online supplementary table) and reviewed independently by a third reviewer (KB) Extracted data included the following information:

- Publication details: author, year, title, journal, country, format of publication.
- Study characteristics: number of primary studies, total number of participants, range of publication dates, gender, age range of participants and socioeconomic data, primary cancer site, length of time since final cancer treatment and type of treatment..
- Intervention design and evaluation: setting, description of the intervention and its components: physical components, psychosocial components, educational components; duration of intervention, follow up, number of treatment contacts, type of practitioner providing treatment, mode of delivery of intervention, and any outcomes.
- Documents: Availability of treatment manuals.
- Results: Main outcome measures, secondary outcome measures, narrative findings, adherence levels, patient satisfaction, effect sizes against intervention components.

For peer review only

Assessment of methodological quality of included reviews

We used the R-AMSTAR (Revised Assessment of Multiple Systematic Reviews)[6] tool to assess the methodological quality (see Table 2). This provides a raw score of 0 to 44. As there is no given threshold to a high or low quality on the R-AMSTAR, we tested the sensitivity of using 50th centile score as the threshold (22), resulting in 15 classified as high quality; using a threshold of 70th centile score (31) resulted in 6 papers classed as high quality. Reviews were classified as 'high quality' if they scored as 33 or above (75th centile) as it better differentiated the highest quality studies from the rest.

Data analysis and narrative synthesis

The intervention components were listed, followed by a narrative synthesis[7] that including understanding components of the interventions, exploring patterns of findings across studies and within primary reviews, and giving greater weight to studies of higher quality in the interpretation of the findings. Ultimately, the purpose is to put into text format the key findings from the most robust evidence available, to guide treatment and future research recommendations. The synthesis sets out reported effect sizes across studies, means and SD. Meta-analysis was not undertaken, due to heterogeneity of methods, outcomes, reporting of effect sizes (9 reviews did not provide effect sizes). The publications were segmented into those reporting meta-analyses to which the greatest weighting was given in the synthesis and conclusions; some reviews did not undertake or report meta-analyses and reported each study, trends and the range of effect sizes; a third group reported no effect sizes but provided narrative statements.

Patient and Public Involvement

Patients and carers (and respective organisations) were involved in the design and development of the programme development grant application (from which this review article paper is one output).

For peer review only

Patients and carers attended all the steering group meetings and were an integral part of the research team, commenting on and critiquing the inclusion and exclusion criteria, outcome selection, and the acceptability and likely value of interventions. As part of the steering group, they received and commented on study progression, and emergent findings, and reports. They are integral to the dissemination plans including sharing the publication, but also helping craft lay summaries of the overall research project and key findings.

RESULTS

Study selection

Electronic database searches yielded 12,947 unique reviews. From this 264 were included from the title search, followed by 39 from the abstract search. After scrutinising the full texts, 16 previous reviews entered this review (See Figure 1). The quality scores are shown in Table 2.

For peer review only

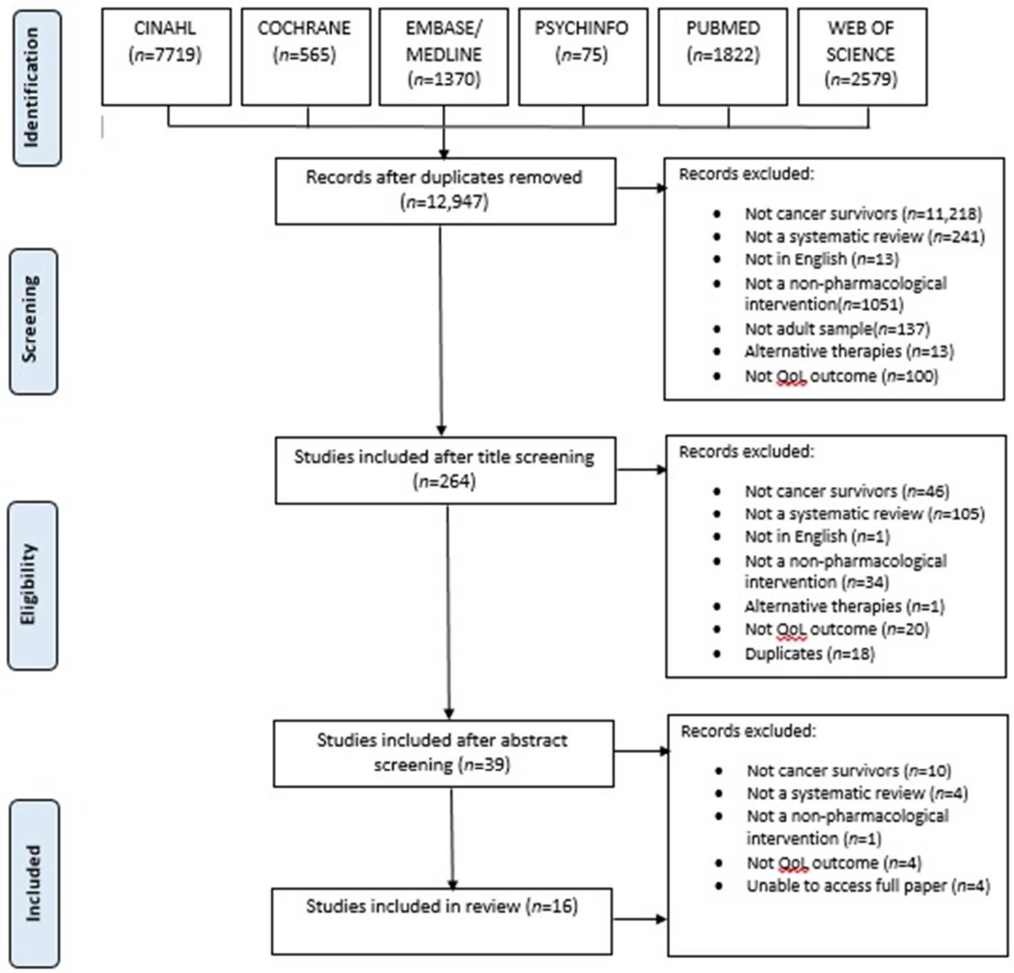

Figure 1: PRISMA flow diagram of study selection

Table 2: Quality rating using R-AMSTAR criteria

R-AMSTAR Criteria	Cramer et al, 2012[8]	Fong et al, 2012[9]	Buffart et al, 2012[10]	Khan et al, 2015[11]	Mishra et al. 2012[12]	Galveo et al. 2005[13]	Culos-Reed et al, 2012[14]	Fors et al, 2011[15]	McAlpine et al, 2015[16]	Spence et al, 2009[17]	Osborn et al, 2006[18]	Spark et al, 2013[19]	Zeng et al, 2014[20]	Mewes et al, 2012[21]	Duijts et al. 2011[22]	Ferrer et al, 2011[23]
Was an ''a priori'' design provided?	3	3	2	4	3	1	3	4	3	3	3	3	3	2	2	3
Was there duplicate study selection and data extraction?	2	3	4	4	4	1	1	4	2	1	4	3	4	4	2	3
Was a comprehensive literature search performed?	4	4	4	4	4	2	3	2	3	2	3	3	3	4	4	4

Was the status of publication i.e. grey literature) used as an inclusion criterion?	2	1	2	4	3	1	1	1	2	1	1	2	1	1	2	2
Was a list of studies included and excluded) provided?	3	2	2	4	4	1	1	1	2	2	1	2	1	3	1	2
Were the characteristics of the included studies provided?	4	4	4	4	4	4	4	3	3	4	4	4	3	4	3	3
Was the scientific quality of the included studies assessed and documented?	3	3	4	4	4	1	3	2	2	3	1	3	1	3	2	2
Was the scientific quality of the included studies used appropriately in formulating conclusions?	3	3	3	4	4	1	3	2	1	3	1	2	2	4	3	3
Were the methods used to combine the findings of the studies appropriate?	4	4	4	3	4	1	1	1	1	1	4	1	4	1	4	3
Was the likelihood of publication bias assessed?	2	3	2	2	4	1	1	1	1	1	3	1	2	2	3	3
Was the conflict of interest stated?	3	3	3	2	4	2	1	1	3	3	1	3	3	1	3	3
**Total score/44**	**33**	**33**	**34**	**39**	**42**	**16**	**22**	**22**	**22**	**24**	**26**	**27**	**27**	**29**	**29**	**31**
**Quality Rating Low = <33; High = 33+)**	**H**	**H**	**H**	**H**	**H**	**L**	**L**	**L**	**L**	**L**	**L**	**L**	**L**	**L**	**L**	**L**

Study characteristics

The types of intervention, settings, cancer type, measures of quality of life, and the key narrative findings are reported in Table 3.

Participants

The total number of patients included in the reviews ranged from 262[11] to 7164.[22] Nine reviews covered mixed tumour groups [9 12-14 16-18 21 23] and 7 specifically focused on breast cancer. [8 10 11 15 19 20 22]

For peer review only

Intervention Type and Components

Face-to-face delivery of interventions was often combined with online delivery (two reviews); [15 22] others included telephone communication (5 reviews) [8 10 19 20 22] and printed information (two studies). [10 19] Three reviews included interventions that provided supplementary compact discs, manuals or video tools. [8 10 15] Two reviews were from inpatient rehabilitation. [11 21] None of the reviews reported the use of structured manuals, and interventions were often not fully described or broken down into different components, nor was there attention to a mechanism or theory of change.

Seven of the reviews focused on physical interventions, [9 12 13 17 19 20 23] with three high quality [8 10 12] and three lower quality reviews including yoga; [14 20 22] three reviews were of psychosocial or behavioural interventions; [15 18 22] and one review focused on online interventions including connecting patients and online education [16] (see Tables 3 & 4). One review compared multi-dimensional versus mono-dimensional interventions [21] and one tested multidisciplinary rehabilitation models. [11] The duration and frequency of the interventions varied greatly from a single 20 minute session[18] to 60, weekly sessions.[9]

The most common component of physical interventions was aerobic exercise, [9 12 13 17 20 22 23] and resistance/strength training. [9 12 13 17 20 22] Psychological education [11 15 18 21 22] and cognitive behaviour therapy [15 18 21 22] were the most utilised psychological and educational intervention. Peer support was often used as a psychological and a behavioural intervention. [11 16 22] Components of the interventions were thematically organised into two groups (see Table 4 for a more detailed itemisation): biological or physical actions (19 types of activity or diet change) and psychological, behavioural or educational (24 types of intervention about mind and body: including cognitive behaviour therapy, psychosexual therapy, coping, emotional support, relaxation, psychotherapy and psychosocial therapy, and social

For peer review only - http://bmjopen.bmj.com/site/about/guidelines.xhtml

cognition interventions, social support, guided imagery, self-management, use of peer support, bibliotherapy, telephone and web-based interventions, return to work interventions).

For peer review only

Table 3: Characteristics of included reviews

Review	Aims of review	Number of primary studies	Participants	Definition of 'survivor'	Setting	Intervention, duration and frequencies	Outcome/measures<br><br>Follow ups??	Narrative findings
Buffart et al, 2012[10]	Systematic review of RCTs and meta-analysis of the effects of yoga in cancer patients and survivors.	16 papers on 13 RCTs	783 breast cancer patients during and after treatment.<br><br>Aged: 44-63 years.	During and post treatment.	Interventions were face to face, with supplementary CDs, manuals or telephone calls.	All included a yoga program led by experienced yoga instructors with physical poses (asanas), breathing techniques, (pranayama), and relaxation or meditation (savasana or dhanya).<br><br>Programme duration: 6 weeks to 6 months.	QoL measures included FACT, General HRQoL, EORTC QLQ-C30, FLIC	Yoga resulted in moderate increases in general quality of life at xx months follow up? (Or range of follow ups)
Cramer et al, 2012[8]	Systematic review & meta-analysis of the effects of yoga on health-related quality of life in breast cancer patients and survivors.	10	742 breast cancer patients during or after treatment.<br><br>Aged: 44-63 years.	Those who had completed active treatment before the onset of the study.	Face to face, with supplementary audio and video tools or telephone calls.	Yoga interventions including Iyengar yoga, Yoga of Awareness, (viniyoga), restorative yoga, yoga based on Patanjali's yoga tradition, yoga in daily life, and hatha yoga.<br><br>Duration: 1 week to 6 months.<br><br>Frequency varied from daily sessions to weekly.	QoL measures included; FACT-G, FACT-B, FACIT-Sp, FLIC, EORTC QLQ-C30,	There is moderate evidence for the short-term (meaning?) effect of yoga on global health-related quality of life.
Culos-Reed et al, 2012[14]	Determine the clinical significance of patient-reported outcomes from yoga interventions conducted with cancer survivors.	13	474 mixed cancer patients.<br><br>The majority were breast cancer patients during and after	After treatment defined as 3 months or more post treatment.		Yoga styles included hatha, integral, iyengar, tibetan, viniyoga, and vivekananda.<br><br>The duration: 6 to 26	QoL measures included EORTC, FACT-B, FACT-G, FACIT, NHP-Total,.	Yoga had a significantly positive impact on quality of life. When?

For peer review only - http://bmjopen.bmj.com/site/about/guidelines.xhtml

			treatment.<br><br>Aged: 46–60 years.			weeks.<br><br>Frequency varied from 5 times per week to weekly and classes were 60–90 minutes.		
Duijts et al, 2011[22]	Evaluate the effect of behavioural techniques and physical exercise on psychosocial functioning and health-related QoL in breast cancer patients and survivors.	42	7164 breast cancer patients, including non-metastatic and metastatic patients during and after treatment.	Mixed during and after treatment.	The review included interventions at the individual and group level. Interventions were conducted face to face, online and by telephone.	Behavioural techniques included psycho-education, problem solving, stress management, CBT, relaxation techniques, social and emotional support.<br><br>Physical interventions included yoga, self-management exercise protocol, aerobic or resistance exercise training and dance movement.<br><br>Intervention duration varied from 1-39 weeks of 3-57 sessions.	Measures included<br><br>SIP, CARES, ABS, EORTC QLQ-C30,, FACT-B, FACT-G, FACT-F, FACT-An, FLIC, QoL-BS, SDS,IFS-CA, QLQ-C30+3	No significant effect of behavioural techniques on HRQoL.<br><br>Physical exercise produced statistically significant but moderate effects on HRQoL.
Ferrer et al, 2011[23]	Examine the efficacy of exercise interventions in improving QOL in cancer survivors, as well as features that may moderate such effects.	91 interventions from 78 studies	3,629 participants; 54% breast cancer, 8% prostate cancer, 2% colorectal cancer, 1% each featured endometrial, head–neck, lymphoma, and ovarian cancer	Survivor was defined as post diagnosis.		Interventions were designed to affect exercise behaviour by comparing low MET vs high MET.<br><br>36% used trained intervention leaders; 56% featured supervised exercise sessions.<br><br>The mean level of	Specific QoL measures included; EORTC, QLQ-30, FACTIT, Quality of life index, FACT-G, FACT-An, FACT-B, FACT H&N, FACT-P, FLIC, CARES-SF, Rotterdam QOL, WHOQOL-BREF.	There was a positive effect of physical interventions on QOL, sustained for delayed follow-up assessment.<br><br>Efficacy increased as the length of intervention decreased, and if exercise was

For peer review only

			survivors, and 32% included mixed diagnosis.<br><br>The mean age was 55.0 years.			targeted aerobic METs was 4.2 (SD=2.2), and the mean level of targeted resistance METs was 2.5 (SD=2.2).<br><br>Duration: 8-26 weeks. The mean length of intervention session was 51.1 min and the mean number of sessions per intervention was 22.8.		supervised.<br><br>Targeted aerobic intensity significantly predicted QOL improvements as a quadratic trend.<br><br>Targeted aerobic METs predicted intervention efficacy.<br><br>Number of sessions, targeted resistance METs, training of facilitators, and inclusion of flexibility content were not significantly related to QOL outcomes.
Fong et al, 2012[9]	Systematically evaluate the effects of physical activity in adult patients after completion of main treatment related to cancer.	39 papers from 34 studies	3769 participants; 65% included breast cancer only, 9% colorectal cancer only, 3% endometrial cancer only, and 27% mixed diagnosis.<br><br>Aged: 39-74 years.	Patients who have completed their main treatment but might be undergoing hormonal treatment.		Exercise interventions included aerobic exercise, resistance or strength training.<br><br>11 were of moderate intensity and 2 were of vigorous intensity.<br><br>Duration: 3 to 60 weeks.<br><br>Frequency ranged from daily to once a week.	QoL measures used include FACT G, FACT-B, FACT-C, EORTC.	Aerobic plus resistance training was significantly more effective than aerobic training alone on general QoL.

For peer review only

For peer review only - http://bmjopen.bmj.com/site/about/guidelines.xhtml

Fors et al, 2011[15]	Determine the effectiveness of psychoeducation, CBT and social support interventions used in the rehabilitation of breast cancer patients.	18 RCTs in 22 publications	3272 breast cancer patients, during and post treatment.	12 weeks to 6 months post treatment.	Provided by computers, internet, video-tapes, trained and/or cancer educators.	The interventions included psychoeducation, CBT and social and emotional support.<br><br>Duration ranged from 2 weeks – 6 months.	In measuring QoL, 17% used FACT-B, 17% used EORTC-QLQ-C30, 5.5% used, 5.5% used QLI, and 5.5% used Euro-QoL-5D.	Psychoeducation showed inconsistent results during and after primary treatment (six trials).<br><br>CBT (4 trials; 6-12 weeks) after primary treatment led to improved QoL. CBT (9-20 weeks) during primary treatment had inconsistent results.
Galveo et al,2005[13]	To present an overview of exercise interventions in cancer patients during and after treatment and evaluate dose-training response considering type, frequency, volume, and intensity of training.	8	1186 mixed cancer patients during and post treatment.<br><br>Aged: 19 - 77 years.	Defined as minimum of one-year post treatment.		Exercise interventions included a cardio exercise programme and mixed training cardio, resistance and flexibility exercises). Intensity level when provided was described as between 60-80% MHR.<br><br>Programme duration was 4-28 weeks. Frequency ranged from twice a week to 5 times per week.	QoL using the Modified Rotterdam QoL Survey.	Contemporary resistance training provides anabolic effects that counteract side effects of cancer treatments, to improve quality of life.

For peer review only

Khan et al, 2012[11]	To assess the effects of organised multidisciplinary rehabilitation during follow-up in women treated for breast cancer.	2	262 breast cancer patients post treatment.	12 months post treatment.	Group based inpatient and home based programme.	Inpatient multi-rehabilitation programme with medical input, psychology, education, dietician, peer support, image consultant and exercise.<br><br>Duration: 3 to 10 weeks of 3 sessions per week.	FACT-B<br><br>Restriction in participation measured using: EORTC-QLQ,	There was 'low level' evidence that multidisciplinary rehabilitation can improve quality of life over 12 months.<br><br>Not possible to suggest optimal frequency, or one type of intervention over another.
McAlpine et al, 2015[16]	Examine the evidence-based literature surrounding the use of online resources for adult cancer patients.	14	2351 lung, prostate, breast, head and neck and mixed cancer patients.	During treatment, in remission, or cured and those who are in the terminal stages of disease.	Web delivered interventions.	Three intervention: Linking the patient with their treating team of healthcare clinicians<br><br>Connecting patients with each other<br><br>Educational resources.<br><br>They were delivered using e-mail, online educational resources, online support groups or message boards, cancer information websites and interactive websites.<br><br>Duration: 4 weeks to 12 months.	Specific measures used were the FACT-B, EORTC QLQ-C30, EPIC-26, 15DHRQoL.	The overall benefit of online interventions for cancer patients is unclear.<br><br>Although there is significant promise, the few interventions that have been rigorously analysed demonstrate mixed efficacy, often of limited duration.

For peer review only - http://bmjopen.bmj.com/site/about/guidelines.xhtml

Mewes et al, 2012[21]	Systematically review effectiveness of multidimensional rehabilitation programs for cancer survivors and cost-effectiveness of cancer rehabilitation in general.	16 papers from 11 RCTs	2175 mixed cancer patients, predominantly breast.	Finished primary treatment with an expected survival duration of at least 1 year.	Inpatient rehabilitation programmes.	Multidimensional rehabilitation defined as consisting of two or more rehabilitation interventions directed at the ICF dimensions. Interventions typically included exercise, CBT, psychotherapy, education and return to work interventions.<br><br>Programme duration: 4 to 15 weeks.	The QoL measures used included, EORTC QLQ-C30, RAND-36, FACT-G, FACT-B+4.	Effect sizes for quality of life were in the range of -0.12 (95% CI: -0.45 to -0.20) to 0.98 (95% CI, 0.69 to 1.29).<br><br>Multi and mono dimension interventions were equally effective.
Mishra et al, 2012[12]	The effectiveness of exercise on overall HRQoL and HRQoL domains among adult post-treatment cancer survivors.	40 trials	3694 mixed cancer patients during and post-treatment. Over 50% included breast cancer patients only.<br><br>Aged: 39 to 68 years	From immediately after surgery to 15 years post treatment.	Settings included a gym, community centre, yoga studio, or university or hospital facility. home-based interventions were included.	Exercise was defined as physical activity causing an increase in energy expenditure in a systematic manner in terms of frequency, intensity, and duration.<br><br>Included prescribed, active exercise formats of aerobic, resistance , stretching or aerobic/resistance combinations.<br><br>Some interventions included modules in psychological or behavioural education.<br><br>Duration ranged from 2 weeks to	Quality of life outcome measures included the EORTC QLQ-C30, FACT-G and specific domains), FACT-G, Cancer Rehabilitation Evaluation System Short Form CARES-SF), Quality of Life for Cancer Patients QoL Index), Medical Outcomes Study Short Form-36 (SF-36).	Exercise has a positive impact on QoL with improvements in global QoL.

For peer review only - http://bmjopen.bmj.com/site/about/guidelines.xhtml

						one year. Frequency varied between daily to once per week. Sessions lasted from 20 to more than 90 minutes.		
Osborn et al, 2006[18]	To investigate the effects of CBT and patient education on quality of life in adult cancer survivors	15 RCTs	1492 mixed cancer patients in total aged 18-84 years.	Defined as beyond the time of diagnosis	Group vs Individual CBT	Interventions included; Group or Individual CBT, Patient Education.<br><br>CBT intervention duration ranged from 3 – 55 weeks. Frequency varied from 1 hour per week to 2 hours per week.<br><br>PE duration ranged from one 20min session - 6 weekly one hour sessions.	QOL measured using the FACT	QOL was improved at short-term and long -term follow up after CBT.<br><br>PE was not related to improved outcomes. Individual interventions were more effective than group.
Spark et al, 2013[19]	The review aimed to determine the proportion of physical activity and/or dietary intervention trials in breast cancer survivors that assessed post-intervention maintenance of outcomes, the proportion of trials that achieved successful post-intervention maintenance of outcomes, and	16 publications from 10 RCTs	1536 breast cancer patients either undergoing or having completed treatment.		Interventions included face to face contact, printed information and telephone counselling.	Interventions were described as physical activity and/or dietary behaviour change aiming to increase aerobic fitness, strength, physical activity.<br><br>Most interventions lasted 1-4 months, with some lasting longer than 6 months.	QoL	More research is needed to identify the best ways of supporting survivors to make and maintain these lifestyle changes. Quality of life specific outcomes from three studies not reported.

For peer review only

	the sample, intervention, and methodological characteristics common among trials that achieved successful postintervention maintenance of outcomes							
Spence et al, 2009[17]	To summarise the literature on the health effects of exercise during cancer rehabilitation.	13 papers from 10 RCTs	327 mixed cancer patients, mostly breast cancer patients. Aged: 16 to 71 years.	'Recently completed' was defined as having completed treatment no more than 6 months prior to enrolment.	Interventions were either supervised exercise programmes or home-based, unsupervised exercise programmes.<br><br>One study employed exercise physiologists to prescribe individually-tailored exercise programmes.	Most interventions were aerobic or resistance-training exercise programmes.<br><br>Most studies prescribed cycling or walking ergometers for the aerobic component. Studies incorporating resistance training prescribed either exercises using machines or resistance bands.<br><br>Duration varied from 2 weeks to 6 months with a frequency of daily exercise to two sessions per week.	QoL was measured in only one study using the Cancer Rehabilitation Evaluation System.	The findings from this review suggest that exercise can provide a variety of benefits for cancer survivors during the rehabilitation period, including an improve QoL.

For peer review only

Zeng et al. 2014[20]	Examine the effectiveness of exercise intervention on the quality of life of breast cancer survivors.	25 studies, 19 included in meta analysis	1073 breast cancer patients aged 18 years or over.	Individuals who had completed active cancer treatment.	Interventions were conducted by telephone and face to face.	Interventions included any type of exercise - aerobic, resistance or combination of aerobic and resistance , yoga, tai chi, aerobic and strength training, aerobic and resistance training and stretching.<br><br>The duration of the intervention ranged from 4 to 52 weeks.<br><br>Time per session varied from 15 to 90minutes, 1 to 5 times per week.	All QOL outcomes including generic, cancer specific: QOL; FACT-g, EORTC-QLQ-C30 and cancer site-specific: FACT-B, EORTC QLQ BR23.	The review found consistent positive effects of exercise interventions in overall QOL and QOL domains. There was a small to moderate effect of interventions on cancer-specific QOL. Single type of exercise intervention general aerobic, yoga or tai chi) had significant differences in QOL score changes in.

For peer review only

For peer review only - http://bmjopen.bmj.com/site/about/guidelines.xhtml

Table 4: Components of the interventions by study

	Cramer et al, 2012[8]	Fong et al, 2012[9]	Buffart et al, 2012[10]	Khan et al, 2012[11]	Mishra et al, 2012[12]	Culos-Reed et al, 2012[14]	Duijts et al, 2011[22]	Ferrer et al, 2011[23]	Fors et al, 2011[15]	Galveo et al, 2005[13]	McAlpine et al, 2015[16]	Mewes et al, 2012[21]	Osborn et al, 2006[18]	Spark et al, 2013[19]	Spence et al, 2009[17]	Zeng et al. 2014[20]
**PHYSICAL**																
Aerobic		•			•		•	•		•					•	•
Aerobic and Resistance					•											•
Resistance					•					•						•
Aquatic exercise					•											
Cardiovascular programme										•						•
Cycling					•					•					•	
Dance movement							•									
Exercise not specified				•								•		•		
METs targeted								•								
Dietary intervention				•										•		
Pilates					•											
Resistance/strength training		•			•		•			•					•	•
Running					•											
Self management exercise							•									
Stretching/Flexibility exercises										•					•	•
Tai Chi					•											•
Treadmill															•	
Walking					•		•			•					•	
Weight training							•									
Yoga/meditation	•		•		•	•	•									•
Qigong					•											
**PSYCHOLOGICAL, EDUCATIONAL & BEHAVIOURAL**																
Body mind							•									
Cognitive behavioural stress therapy							•									
Cognitive behavioural therapy							•		•			•	•			
Cognitive G therapy							•									
Combined psychosexual							•									
Comprehensive coping strategy							•									
Coping skills																
Emotional support							•		•							
Group therapy							•						•			
Guided imagery							•									
Image consultant				•												
Motivational interviewing																

peer review only

Problem solving training								•									
Progressive relaxation training																	
Psychotherapy													•				
Psychosocial therapy								•									
Return to work interventions													•				
Social cognition theory																	
Social support								•		•							
Stress management								•									
Health education								•					•	•			
Psychological education				•				•		•			•	•			
Peer support				•				•				•					
**MODE OF DELIVERY**																	
CD/manuals/videos			•							•							
Face to face				•	•			•							•		
Home based			•	•	•			•							•	•	
Inpatient setting													•				
Multidisciplinary rehabilitation programme				•									•				
Printed information															•		
Support from nurse or voluntary organisations								•		•							
Telephone			•					•							•		•
Web based								•		•		•					

peer review only

Table 5: Reported effect size from meta-analyses in reviews

Authors	Intervention	Type of effect size reported	Reported effect size	Overall finding
**Buffart et al, 2012[10]◊**	Yoga	SMD (7 studies)		
		General QoL	0.37,0.11-0.62	+
Cramer et al, 2012[8]◊	Yoga	SMD (4 studies) Global QoL	0.62, 0.04 to 1.21;	+
Ferrer et al, 2011[23] * $	Exercise	SMD (78 studies)		
		All intervention groups (Immediate FU)	0.34, 0.24 to 0.43	+
		Intervention vs control, adjusted for baseline differences	0.24, 0.12 to 0.35	+
		Delayed FU		
		All intervention groups	0.42, 0.23 to 0.61	+
		Intervention vs Control adjusted for baseline	0.20, -0.058 to 0.46	?
**Fong et al, 2012[9]**	Exercise	2 studies	3.4, 0.4 to 6.4	+
		9 studies	22.1, 16.8 to 27.4,	+
**Mishra et al, 2012[12] ◊**	Exercise	SMD: baseline to after intervention (11 studies)	0.48, 0.16 to 0.81	+
		3-6 month follow up (181 participants)	0.14, -0.38 to 0.66	?
		6 month follow up (115 participants) (2 studies)	0.46, 0.09 to 0.84	+
Zeng et al, 2014[20]	Exercise	Standardised Mean Difference (0verall) (6 studies)	0.70, 0.21, 1.19	+
		Cancer specific (10 studies)	0.38, 0.03 to 0.74	+
Duijts et al, 2011[22] (Exercise)	Exercise i	SMD (or Hedges g for small sample size, with adjustment) (27 studies)	0.298, 0.117 to 0.479, p = 0.001	+
	Behavioural intervention		0.045, -0.044 to 0.135,	?

For peer review only

			p=0.322	
Osborn et al, 2006[18]	CBT	SMD Overall (11 studies)	0.91, 0.38 to 1.44, p<0.01	+
		Short term	1.45, 0.43 to 2.47	+
		Long term	0.26, 0.06 to 0.46	+
		Individual CBT (7 studies)	0.95, -0.367 to 1.536	?
		Individual vs Group CBT (1 study(	0.37, -0.02 to 0.75, p=0.06	?
	Patient Education	(1 study)	-0.04, -0.38 to 0.29, p=0.8	?
			1.99, 0.69 to 3.31	+

*** random effects assumption**

◊ Reviews rated as high quality

$ findings sustained for random or fixed effects, random effects reported.

For peer review only

Overall Effectiveness of Interventions

Meta-analyses were reported in 8 reviews and the effect sizes (as reported in the original reviews) are tabulated Table 5. Of five publications providing meta-analyses of physical activity (not including Yoga), all found convincing positive associations for studies testing response between 1 and 26 weeks. Long term effects were not tested by all, although both Fong, and Zeng, did show persistent effects at six months and a year respectively.[9 20] One review [23] showed uncertain outcome at 3-6 months, although shorter and longer term outcomes were favourable. This review showed equivocal effects when the intervention group was compared with the control group, once adjusted for baseline quality of life and covariates. The two meta-analyses of yoga interventions both showed positive effects,[8 10] as did a review of CBT [18] but there was no evidence of benefit in quality of life with patient education [18] and behavioural interventions.[22]

Two reviews reported effect sizes from individual studies but did not undertake meta-analyses.[15 21] Mewes's review of multidimensional rehabilitation included 10 studies, nine of which had global quality of life outcomes, of which seven showed benefit with effect sizes ranging from 0.04 to 0.99 (no confidence intervals reported).[21] Fors's review included six RCTs only four of which included a quality of life measure;[15] two of these showed positive effect sizes (ranging from 0.56, 95%CI: 0.09 to 1.03; 95% CI: 0.63, 0.11 to 1.18); one showed improved and one a worsening of quality of life as a non-standardised mean score. Five reviews[11 13 16 17 19] did not report meta-analyses nor effect sizes; mostly these provided mean change scores or narrative statements. On the whole these gave a mixed picture, often resorting to sub-group analysis by cancer type or different dimensions of quality of life.

Physical Activity: Summary Findings

Buffart's [10] and Cramer's [8] high quality reviews of 6-12 weeks of yoga in breast cancer patients showed a large increase in general quality of life, a finding that was consistent with a lower quality review.[14] Mishra's[12] high quality review of people with multiple cancers, 50% of whom had breast cancer, found physical activity had a positive effect on global quality of life at three and six months follow up. Fong's[9] high quality review of breast cancer, colorectal, endometrial and mixed cancers, similarly found physical interventions improved general quality of life on average at 13 weeks follow up (range 3-60 weeks). There was inconsistency across the reviews with regard to the types of exercise interventions that were most effective. Fong[9] found aerobic plus resistance training to be significantly more effective than aerobic training alone on many aspects of quality of life. However, Zeng's [20] lower quality review suggested that single types of exercise interventions (general aerobic, yoga or tai chi) were more effective at increasing quality of life at 4-52 weeks after intervention; half of the studies assessed interventions between 8-12 weeks. Duijts[22] study of breast cancer patients found only small effects of physical activity on quality of life (at 8 -26 weeks after intervention); and

For peer review only

Spence's[17] study of mixed but mostly breast cancer patients reported evidence that physical activity improved overall quality of life but only 4 of ten trials maintained the intervention and only a fifth of trials seemed to assess outcome at 3 months and beyond. Zeng's[20] review of breast cancer patients found small but positive benefits of physical activity on overall quality of life. Galveo's[13] review of mixed cancers gave preliminary evidence of positive benefits on a Modified Rotterdam QoL measure, but no overall effects were reported. However, Spark's[19] study of breast cancer patients showed that the impact of physical activity on quality of life was not convincing. Although Spark did not report effect sizes, two of the reviewed studies included quality of life measures, both of which reported effect sizes in the original papers; one showed positive benefits on FACT-G and FACT-B at 8 months (effect sizes 9.8 to 13.4), but not at 24 months follow up; the other showed no significant effects on FACT-G overall, but when the cancer specific FACT G was assessed at six month follow up, there was benefit (4.9, 0.2 to 9.6). Ferrer's[23] study of breast, prostate, endometrial, head and neck, ovarian cancers and lymphoma found small but positive effects of exercise at long term follow up on multiple measures of quality of life. The efficacy of the interventions appeared greater with shorter duration treatments, and if exercise was supervised. Aerobic intensity predicted improvements in quality of life.

Psychological and behavioural interventions: Summary Findings

None of the reviews of psychological and behavioural interventions were classified as high quality. Overall there was no effect on health related quality of life.[22] Fors's[15] review of breast cancer patients showed CBT improved quality of life. No meta-analysis or overall effect sizes were reported due to heterogeneity. Further support for CBT came from Osborn's[18] review of group and individually delivered CBT for mixed cancers; individual interventions were more effective than group based treatment. CBT showed both short-term[15] and long-term improvements in quality of life.[18] Only one of five primary papers in one review assessed the effect of social and emotional

For peer review only - http://bmjopen.bmj.com/site/about/guidelines.xhtml

support as an intervention, four of them finding no effect, and one reporting a significant improvement on quality of life on one measure.[15] There was no evidence that psychosocial education increased quality of life.[15 18]

Multidimensional and Multidisciplinary rehabilitation

Khan's[11] high quality review of breast cancer patients included just two studies, only one of which provided low level evidence that multidisciplinary rehabilitation improved participation and social activities. The other showed no significant effects. Mews's[21] low quality review of mainly breast but also other cancers treated by inpatient multidisciplinary rehabilitation demonstrated no differences between multidimensional and single dimension interventions, with benefits of both on physical outcomes.

Intervention modality

The effectiveness of online educational interventions was unclear. McAlpine's [16 18] review of lung, prostate, head and neck and a smaller number of mixed cancers showed equivocal findings. There were benefits to online education, message boards, but mixed effects for interactive websites, and worse outcome from one study on email interventions.

DISCUSSION

Main findings

Only sixteen reviews were included, reflecting a paucity of evidence and showing much variation in study designs and outcome measures used to indicate quality of life. Low and high quality reviews showed that physical activity improves overall quality of life, but few studies assessed long-term

For peer review only - http://bmjopen.bmj.com/site/about/guidelines.xhtml

outcomes beyond 3 months, and even fewer assessed outcomes beyond a year after the intervention. More focused research and a consistent approach is required to explore the effect on the subdomains of quality of life. [12] Higher quality reviews suggest that aerobic plus resistance training provide maximum improvements in quality of life. [9] There was more evidence on physical rather than psychological or other types of intervention.

None of the included reviews for psychological or behavioural interventions were of high quality. CBT is effective for improving quality of life in the short and long term,[15 18] especially when provided as an individual intervention.[18] There is not much evidence on group versus individual, mono-dimensional versus multi-dimensional or whether multidisciplinary interventions are better or not. Further work is needed to examine these different modalities. Given the accessibility of social media, and its popularity, the findings that email contact was related to poorer quality of life needs further investigation; although interactive websites were beneficial, overall the findings about digital interventions were equivocal.

Limitations

We encountered some methodological limitations in included reviews. Some used multiple outcomes and often mislabelled these as measures of quality of life, and there was no consistent reporting standard.

We did not consider outcomes such as wellbeing or the multiple sub-domains of quality of life to avoid the risk of generating findings due to multiple testing smaller samples. Some reviews included a small number of primary papers.

For peer review only - http://bmjopen.bmj.com/site/about/guidelines.xhtml

We found little overlap between reviews (tabulation available on request), reflecting their specific inclusion and exclusion criteria, and interest in very specific interventions and cancer types.

The physical and psychosocial concerns of patients at different time periods of the cancer experience will vary greatly and interventions effective at one stage may not be suitable for another. Most reviews defined 'survivors' as those who had completed active treatment before the onset of the study.[8 9 13-15 17 20 21 23] Some specified a time frame, from immediately after surgery to 15 years after active treatment.[12] One review defined survival as from diagnosis onwards.[18] Another included terminal stages of cancer.[16] The majority of the reviews incorporated studies combining patients during and post treatment.[8 10 12-16 19 22] These differing definitions of living with and beyond cancer make comparison difficulty, and a standardised approach to trials and reporting of studies is needed.

Interventions were offered to patients based on their diagnosis of cancer, rather than low quality of life, which may have underestimated potential beneficial effects. Future research should consider the effectiveness of interventions targeting people living beyond all types of cancer, and with poor overall quality of life.

Conclusions

The most effective interventions were physical activity based treatments. CBT also had beneficial effects. However, currently there is no standardised study design, outcome selection, or reporting conventions adopted across these reviews. No single intervention can be recommended to those patients with a poor quality of life following cancer treatment as interventions were not targeted on this basis. Future research is needed to help address this.

For peer review only - http://bmjopen.bmj.com/site/about/guidelines.xhtml

What is already known on this topic

Some two million people in the UK are living beyond cancer, and a third report a diminished quality of life or wellbeing. There is a wide variation in NHS provision for these patients, with an unknown effectiveness of the interventions offered.

What this study adds

This review of reviews determines the components of interventions offered, and suggests that interventions are more effective when unidimensional, individual, and focused on one area of quality of life. Interventions that were most effective were exercise based, including yoga. However, CBT also had beneficial effects.

Acknowledgements, competing interests, funding and all other required statements

This review was funded by NIHR-Programme Development Grant: RP-DG-1212-10014

The authors declare not conflicts of interest

The lead and corresponding authors (guarantors) affirm that the manuscript is an honest, accurate, and transparent account of the study being reported; that no important aspects of the study have been omitted; and that any discrepancies from the study as planned (and, if relevant, registered) have been explained. All authors had access to the full data set, and the work was undertaken independently of the funders and sponsor.

Contributor Statement:

KB designed the review, and prepared the review section for the original grant application, which overall was led by PDW. Input on design was provided by all authors (KB, MD, JD, RR, LJ, LB, AM, TC, MT, ST, AK, PW) and PPI experts (Miriam Harris, Adrienne Morgan, and Louisa Smalley) in steering groups during preparation of the funding application and throughout the project; more specific input to design was provided by PDW, SJC. MD and JD were research fellows employed on the grant, and collected the papers, ran the searches and performed the first extraction under supervision by KB. MD and JD undertook the preliminary charting and extraction. KB reviewed all data and checked and completed extraction of the data and identified relevant effect estimates, and led on writing the paper, edited consecutive drafts of the MS, and the produced the final draft. All authors (KB, MD, JD, RR, LJ, LB, AM, TC, MT, ST, AK, PW) contributed to the reviewing the emergent data, and consecutive drafts of the paper for content, the presentation, and discussion about the findings and interpretation at each stage of the review process, as well as the structure of the paper. All authors (KB, MD, JD, RR, LJ, LB, AM, TC, MT, ST, AK, PW) commented on and approved the final version. We thank Miriam Harris, Adrienne Morgan, and Louisa Smalley for helpful analysis and comments in the design, planning and delivery of the research including this review, and in the construction of SURECAN dissemination plans and the design of a future trial.

For peer review only - http://bmjopen.bmj.com/site/about/guidelines.xhtml

The Corresponding Author has the right to grant on behalf of all authors and does grant on behalf of all authors, *a worldwide licence* to the Publishers and its licensees in perpetuity, in all forms, formats and media (whether known now or created in the future), to i) publish, reproduce, distribute, display and store the Contribution, ii) translate the Contribution into other languages, create adaptations, reprints, include within collections and create summaries, extracts and/or, abstracts of the Contribution, iii) create any other derivative work(s) based on the Contribution, iv) to exploit all subsidiary rights in the Contribution, v) the inclusion of electronic links from the Contribution to third party material where-ever it may be located; and, vi) licence any third party to do any or all of the above.

Data Sharing Statement

No additional data are available.

For peer review only

Annex1: Full search strategy

Component 1: Population

#1. Neoplasms mesh) or cancer or cancers or cancerous or carcinoma* or neoplas* or tumor* or tumour* or malignan*

Component 2: Intervention

#2. Counseling mesh) or psychotherapy mesh) or “cognitive therapy” mesh) or “self-help groups” mesh) or “mind body therapies” mesh) or “behavior therapy” mesh) or psychotherapy, group mesh) or meditation, mesh) or “mindfulness” mesh) behaviour therapies, cognitive mesh)

+

#3. counsel*:ti,ab or psychoeducat*:ti,ab or educat*:ti,ab or coping*:ti,ab or psychological*:ti,ab or psychosocail*:ti,ab or psychotherap*:ti,ab or psychoanalytic*:ti,ab AND therap*:ti,ab or treatment*:ti,ab or outcome*:ti,ab or intervention*:ti,ab

+

#4. social: ti,ab or peer: ti,ab or group: ti,ab AND support: ti,ab

+

#5. self: ti,ab AND help: ti,ab

+

#7. cognitive: ti,ab or behav*: ti,ab AND treatment*: ti,ab or therap*: ti,ab

+

#8. “CBT” : ti,ab

+

#9. Family: ti,ab or couple: ti,ab AND therap*: ti,ab

+

#10. meditation: ti,ab or mindfulness: ti,ab

#11. #2+#3+#4+#5+#6+#7+#8+#9+#10

Component 3: Outcome

#12. “quality of life” mesh) or “well being”: ti,ab or “QoL” all fields) or “quality of life”: ti,ab

FULL PICO:

#1 AND #11 AND #12

Filters: Humans, English language, Reviews, Publication Dates, Age group

For peer review only

REFERENCES

1. UK CR. Cancer Survival Statistics. 2015.
2. Jarrett N, Scott I, Addington-Hall J, Amir Z, Brearley S, Hodges L, et al. Informing future research priorities into the psychological and social problems faced by cancer survivors: A rapid review and synthesis of the literature. *European Journal of Oncology Nursing* 2013;17(5):510-20.
3. Miller KD, Triano LR. Medical issues in cancer survivors--a review. *Cancer J* 2008;14(6):375-87.
4. MacmillanCancerSupport. Worried Sick: the emotional impact of cancer. http://www.macmillan.org.uk/documents/getinvolved/campaigns/campaigns/impact_of_cancer_english.pdf: Macmillan Cancer Support 89 Albert Embankment London SE1 7UQ, 2006.
5. Choices N. Complementary and Alternative Medicine. http://www.nhs.uk/Livewell/complementary-alternative-medicine/Pages/complementary-alternative-medicines.aspx, Accessed October 2016.
6. Kung J CF, Cajulis OO, Avezova R, Kossan G, Chew L, Maida CA. . From systematic reviews to clinical recommendations for evidence-based health care: validation of revised assessment of multiple systematic reviews (R-AMSTAR) for grading of clinical relevance. *The open dentistry journal.* 2010;16(4):1.
7. Mays N, Pope C, Popay J. Systematically reviewing qualitative and quantitative evidence to inform management and policy-making in the health field. *Journal of health services research & policy* 2005;10 Suppl 1:6-20.
8. Cramer H, Lange S, Klose P, Paul A, Dobos G. Yoga for breast cancer patients and survivors: a systematic review and meta-analysis. *BMC Cancer* 2012;12(1):1-13.
9. Fong DYT, Ho JWC, Hui BPH, Lee AM, Macfarlane DJ, Leung SSK, et al. Physical activity for cancer survivors: meta-analysis of randomised controlled trials. *BMJ* 2012;344.
10. Buffart LM, van Uffelen JG, Riphagen II, Brug J, van Mechelen W, Brown WJ, et al. Physical and psychosocial benefits of yoga in cancer patients and survivors, a systematic review and meta-analysis of randomized controlled trials. *BMC Cancer* 2012;12(1):1-21.
11. Khan F, Amatya B, Ng L, Demetrios M, Zhang NY, Turner-Stokes L. Multidisciplinary rehabilitation for follow-up of women treated for breast cancer. *Cochrane Database of Systematic Reviews* 2012(12).
12. Mishra SI, Scherer RW, Snyder C, Geigle PM, Berlanstein DR, Topaloglu O. Exercise interventions on health-related quality of life for people with cancer during active treatment. *Cochrane Database of Systematic Reviews* 2012(8).
13. Galvão DA NR. Review of exercise intervention studies in cancer patients. *Journal of clinical oncology* 2005(4):899-909.
14. Culos-Reed SN MM, Sohl SJ, Jesse MT, Zahavich AN, Danhauer SC. Yoga & cancer interventions: a review of the clinical significance of patient reported outcomes for cancer survivors. . *Evidence-Based Complementary and Alternative Medicine.* 2012.
15. Fors EA, Bertheussen GF, Thune I, Juvet LK, Elvsaas I-KØ, Oldervoll L, et al. Psychosocial interventions as part of breast cancer rehabilitation programs? Results from a systematic review. *Psycho-Oncology* 2011;20(9):909-18.
16. McAlpine H, Joubert L, Martin-Sanchez F, Merolli M, Drummond KJ. A systematic review of types and efficacy of online interventions for cancer patients. *Patient Education and Counseling* 2015;98(3):283-95.
17. Spence RR, Heesch KC, Brown WJ. Exercise and cancer rehabilitation: A systematic review. *Cancer Treatment Reviews* 2010;36(2):185-94.
18. Osborn RL DA, Feuerstein M. Psychosocial interventions for depression, anxiety, and quality of life in cancer survivors: meta-analyses. The International Journal of Psychiatry in Medicine.

For peer review only

2006;36(1):13-34. Psychosocial interventions for depression, anxiety, and quality of life in cancer survivors: meta-analyses. . *The International Journal of Psychiatry in Medicine.* 2006;36 (1):13-34.

19. Spark LC, Reeves MM, Fjeldsoe BS, Eakin EG. Physical activity and/or dietary interventions in breast cancer survivors: a systematic review of the maintenance of outcomes. *Journal of Cancer Survivorship* 2013;7(1):74-82.
20. Zeng Y, Huang M, Cheng ASK, Zhou Y, So WKW. Meta-analysis of the effects of exercise intervention on quality of life in breast cancer survivors. *Breast Cancer* 2014;21(3):262-74.
21. Mewes JC SL, IJzerman MJ, Van Harten WH. . Effectiveness of multidimensional cancer survivor rehabilitation and cost-effectiveness of cancer rehabilitation in general: a systematic review. *The oncologist* 2012.
22. Duijts SFA, Faber MM, Oldenburg HSA, van Beurden M, Aaronson NK. Effectiveness of behavioral techniques and physical exercise on psychosocial functioning and health-related quality of life in breast cancer patients and survivors—a meta-analysis. *Psycho-Oncology* 2011;20(2):115-26.
23. Ferrer RA, Huedo-Medina TB, Johnson BT, Ryan S, Pescatello LS. Exercise Interventions for Cancer Survivors: A Meta-Analysis of Quality of Life Outcomes. *Annals of Behavioral Medicine* 2011;41(1):32-47.

For peer review only

BMJ Open

A Review of Systematic Reviews of non-pharmacological interventions to improve quality of life in cancer survivors.

Journal:	*BMJ Open*
Manuscript ID	bmjopen-2017-015860.R1
Article Type:	Research
Date Submitted by the Author:	07-Aug-2017
Complete List of Authors:	Duncan, Morvwen; Barts and The London School of Medicine & Dentistry Queen Mary University of London, Centre for Psychiatry at the Wolfson Institute of Preventive Medicine<br>Moschopoulou, Elisavet; Barts and The London School of Medicine & Dentistry Queen Mary University of London, Centre for Psychiatry at the Wolfson Institute of Preventive Medicine<br>Herrington, Eldrid; Barts and The London School of Medicine & Dentistry Queen Mary University of London, Centre for Psychiatry at the Wolfson Institute of Preventive Medicine<br>Deane, Jennifer; Barts and The London School of Medicine & Dentistry Queen Mary University of London, Centre for Psychiatry at the Wolfson Institute of Preventive Medicine<br>Roylance, Rebecca ; University College London<br>Jones, Louise; University College Medical School, Marie Curie Palliative Care Unit, UCL Mental Health Sciences Unit<br>Bourke, Liam; University of Sheffield Medical School<br>Morgan , Adrienne ; Queen Mary University of London - Charterhouse Square Campus, Barts Cancer Institute<br>Chalder, Trudie; King's College London, Psychological Medicine<br>Thaha, Mohamed; Queen Mary's University of London, National Centre for Bowel Research & Surgical Innovation<br>Taylor, Stephanie; Barts and The London School of Medicine & Dentistry Queen Mary University of London, Centre for Psychiatry at the Wolfson Institute of Preventive Medicine<br>Korszun, Ania; Barts and The London School of Medicine and Dentistry, Psychiatry<br>White, Peter; Barts and the London School of Medicine, Queen Mary University of London, Centre for Psychiatry<br>Bhui, Kamaldeep; Barts and The London School of Medicine & Dentistry Queen Mary University of London, Centre for Psychiatry at the Wolfson Institute of Preventive Medicine
**Primary Subject Heading**:	Oncology
Secondary Subject Heading:	Public health, Patient-centred medicine
Keywords:	Adult oncology < ONCOLOGY, Quality of life, Psychosocial interventions

For peer review only

A Review of Systematic Reviews of non-pharmacological interventions to improve quality of life in cancer survivors.

Morvwen Duncan, Elisavet Moschopoulou, Eldrid Herrington, Jennifer Deane, Rebecca Roylance, Louise Jones, Liam Bourke, Adrienne Morgan, Trudie Chalder, Mohamed A. Thaha, Stephanie Taylor, Ania Korszun, Peter White, *Kamaldeep Bhui on behalf of SURECAN Investigators.

Morvwen Duncan

Research Assistant

Academic Psychological Medicine, Wolfson Institute of Preventive Medicine, Barts and the London School of Medicine and Dentistry, Queen Mary University of London, EC1A 7BE

Elisavet Moschopoulou

Postgraduate Researcher

Centre for Psychiatry, Barts and The London School of Medicine and Dentistry, Queen Mary University of London, Old Anatomy Building, Charterhouse Square, London EC1M 6BQ

Eldrid Herrington

Honorary Senior Research Fellow

Blizard Institute, National Bowel Research Centre, Queen Mary University of London, 2 Newark Street, London E1 2AT

Public Patient representative

Department of Colorectal Surgery, The Royal London Hospital, Barts Health NHS Trust, Whitechapel, London E1 1BB

Jennifer Deane

Research Assistant

Academic Psychological Medicine, Wolfson Institute of Preventive Medicine, Barts and the London School of Medicine and Dentistry, Queen Mary University of London, EC1A 7BE

Rebecca Roylance

Consultant Medical Oncologist and Honorary Senior Lecturer

University College Hospitals NHS Foundation Trust and UCLH Biomedical Research Centre, 149 Tottenham Court Road, London W1T 7DN

Louise Jones

Clinical Senior Lecturer

Marie Curie Palliative Research Department, Division of Psychiatry, UCL, 149 Tottenham Court Road, London W1T 7NF

Liam Bourke

Reader in Clinical Science

For peer review only - http://bmjopen.bmj.com/site/about/guidelines.xhtml

Sheffield Hallam University, Howard Street, Sheffield S1 1WB.

Adrienne Morgan

Honorary Senior Lecturer

Centre for Tumour Biology, Barts Cancer Institute - Queen Mary University of London, Old Anatomy Building, London EC1M 6BQ

Trudie Chalder

Professor of Cognitive Behavioural Psychotherapy

Department of Psychological Medicine, King's College London, Denmark Hill, King's College, London, SE59RJ

Mohamed A Thaha

Senior Lecturer & Consultant in Colorectal Surgery

Blizard Institute, National Bowel Research Centre,
Barts and the London School of Medicine & Dentistry, Queen Mary University of London, 2 Newark Street, London E1 2AT.
Department of Colorectal Surgery, The Royal London Hospital, Barts Health NHS Trust, Whitechapel, London E1 1BB

Stephanie Taylor

Professor in Public Health and Primary Care

Centre for Primary Care and Public Health, Blizard Institute, Barts and The London School of Medicine and Dentistry, London, E1 2AB

Ania Korszun

Professor of Education and Psychiatry

Wolfson Institute of Preventive Medicine, Barts and the London School of Medicine and Dentistry, Queen Mary University of London, EC1M 6BQ

Peter White

Professor of Psychological Medicine

Wolfson Institute of Preventive Medicine, Barts and the London School of Medicine and Dentistry, Queen Mary University of London, EC1A 7BE

* Kamaldeep Bhui

Professor of Cultural Psychiatry & Epidemiology

Wolfson Institute of Preventive Medicine, Barts and the London School of Medicine and Dentistry, Queen Mary University of London, EC1A 7BE

& SURECAN Research Group

*Correspondence to: Kamaldeep Bhui, k.s.bhui@qmul.ac.uk, 020 7882 2012

For peer review only - http://bmjopen.bmj.com/site/about/guidelines.xhtml

Key Words: *Cancer, quality of life, interventions*

Word Count:

Paper excluding abstract, tables and references: 4795

Abstract: 296

For peer review only

For peer review only - http://bmjopen.bmj.com/site/about/guidelines.xhtml

ABSTRACT

Objectives

Over two million people in the UK are living with and beyond cancer. A third report diminished quality of life.

Design

A review of published systematic reviews to identify effective non-pharmacological interventions to improve the quality of life of cancer survivors

Data Sources

Databases searched until May 2017 included PubMed, Cochrane Central, EMBASE, MEDLINE, Web of Science, the Cumulative Index to Nursing and Allied Health Literature (CINAHL) and Psych INFO.

Study selection

Published systematic reviews of randomised trials of non-pharmacological interventions for people living with and beyond cancer were included; included reviews targeted patients aged over 18. All participants had already received a cancer diagnosis. Interventions located in any healthcare setting, home or online were included. Reviews of alternative therapies or those non-English reports were excluded. Two researchers independently assessed titles, abstracts, the full text of papers, and independently extracted the data.

Outcomes

The primary outcome of interest was any measure of global (overall) quality of life.

Analytic methods

Quality assessment (AMSTAR) and narrative synthesis, evaluating effectiveness of non-pharmacological interventions and their components.

For peer review only

Results

Of 14,430 unique titles, 21 entered the review of reviews. There was little overlap in the primary papers across these reviews. 13 reviews covered mixed tumour groups, seven focused on breast cancer, and one focused on prostate cancer. Face-to-face interventions were often combined with online, telephone and paper-based reading materials. Interventions included physical, psychological or behavioural, multidimensional rehabilitation and online approaches. Yoga specifically, physical exercise, more generally, cognitive behavioural therapy (CBT) and mindfulness-based stress reduction (MBSR) programmes showed benefit in terms of quality of life.

Conclusions

Exercise-based interventions were effective in the short (less than 3-8 months) and long term. CBT and MBSR also showed benefits, especially in the short term. The evidence for multidisciplinary, online, and educational interventions was equivocal.

For peer review only

ARTICLE SUMMARY

Strengths and limitations of this study

- This systematic evidence synthesis provides information to carers, patients and professionals about the effective elements of non-pharmacological interventions in cancer survivors.
- Longer term studies are needed and studies of greater methodological quality that adopt similar reporting standards.
- Definitions of survivor varied and more studies are needed for different types of cancer, and specifically for patients who have poor quality of life.
- More studies are needed that investigate educational, online and multidisciplinary team based interventions.
- This review has some limitations in the methodology. Studies not in English and grey literature were not included. This was a review of reviews: we did not review individual studies focussed on specific cancers or stage and we did not re-assess the quality of the primary studies included in each review.

For peer review only

For peer review only - http://bmjopen.bmj.com/site/about/guidelines.xhtml

INTRODUCTION

Advances in public awareness, early detection and improved treatments mean that more people are now living with and beyond cancer. For example, Cancer Research UK reports that 50% of people diagnosed with cancer in England and Wales survive 10 years or more, and survival rates have doubled over the last 40 years.[1] This group of survivors includes people at various stages of active treatment, and those in remission, who are gradually restoring their social and occupational roles.

A significant proportion of cancer survivors experience poor quality of life.[2] The main causes of poor quality of life include depression, anxiety, distress, fear of recurrence, lower levels of social support; impacts on relationships, family, and social function; psychological and social needs, and problems coping.[2 3] The process of diagnosis and treatment is traumatic and disruptive. It is not unusual for cancer patients to experience distress. Common experiences for those living with and beyond cancer include reduced physical ability, fatigue, changes in sexual activity and developing other medical conditions that affect function for many years.[2 3] If a person is suffering from fatigue, depression or anxiety they are understandably less motivated to visit friends or engage in social activities; the strain on marital relationships may lead to a loss of support: 25% of people who experience difficulties have broken up with their partner as a result of cancer.[3 4] Thus, the effects of cancer extend beyond the diagnostic and active treatment phases. This review aims to gather the evidence for practitioners, patients and their carers about effective non-pharmacological interventions to improve quality of life in cancer survivors. We sought to summarise the effectiveness of non-pharmacological interventions in cancer survivors as part of a NIHR funded programme development grant to inform the design and delivery of a full programme grant.

For peer review only - http://bmjopen.bmj.com/site/about/guidelines.xhtml

METHODS

This review of reviews examined existing systematic reviews of non-pharmacological interventions that include information on quality of life of those living with and beyond cancer.

Inclusion and exclusion criteria

The study included any systematic reviews that explicitly reported randomised controlled trials. Inclusion criteria were organised in accordance with the PICO reporting structure (see Table 1). The population of interest was people living with and beyond cancer, who were aged 18 years or more, and who had received their cancer diagnosis as adults.

We defined non-pharmacological interventions as those that did not involve any drug or medicine, but they could include educational, behavioural, psychosocial approaches or physical activity; we excluded complementary and alternative therapies as defined by the NHS Choices resource.[5] However, we included physical activity and psychological approaches that were part of yoga based interventions after consulting with patients in the development of the review. Comparators were not specified for the purpose of the inclusion criteria of the review of reviews, but comparators reported in the original reviews were considered in the analysis.

The primary outcome was quality of life (QoL) defined by physical, psychological and social functioning. We reported on studies that used an established and validated measure of global or overall QoL; some of these are cancer specific. In the literature, the terms 'Quality of Life' and 'Health Related QoL' are used interchangeably; therefore both are included under the term 'QoL' in this review. The study settings included any healthcare venue, such as hospital inpatient or

For peer review only - http://bmjopen.bmj.com/site/about/guidelines.xhtml

outpatient services, community services and also included home and remote e-technology based interventions.

Table 1: Application of the PICO search strategy

**Population**	Participants living beyond cancer, who have completed active treatment with curative intent; aged 18 or more who received their cancer diagnosis in adulthood.
**Intervention**	Non-pharmacological interventions. Psychological, social and physical activity, excluding complementary and alternative therapies or medicines. Including yoga interventions with meditation, activity or mindfulness.
**Outcomes**	Quality of life.
**Setting**	Any healthcare setting: hospital (in-patient or outpatient), community or remote (e.g. using e-technology).
**Study Design**	Systematic reviews that had explicitly searched for RCTs. To be classified as a systematic review the following criteria were met:<br>- clear inclusion criteria<br>- a systematic search strategy<br>- a screening procedure to identity relevant studies<br>- systematic data extraction and analysis procedures for RCTs

Data sources

We searched the following databases: PubMed, Cochrane Central, EMBASE, MEDLINE, Web of Science, The Cumulative Index to Nursing and Allied Health Literature (CINAHL) and Psych INFO. The final search was from inception to May 2017 and is shown in Annex 1. We consulted experts in the field to assess completeness of the list of identified reviews, and where necessary, contacted authors to secure the full text versions.

For peer review only - http://bmjopen.bmj.com/site/about/guidelines.xhtml

Study selection

Two authors (MD, JD) independently screened all titles and abstracts of studies identified by the search strategy against inclusion and exclusion criteria, and, when eligibility was determined, the full text was read. Discrepancies around inclusion were resolved by discussion or in consultation with a third author when required (KB). We searched the reference lists of all included reviews to identify any further relevant reviews. The research team was not blinded to authors. Citations were downloaded and managed in an Endnote library.

Data extraction

Two authors (EM, EH) independently extracted data from each of the eligible reviews into a purpose built, pre-designed, structured template. The data extraction forms were then summarised in a table and reviewed independently by a third reviewer (KB) Extracted data included the following information:

- Publication details: author, year, title, journal, country, format of publication.
- Study characteristics: number of primary studies, total number of participants, range of publication dates, gender, age range of participants and socioeconomic data, primary cancer site, length of time since final cancer treatment and type of treatment.
- Intervention design and evaluation: setting, description of the intervention and its components: physical components, psychosocial components, educational components; duration of intervention, follow up, number of treatment contacts, type of practitioner providing treatment, mode of delivery of intervention, and any outcomes.
- Documents: Availability of treatment manuals.
- Results: Main outcome measures, secondary outcome measures, narrative findings, adherence levels, patient satisfaction, effect sizes against intervention components.

For peer review only - http://bmjopen.bmj.com/site/about/guidelines.xhtml

For peer review only

Assessment of methodological quality of included reviews

The methodological quality of the systematic reviews was evaluated using AMSTAR[6], a measurement tool for the assessment of multiple systematic reviews that has good reliability and validity (Table 2). The AMSTAR checklist used can be found here: https://amstar.ca/Amstar_Checklist.php.

Data analysis and narrative synthesis

The intervention components were listed, followed by a narrative synthesis.[7] This included understanding components of the interventions, exploring patterns of findings across studies and within primary reviews, and giving greater weight to studies of higher quality in the interpretation of the findings, especially if there were contradictions between the findings of reviews. Ultimately, the purpose was to put into text format the key findings from the most robust evidence available, to guide treatment and future research recommendations. The synthesis set out reported effect sizes across studies, means and standard deviation. Meta-analysis was not undertaken, due to heterogeneity of methods, outcomes, and absence of reported effect sizes (10 reviews did not provide effect sizes). The publications were segmented into those reporting meta-analyses to which the greatest weighting was given in the synthesis; some reviews did not undertake or report meta-analyses but rather reported each study, trends and the range of effect sizes; a third group reported no effect sizes but provided narrative statements.

Patient and Public Involvement

Patients and carers (and respective organisations) were involved in the design and development of the programme development grant application (from which this review is one output). Patients and carers attended all the steering group meetings and were an integral part of the research team, commenting on and critiquing the inclusion and exclusion criteria, outcome selection, and the acceptability and likely value of interventions. As part of the steering group, they received and commented on study progression, emergent findings, and reports. They are integral to the

For peer review only - http://bmjopen.bmj.com/site/about/guidelines.xhtml

dissemination plans including sharing the publication, but also helping craft lay summaries of the overall research project and key findings. A Public-Patient representative (EH) performed the data extraction together with research and clinical colleagues and co-authored and edited the review. Public Patient representatives were also part of the steering group and informed the design and delivery of the review.

RESULTS

Study selection

Electronic database searches yielded 14,430 unique reviews. From this 290 were included from the title search, followed by 47 from the abstract search. After scrutinising the full texts, 21 of eligible published reviews entered this review (Figure 1). The 26 studies excluded studies are listed in an online supplementary file. The quality scores are shown in Table 2.

For peer review only

For peer review only - http://bmjopen.bmj.com/site/about/guidelines.xhtml

Figure 1: PRISMA flow diagram of study selection

FIGURE 1 ABOUT HERE

Table 2: AMSTAR, tool for the assessment of multiple systematic reviews

Review	AMSTAR Score*	Quality Rating
Bourke et al, 2015	3	Low
Buffart et al, 2012	6	Moderate
Cramer et al, 2012	9	High
Culos-Reed et al, 2012	3	Low
Duijts et al, 2010	4	Moderate
Ferrer et al, 2011	8	High
Fong et al, 2012	8	High
Fors et al, 2011	5	Moderate
Galvão et al, 2005	2	Low
Gerritsen and Vincent 2015	6	Moderate
Huang et al, 2015	8	High
Khan et al, 2012	10	High
McAlpine et al, 2015	5	Moderate
Mewes et al, 2012	5	Moderate
Mishra et al, 2012	10	High
Osborn et al, 2006	7	Moderate
Smits et al, 2015	8	High
Spark et al, 2013	6	Moderate
Spence et al, 2010	5	Moderate
Zachariae et al. 2015	5	Moderate
Zeng et al, 2014	6	Moderate

**The maximum score on AMSTAR is 11 and scores of 0-3 indicate that the review is of low quality, 4-7 of moderate quality; and of 8-11 as high quality*

For peer review only

Study characteristics

The types of interventions, settings, cancer type, measures of quality of life, and the key narrative findings are reported in Table 3.

Participants

The number of patients included in the reviews ranged from 262[8] to 7164.[9] 13 reviews covered mixed tumour groups,[10-22] seven specifically focused on breast cancer,[8 9 23-27] and one on prostate cancer.[28]

Intervention Type and Components

Face-to-face delivery of interventions was often combined with online delivery (three reviews)[9 24 28]; others included telephone communication (five reviews)[9 11 23 25 26] and printed information (two reviews).[11 25] Four reviews included interventions that provided supplementary compact discs, manuals or video tools.[11 23 24 28] Two reviews were from inpatient rehabilitation.[8 18] None of the reviews reported the use of structured manuals, and interventions were often not fully described or broken down into different components, nor was there attention to a mechanism or theory of change.

Ten of the reviews focused on physical interventions,[10 12 13 16 19-21 25 26 28] and three focused on yoga; [11 14 23] four reviews were of psychosocial or behavioural interventions; [9 17 24 27] and one review focused on online interventions including connecting patients and online education (see Tables 3 & 4).[15] One review compared multi-dimensional versus mono-dimensional interventions [18] and one tested multidisciplinary rehabilitation models. [8] Finally, one review focused on the effects of expressive writing. [22] The duration and frequency of the interventions varied greatly from a single 20 minute session [17] to 60 weekly sessions. [10]

For peer review only - http://bmjopen.bmj.com/site/about/guidelines.xhtml

The most common component of physical interventions was aerobic exercise, [9 10 12 13 16 19 26] and resistance/strength training. [9 10 12 13 16 26] Psychological education [8 9 17 18 24] and cognitive behaviour therapy [9 17 18 24] were the most utilised psychological and educational interventions. Peer support was often used as a psychological and a behavioural intervention. [8 9 15] Components of the interventions were thematically organised into two groups (see Table 4 for a more detailed itemisation): biological or physical actions (19 types of activity or diet change) and psychological, behavioural or educational (24 types of intervention about mind and body including cognitive behavioural therapy, mindfulness-based stress reduction, psychosexual therapy, supporting existing coping methods, emotional support, relaxation, psychotherapy and psychosocial therapy, and interventions focusing on social support, guided imagery, self-management, use of peer support, bibliotherapy, telephone and web-based interventions, return to work interventions).

For peer review only

For peer review only - http://bmjopen.bmj.com/site/about/guidelines.xhtml

Table 3: Characteristics of included reviews

Review	Aims of review	Number of primary studies	Participants	Definition of 'survivor'	Setting	Intervention, duration and frequency	Outcome - QoL measures	Narrative findings
Buffart et al, 2012	Systematic review of RCTs and meta-analysis of the effects of yoga in cancer patients and survivors.	16 publications/13 RCTs	744 breast cancer patients and 39 lymphoma cancer patients during and after treatment. Mean age range: 44-63 years.	Patients during and after treatment.	Face to face, with supplementary booklets and audiotapes of exercises for home practice	All included a yoga program led by experienced yoga instructors with physical poses (asanas), breathing techniques, (pranayama), and relaxation or meditation (savasana or dhanya). Programme duration: 6 weeks to 6 months.	FACT-G, SF-36, EORTC QLQ-C30, EORTC QLQ-C30, FLIC	Yoga has strong beneficial effects on distress, anxiety and depression, moderate effects on fatigue, general HRQoL, emotional function and social function, small effects on functional well-being, and no significant effects on physical function and sleep disturbances.
Bourke et al, 2015	To evaluate the evidence from RCTs of supportive interventions designed to improve prostate cancer-specific quality of life.	20 RCTs	2,654 prostate cancer survivors	Patients during and after treatment.	Group or face to face, online or with supplementary audiotapes	Lifestyle interventions including exercise interventions, diet interventions or a combination of exercise and diet. Multidisciplinary group education or online education and support. Enhanced standard care interventions and cognitive behavioural interventions. Varied durations and follow up frequencies.	FACT-P, QLQ-PR25, EPIC, EPIC-26, UCLA-PCI, PCa-QoL	Supervised and individually tailored patient-centred interventions such as lifestyle programmes are beneficial.

For peer review only

Cramer et al, 2012	To systematically assess and meta-analyse the evidence for the effects of yoga on HRQoL nd psychological health in breast cancer patients and survivors.	12 RCTs were included in the qualitative synthesis and 10 of them were included in the meta-analysis	742 breast cancer patients during or after treatment. Mean age range: 44-63 years.	Those who had completed active treatment before the onset of the study.	Face to face, with supplementary audio and video tools or telephone calls.	Yoga interventions including Iyengar yoga, Yoga of Awareness, Viniyoga, restorative yoga, yoga based on Patanjali's yoga tradition, Yoga in Daily Life, integrated yoga and hatha yoga. Duration: 1 week to 6 months. Frequency varied from daily sessions to weekly.	FACT-G, FACT-B, FACIT-Sp, SF-36, SF-12, FLIC, EORTC QLQ-C30	There is moderate evidence for the short-term effect of yoga on global HRQoL. However these short-term effects could not be clearly distinguished from bias.
Culos-Reed et al, 2012	To determine the clinical significance of patient-reported outcomes from yoga interventions conducted with cancer survivors.	13 studies/ 7 RCTs	474 mixed cancer patients. The majority were breast cancer patients during and after treatment. RCTs: sample size in the treatment group at time 2 ranged from 13-45 patients. Mean age range: 46–60 years.	Patients both on and off treatment.	Face to face	Yoga styles included hatha, integral, iyengar, tibetan, viniyoga, and vivekananda. Duration: 6 to 26 weeks. Frequency varied from 5 times per week to weekly and classes were 60–90 minutes.	SF-36, EORTC QLQ-C30, FACT-B, FACT-G, SF-12, NHP	Yoga for cancer survivors results in clinically significant improvements in overall HRQoL, as well as in its mental and emotional domains.

For peer review only - http://bmjopen.bmj.com/site/about/guidelines.xhtml

Duijts et al, 2011	Evaluate the effect of behavioural techniques and physical exercise on psychosocial functioning and HRQoL in breast cancer patients and survivors.	56 RCTs	>7,000 breast cancer patients, including non-metastatic and metastatic patients during and after treatment. Participants' ages were not specified.	Patients during and after treatment.	Face to face, online or by telephone, individually or at group level	Behavioural techniques included psycho-education, problem solving, stress management, CBT, relaxation techniques, social and emotional support.<br>Physical interventions included yoga, self-management exercise protocol, aerobic or resistance exercise training and dance movement.<br>Intervention duration varied from 1-56 weeks of 3-56 sessions.	SIP, CARES, ABS, EORTC QLQ-C30, FACT-B, FACT-G, FACT-F, FACT-An, FLIC, SF-12, SF-36, QoL-BC, GHQ, SDS, IFS-CA, VAS	No significant effect of behavioural techniques on HRQoL.<br>Physical exercise produced statistically significant but moderate effects on HRQoL.
Ferrer et al, 2011	To examine the efficacy of exercise interventions in improving quality of life in cancer survivors, as well as features that may moderate such effects.	78 studies/ 43 RCTs	3,629 participants: 54% breast cancer, 8% prostate cancer, 2% colorectal cancer, 1% each featured endometrial, head–neck, lymphoma, and ovarian cancer survivors, and 32% included mixed diagnosis. 2,432 patients participated in the RCTs.<br>Mean age was 55 years.	Survivor was defined as post diagnosis.	Supervised or unsupervised	Interventions were designed to affect exercise behaviour by comparing low vs high exercise intensity.<br>36% used trained intervention leaders; 56% featured supervised exercise sessions.<br>The mean level of targeted aerobic METs was 4.2 (SD=2.2), and the mean level of targeted resistance METs was 2.5 (SD=2.2).<br>Duration: 8-26 weeks. The mean length of intervention session was 51.1 mins and the mean number of sessions per intervention was 22.8.	EORTC QLQ-30, SF-36, FACTIT, Quality of Life Index, FACT-G, FACT-An, FACT-B, FACT H&N, FACT-P, FLIC, CARES-SF, Rotterdam QOL, WHOQOL-BREF.	There was a positive effect of physical interventions on QoL, sustained for delayed follow-up assessment.<br>Efficacy increased as the length of intervention decreased, and if exercise was supervised.<br>Targeted aerobic intensity significantly predicted QoL improvements as a quadratic trend.<br>Targeted aerobic METs predicted intervention efficacy.<br>Number of sessions, targeted resistance METs, training of facilitators, and inclusion of flexibility content were not significantly related to QoL outcomes.

For peer review only - http://bmjopen.bmj.com/site/about/guidelines.xhtml

Fong et al, 2012	To systematically evaluate the effects of physical activity in adult patients after completion of main treatment related to cancer.	34 RCTs	3,769 participants; 65% included breast cancer only, 9% colorectal cancer only, 3% endometrial cancer only, and 27% mixed diagnosis. Mean age range: 39-74 years.	Patients who have completed their main cancer treatment but might be undergoing hormonal treatment.	Face to face	Exercise interventions included aerobic exercise, resistance or strength training. Duration: 3 to 60 weeks. Frequency ranged from daily to once a week.	FACT G, FACT-B, FACT-C, EORTC, SF-36	Physical activity was shown to be associated with clinically important positive effects on quality of life. Aerobic plus resistance training was significantly more effective than aerobic training alone on general QoL.
Fors et al, 2011	To determine the effectiveness of psychoeducation, cognitive behavioural therapy (CBT) and social support interventions used in the rehabilitation of breast cancer patients.	18 RCTs	3,272 breast cancer patients, during and post treatment. Age range not specified.	Patients who have finished surgery and adjuvant treatment.	Online, face to face or by telephone or by using print material, individually or in a group	Psychoeducation, CBT and social and emotional support. Duration ranged from 2 weeks – 6 months.	FACT-B, FACT-G, EORTC-QLQ-C30, QoL-BC, QLI, EuroQoL-5D, QoQ-C33 Global	Psychoeducation showed inconsistent results during and after primary treatment. CBT after primary treatment (6-12 weeks) led to improved QoL. CBT during primary treatment had inconsistent results.

For peer review only - http://bmjopen.bmj.com/site/about/guidelines.xhtml

Galvao et al, 2005	To present an overview of exercise interventions in cancer patients during and after treatment and evaluate dose-training response considering type, frequency, volume, and intensity of training along with physiological outcomes.	26 studies/ 9 RCTs	1,186 mixed cancer patients during and post treatment. 458 patients participated in the RCTs. Age range: 14 - 65 years.	Patients during and after treatment.	Face to face	Exercise interventions included a cardiovascular exercise programme and mixed training (cardio, resistance and flexibility exercises). Intensity level when provided was described as between 60-80% maximum heart rate (MHR). Programme duration was 4-28 weeks. Frequency ranged from twice a week to 5 times per week.	Modified Rotterdam QoL Survey.	Contemporary resistance training provides anabolic effects that counteract side effects of cancer treatments, to improve quality of life.
Gerritsen and Vincent, 2015	To evaluate the effectiveness of exercise in improving QoL in patients with cancer, during and after treatment.	16 RCTs	1,845 mixed, breast, lymphoma, colorectal, prostate and lung cancer patients. Aged: 18-79 years	Patients during or after treatment.	Home-based or outdoors, supervised or unsupervised	Exercise modalities included walking, cycling, strength training, swimming, stability training and elliptical training ranging from twice a week to five times a week. The duration ranged from 3 weeks to 16 months.	EORTC-QLQ, FACT-An, FACT-B, FACT-C, FACT-G, FACT-P, SF-36, MCS/PCS	Exercise has a direct positive impact on cancer patients' QoL, during and following medical intervention

For peer review only - http://bmjopen.bmj.com/site/about/guidelines.xhtml

Huang et al, 2015	Meta-analysis to evaluate the benefits of mindfulness-based stress reduction on psychological distress among breast cancer survivors.	9 studies/ 4 RCTs	964 breast cancer survivors. 812 patients participated in the RCTs. Mean age range: 49 - 57.5	Women diagnosed with breast cancer.	Setting not specified	8-week mindfulness based stress reduction program. One study used a 6 week formula.	FACT-B	Mindfulness based stress reduction programmes showed a positive effect in improving psychological function and overall QoL of breast cancer survivors.
Khan et al, 2012	To assess the effects of organised multidisciplinary rehabilitation during follow-up in women treated for breast cancer.	2 RCTs	262 breast cancer patients after treatment. All women were older than 49 years except for two.	At least 12 months after completion of definitive cancer treatment.	Group-based inpatient programme or inpatient programme together with a home-based programme.	Multidisciplinary rehabilitation programme incorporating medical input, psychology and physiotherapy or psychology-based education, exercise, peer support group activity and medical input. Duration: 3 to 10 weeks of 3 sessions per week.	Local QoL measure, EORTC QLQ-C30	There was 'low level' evidence that multidisciplinary rehabilitation can improve QoL over 12 months. Not possible to suggest optimal frequency, or one type of intervention over another.

For peer review only

McAlpine et al, 2015	To examine the evidence-based literature surrounding the use of online resources for adult cancer patients.	14 studies/ 9 RCTs	2,351 lung, prostate, breast, head and neck and mixed cancer patients. The sample size for the RCTs was 1,121 patients and their mean age ranged from 49.5 - 67.2 years.	Survivors are defined as patients who have had a cancer diagnosis in the past, including those currently receiving active treatment, those in remission or cured and those who are in the terminal stages of disease.	A variety of online platforms were used including email, online educational resources, online support groups or message boards, cancer information websites and interactive websites.	Three interventions: (i) Linking patients to their treating team of clinicians<br>(ii) Connecting patients with each other<br>(iii) Educational resources.<br><br>Duration: 4 weeks to 12 months.	FACT-B, SF-12, EORTC QLQ-C30, EQ-5D, EPIC-26, 15DHRQoL, bespoke QoL measure	The overall benefit of online interventions for cancer patients is unclear. Although there is significant promise, the few interventions that have been rigorously analysed demonstrate mixed efficacy, often of limited duration.
Mewes et al, 2012	To systematically review the evidence on the effectiveness of multidimensional rehabilitation programs for cancer survivors and to critically review the cost-effectiveness studies of cancer rehabilitation.	16 studies originated from 11 trials (11 RCTs, 3 pre-test-post-test, 1 quasi experimental, 1 longitudinal)	2,175 mixed cancer patients, predominantly breast. RCTs included from 21 to 199 participants. Age range not specified.	Patients with any type of cancer who finished primary treatment with an expected survival duration of at least 1 year. Hormone therapy could still be ongoing.	Face to face in an inpatient setting	Multidimensional rehabilitation defined as consisting of two or more rehabilitation interventions directed at the ICF dimensions. Interventions typically included exercise, CBT, psychotherapy, education and return to work interventions.<br>Programme duration: 4 to 15 weeks.	EORTC QLQ-C30, RAND-36, FACT-G, FACT-B, SF-12	Effect sizes for QoL were in the range of -0.12 (95% CI:-0.45 to -0.20) to 0.98 (95% CI, 0.69 to 1.29).<br><br>Multi and mono dimension interventions were equally effective.

For peer review only - http://bmjopen.bmj.com/site/about/guidelines.xhtml

Mishra et al, 2012	To evaluate the effectiveness of exercise on overall HRQoL and HRQoL domains among adult post-treatment cancer survivors.	40 trials / 38 RCTs	3,694 mixed cancer patients during and post-treatment were randomised. Over 50% included breast cancer patients only. Mean age range: 39 to 68 years	Participants who have completed treatment	Settings included a gym, community centre, yoga studio, or university or hospital facility. Home-based interventions were included.	Exercise was defined as physical activity causing an increase in energy expenditure in a systematic manner in terms of frequency, intensity, and duration. Included prescribed, active exercise formats of aerobic, resistance , stretching or aerobic/resistance combinations. Some interventions included modules in psychological or behavioural education. Duration ranged from 3 weeks to one year. Frequency varied between daily to once per week. Sessions lasted from 20 to more than 90 minutes.	EORTC QLQ-C30, FACT-G, FACT-B, FACT-F, FACT-An, FACT-Lym, FACIT-F, CARES-SF, QoL Index, SF-36, Neck Dissection Impairment Index for QoL for head and neck cancer survivors.	Exercise has a positive impact on QoL with improvements in global QoL.
Osborn et al, 2006	To investigate the effects of CBT and patient education (PE) on commonly reported problems (i.e. depression, anxiety, pain, physical functioning and quality of life) in adult cancer survivors.	15 RCTs	1,492 mixed cancer patients. Age range: 18-84 years.	Defined as beyond the time of diagnosis	In a group or individually, face to face	Interventions included; Group or Individual CBT, Patient Education. CBT intervention duration ranged from 3 – 55 weeks. Frequency varied from 1 hour per week to 2 hours per week. PE duration ranged from one 20-minute session to 6 weekly one-hour sessions.	FACT	QoL was improved at short-term and long - term follow up after CBT. Patient Education was not related to improved outcomes. Individual interventions were more effective than group.

For peer review only - http://bmjopen.bmj.com/site/about/guidelines.xhtml

Smits et al, 2015	To evaluate the effectiveness of lifestyle intervention in improving QoL of endometrial and ovarian cancer survivors.	8 studies / 3 RCTs	413 survivors of endometrial and ovarian cancer were included in the analysis. 153 survivors were included in the RCTs. Age range not specified.	Adults diagnosed with endometrial cancer having completed primary treatment (surgery, chemotherapy or radiotherapy)	Home-based, individually or group-based	Physical activity, behavioural change, nutritional, counselling interventions<br>The duration varied from 12 weeks to 12 months.	FACT-G, FACT-F, FACT-O, SF-36 and QLACS	The review did not show improvements in global QoL. The authors concluded that lifestyle interventions have the potential to improve QoL in this population.
Spark et al, 2013	To determine the proportion of physical activity and/or dietary intervention trials in breast cancer survivors that assessed post-intervention maintenance of outcomes, the proportion of trials that achieved successful post-intervention maintenance of outcomes, and the sample,	16 studies originated from 10 RCTs	1,536 breast cancer survivors during or after treatment. Age range not specified.	Not specified	Interventions included face to face contact, printed information and telephone counselling or home-based delivery	Interventions were described as physical activity and/or dietary behaviour change aiming to increase aerobic fitness, strength, physical activity.<br>Most interventions lasted 1-4 months, with some lasting longer than 6 months.	Measures not specified	More research is needed to identify the best ways of supporting survivors to make and maintain these lifestyle changes. QoL-specific outcomes from three studies not reported.

For peer review only - http://bmjopen.bmj.com/site/about/guidelines.xhtml

	intervention, and methodological characteristics common among trials that achieved successful post intervention maintenance of outcomes.							
Spence et al, 2009	To summarise the literature on the health effects of exercise during cancer rehabilitation and to evaluate the methodological rigor of studies in this area.	13 studies originated from 10 trials, 4 of which were RCTs	327 mixed cancer patients, mostly breast cancer patients. The sample size for the RCTs was 245 patients and their mean age ranged from 18 to 65 years.	Patients who had recently completed treatment and had reported no plans for additional treatment. 'Recently completed' was defined as having completed treatment no more than 12 months prior to enrolment.	Interventions were either supervised exercise programmes or home-based, unsupervised exercise programmes. One study employed exercise physiologists to prescribe individually-tailored exercise programmes.	Most interventions were aerobic or resistance-training exercise programmes. Most studies prescribed cycling or walking ergometers for the aerobic component. Studies incorporating resistance training prescribed either exercises using machines or resistance bands. Duration varied from 2 weeks to 14 weeks with a frequency of daily exercise to two or three sessions per week.	Cancer Rehabilitation Evaluation System	The findings from this review suggest that exercise can provide a variety of benefits for cancer survivors during the rehabilitation period, including an improved QoL.

For peer review only

For peer review only - http://bmjopen.bmj.com/site/about/guidelines.xhtml

Zachariae et al, 2015	To evaluate the effectiveness of expressive writing for improving psychological and physical health in cancer patients and survivors.	16 RCTs	1,797 cancer patients or survivors. Breast cancer, ovarian, renal, prostate, colorectal and mixed cancers. Age range not specified.	Not specified	Lab or home-based	Expressive writing interventions requiring participants to disclose their emotions in sessions. The duration of the intervention ranged from 3 – 4 sessions, which were daily, weekly or bi-weekly.	FACT-B, FACT-G, FACT-BMT, QLQ-C30	The review did not support the general effectiveness of expressive writing in cancer patients and survivors.
Zeng et al, 2014	To examine the effectiveness of exercise intervention on the quality of life of breast cancer survivors.	25 studies included in the qualitative synthesis, 19 studies included in meta-analysis. 22 RCTs	1,073 breast cancer patients aged 18 years or over.	Individuals who had completed active cancer treatment.	Face to face, by telephone	Interventions included any type of exercise - aerobic, resistance or combination of aerobic and resistance, yoga, tai chi, aerobic and strength training, aerobic and resistance training and stretching. The duration of the intervention ranged from 4 to 52 weeks. Time per session varied from 15 to 90 mins, 1 to 5 times per week.	Generic QoL measures: SF-36, FACT-G, EORTC-QLQ-C30. Cancer site-specific QoL measures: FACT-B, EORTC QLQ BR23.	The review found consistent positive effects of exercise interventions in overall QoL and certain QoL domains. There was a small to moderate effect of interventions on site-specific QoL. Single type of exercise intervention general aerobic, yoga or tai chi had significant differences in QoL score changes.

For peer review only

Abbreviations: QoL = Quality of Life; HRQoL = Health-related Quality of Life; 15DHRQoL = 15-dimensional health related quality of life; ABS = Affects balance scale; CARES = Cancer rehabilitation evaluation system; CARES-SF = Cancer Rehabilitation Evaluation System Short Form; EORTC QLQ-C30 = European Organisation for Research and Treatment of Cancer Quality of Life Questionnaire C30; EORTC QLQ-C33 = European Organization for Research and Treatment of Cancer Quality of Life Questionnaire C33; EPIC = Expanded Prostate Cancer Index Composite; EPIC-26 = Expanded Prostate Cancer Index Composite Short Form; EQ-5D, EuroQol-5D ''feeling thermometer''; FACIT-F = Functional Assessment of Chronic Illness—Fatigue; FACIT-Sp = Functional Assessment of Chronic Illness—Spiritual well-being; FACT-An = Functional Assessment of Cancer Therapy—Anaemia scale; FACT-B = Functional Assessment of Cancer Therapy—Breast Cancer; FACT-C = Functional Assessment of Cancer Therapy—Colorectal; FACT-F = Functional Assessment of Cancer Therapy—Fatigue; FACT-G: Functional Assessment of Cancer Therapy—General; FACT H&N = Functional Assessment of Cancer Therapy—Head & Neck; FACTIT = Functional Assessment of Chronic Illness Therapy; FACT-Lym = Functional Assessment of Cancer Therapy—Lymphoma; FACT-O = Functional Assessment of Cancer Therapy—Ovarian; FACT-P = Functional Assessment of Cancer Therapy—Prostate; FLIC= Functional Living Index for Cancer; GHQ = General health questionnaire; IFS-CA = Inventory of functional status—cancer; MCS/PCS = Mental Component Score/Physical Component Score; Modified Rotterdam QoL Survey; Neck Dissection Impairment Index for QoL for head and neck cancer survivors; NHP = Nottingham Health Profile; PCa-QoL = Prostate Cancer Quality of Life Instrument; QLACS = Quality of Life in Adult Cancer Survivors; QLI = Quality of life index; QLQ-PR25 = European Organisation for Research and Treatment of Cancer Quality of Life Questionnaire-Prostate Module; QoL-BC = quality of life questionnaire—breast cancer; RAND-36 = 36-Item Short Form Health Survey; SDS = Symptom Distress Scale; SF-12 = Medical Outcomes Study Short-Form Health Survey 12; SF-36 = Medical Outcomes Study Short-Form Health Survey 36; SIP = Sickness impact profile; UCLA-PCI = University of California, Los Angeles, Prostate Cancer Index; VAS = Visual analogue scale; WHOQOL-BREF = World Health Organization Quality of Life Assessment.

For peer review only - http://bmjopen.bmj.com/site/about/guidelines.xhtml

Table 4: Components of the interventions by study

	Cramer et al, 2012[23]	Fong et al, 2012[10]	Buffart et al, 2012[11]	Khan et al, 2012[8]	Mishra et al, 2012[12]	Culos-Reed et al, 2012[14]	Bourke et al, 2015[28]	Duijts et al, 2011[9]	Ferrer et al, 2011[19]	Fors et al, 2011[24]	Galveo et al, 2005[13]	Gerritsen and Vincent 2015[20]	Huang et al, 2015[27]	McAlpine et al, 2015[15]	Mewes et al, 2012[18]	Osborn et al, 2006[17]	Smits et al, 2015[21]	Spark et al, 2013[25]	Spence et al, 2009[16]	Zachariae et al, 2015[22]	Zeng et al. 2014[26]
**PHYSICAL**																					
Aerobic		•			•		•	•	•		•						•		•		•
Aerobic and Resistance					•		•														•
Resistance					•		•				•										•
Aquatic exercise					•																
Cardiovascular programme											•										•
Cycling					•						•	•							•		
Dance movement								•													
Enhanced Standard Care							•														
Exercise not specified				•											•		•	•			
Expressive writing																				•	
METs targeted									•												
Dietary intervention				•			•										•	•			
Pilates					•																
Resistance/strength training		•			•			•			•	•					•		•		•
Running					•																
Self-management exercise								•													
Stretching/Flexibility exercises											•								•		•
Swimming												•									
Tai Chi					•																•
Treadmill																			•		
Walking					•			•			•	•							•		
Weight training								•													
Yoga/meditation	•		•		•	•		•													•
Qigong					•																
**PSYCHOLOGICAL, EDUCATIONAL & BEHAVIOURAL**																					
Body mind								•													
Cognitive behavioural							•	•													

For peer review only

For peer review only - http://bmjopen.bmj.com/site/about/guidelines.xhtml

	Cramer et al, 2012[23]	Fong et al, 2012[10]	Buffart et al, 2012[11]	Khan et al, 2012[8]	Mishra et al, 2012[12]	Culos-Reed et al, 2012[14]	Bourke et al, 2015[28]	Duijts et al, 2011[9]	Ferrer et al, 2011[19]	Fors et al, 2011[24]	Galveo et al, 2005[13]	Gerritsen and Vincent 2015[20]	Huang et al, 2015[27]	McAlpine et al, 2015[15]	Mewes et al, 2012[18]	Osborn et al, 2006[17]	Smits et al, 2015[21]	Spark et al, 2013[25]	Spence et al, 2009[16]	Zachariae et al, 2015[22]	Zeng et al. 2014[26]
stress therapy																					
Cognitive behavioural therapy								●		●					●	●	●				
Cognitive G therapy								●													
Combined psychosexual								●													
Comprehensive coping strategy								●													
Coping skills																					
Emotional support								●		●											
Group therapy							●	●								●	●				
Guided imagery								●													
Image consultant				●																	
Mindfulness based stress reduction programme													●								
Motivational interviewing																					
Problem solving training								●													
Progressive relaxation training																					
Psychotherapy															●						
Psychosocial therapy								●													
Return to work interventions															●						
Social support								●		●											
Stress management								●													
Health education								●							●	●					
Psychological education				●			●	●		●					●	●					
Peer support				●				●						●							
**MODE OF DELIVERY**																					
CD/manuals/videos			●				●			●											
Face to face				●	●		●	●									●	●			
Home based			●	●	●			●									●	●	●		
Inpatient setting				●											●						
Multidisciplinary rehabilitation programme				●			●								●						

For peer review only - http://bmjopen.bmj.com/site/about/guidelines.xhtml

	Cramer et al, 2012[23]	Fong et al, 2012[10]	Buffart et al, 2012[11]	Khan et al, 2012[8]	Mishra et al, 2012[12]	Culos-Reed et al, 2012[14]	Bourke et al, 2015[28]	Duijts et al, 2011[9]	Ferrer et al, 2011[19]	Fors et al, 2011[24]	Galveo et al, 2005[13]	Gerritsen and Vincent 2015[20]	Huang et al, 2015[27]	McAlpine et al, 2015[15]	Mewes et al, 2012[18]	Osborn et al, 2006[17]	Smits et al, 2015[21]	Spark et al, 2013[25]	Spence et al, 2009[16]	Zachariae et al, 2015[22]	Zeng et al. 2014[26]
Printed information			•															•			
Support from nurse or voluntary organisations							•	•		•											
Telephone	•		•					•										•			•
Web based							•	•		•				•							

For peer review only

For peer review only - http://bmjopen.bmj.com/site/about/guidelines.xhtml

Table 5: Reported effect size from meta-analyses in reviews

Authors	Intervention	Type of effect size reported	Reported effect size	Overall finding
Buffart et al, 2012◊	Yoga	SMD (7 studies)		
		General QoL	0.37,0.11-0.62	+
Cramer et al, 2012◊	Yoga	SMD (4 studies) Global QoL	0.62, 0.04 to 1.21;	+
Ferrer et al, 2011 * $	Exercise	SMD (78 studies)		
		All intervention groups (Immediate FU)	0.34, 0.24 to 0.43	+
		Intervention vs control, adjusted for baseline differences	0.24, 0.12 to 0.35	+
		Delayed FU		
		All intervention groups	0.42, 0.23 to 0.61	+
		Intervention vs Control adjusted for baseline	0.20, -0.058 to 0.46	+
Fong et al, 2012	Exercise	2 studies	3.4, 0.4 to 6.4	+
		9 studies	22.1, 16.8 to 27.4	+
Gerritsen and Vincent 2015	Exercise	SMD: intervention vs control	5.55, 3.19 to 7.9	+
Mishra et al, 2012 ◊	Exercise	SMD: baseline to after intervention (11 studies)	0.48, 0.16 to 0.81	+
		3-6 month follow up (181 participants)	0.14, -0.38 to 0.66	-
		6 month follow up (115 participants) (2 studies)	0.46, 0.09 to 0.84	+
Zeng et al, 2014	Exercise	Standardised Mean Difference (0verall) (6 studies)	0.70, 0.21, 1.19	+
		Cancer specific (10 studies)	0.38, 0.03 to 0.74	+
Duijts et al, 2011	Exercise	SMD (or Hedges g for small sample size, with adjustment) (27 studies)	0.298, 0.117 to 0.479, p = 0.001	+

For peer review only - http://bmjopen.bmj.com/site/about/guidelines.xhtml

	Behavioural intervention		0.045, -0.044 to 0.135, p=0.322	uncertain
Osborn et al, 2006	CBT	SMD Overall (11 studies)	0.91, 0.38 to 1.44, p<0.01	+
		Short term (<8wks)	1.45, 0.43 to 2.47	+
		Long term (>8wks)	0.26, 0.06 to 0.46	+
		Individual CBT (7 studies)	0.95, -0.367 to 1.536	+
		Individual vs Group CBT (1 study)	0.37, -0.02 to 0.75,	uncertain
	Patient Education	(1 study)	-0.04, -0.38 to 0.29,	-
Smits et al, 2015	Lifestyle interventions	SMD		+
		3 months	1.16, -5.91 to 8.23,	
		6 months	2.48, -4.63 to 9.58,	
Zachariae et al, 2015	Expressive writing	Hedges's *g*	0.09, -0.5 to 0.24,	+

* random effects assumption
◊ Reviews rated as high quality
$ findings sustained for random or fixed effects, random effects reported.

For peer review only

Overall Effectiveness of Interventions: meta-analysis findings

Meta-analyses were reported in 11 reviews and the effect sizes (as reported in the original reviews) are tabulated (Table 5). Of six publications providing meta-analyses of physical activity (not including yoga), all found convincing positive associations for studies testing response between 1 and 26 weeks post-treatment. Long term effects were not tested by all, although Fong and Zeng did show persistent effects at six months and a year respectively. [10 26] One review [19] showed uncertain outcomes at 3-6 months, although shorter and longer term outcomes were favourable. This review showed equivocal effects when the intervention group was compared with the control group, once adjusted for quality of life and covariates at baseline. The two meta-analyses of yoga interventions showed positive effects, [11 23] as did a review of CBT. [17] There was no evidence of benefit in quality of life following patient education [17] and behavioural interventions.[9]

For peer review only - http://bmjopen.bmj.com/site/about/guidelines.xhtml

Two reviews reported effect sizes from individual studies but did not undertake meta-analyses.[18 24] Mewes's review of multidimensional rehabilitation included ten studies, nine of which had global quality of life outcomes; of these, seven showed benefit with effect sizes ranging from 0.04 to 0.99 (no confidence intervals reported). [18] Fors's review included six RCTs only four of which included a quality of life measure; [24] two of these showed positive effect sizes (ranging from 0.56, 95%CI: 0.09 to 1.03; 95% CI: 0.63, 0.11 to 1.18); one showed improved and one a worsening of quality of life as a non-standardised mean score. Five reviews [8 13 15 16 25] did not report meta-analyses or effect sizes; mostly these provided mean change scores or narrative statements. On the whole these gave a mixed picture, often resorting to sub-group analysis by cancer type or different dimensions of quality of life.

Physical Activity: Summary Findings

Cramer's[23] high quality review of 6-12 weeks of yoga in breast cancer patients showed a large increase in general quality of life, a finding that was consistent with reviews by Buffart [11] and Culos-Reed, which scored lower on the AMSTAR. [14] Mishra's [12] high quality review of people with multiple cancers, 50% of whom had breast cancer, found that physical activity had a positive effect on global quality of life at three and six months follow up, as did Smits's high quality review of endometrial cancer and Gerritsen's moderate quality review of mixed cancers.[20 21] Fong's[10] high quality review of breast cancer, colorectal, endometrial and mixed cancers, similarly found physical interventions improved general quality of life on average at 13 weeks follow up (range 3-60 weeks). Bourke's review of prostate cancer found personalised lifestyle interventions helpful[28], and McAlpine's review of mixed cancers including prostate found benefit of activity following medication treatment.[15]

There was inconsistency across the reviews with regard to the types of exercise interventions that were most effective. Fong [10] found aerobic plus resistance training to be significantly more effective

For peer review only - http://bmjopen.bmj.com/site/about/guidelines.xhtml

than aerobic training alone on many aspects of quality of life. However, Zeng's [26] moderate quality review suggested that single types of exercise interventions (general aerobic, yoga or tai chi) were more effective at increasing quality of life at 4-52 weeks after intervention; half of the studies assessed interventions between 8-12 weeks. Duijts's [9] study of breast cancer patients found only small effects of physical activity on quality of life (at 8 -26 weeks after intervention); and Spence's [16] study of mixed but mostly breast cancer patients reported evidence that physical activity improved overall quality of life but only four of ten trials maintained the intervention and only a fifth of trials seemed to assess outcome at 3 months and beyond. Zeng's [26] review of breast cancer patients found small but positive benefits of physical activity on overall quality of life. Galvão's [13] review of mixed cancers gave preliminary evidence of positive benefits on a Modified Rotterdam QoL measure, but no overall effects were reported. However, Spark's [25] review of breast cancer patients showed that the impact of physical activity on quality of life was not convincing. Although Spark did not report effect sizes, two of the studies in that review included quality of life measures, both of which reported effect sizes in the original papers: one showed positive benefits on FACT-G and FACT-B at 8 months (effect sizes 9.8 to 13.4), but not at 24 months follow up; the other showed no significant effects on FACT-G overall, but when the cancer specific FACT-G was assessed at six month follow up, there was benefit (4.9, 0.2 to 9.6). Ferrer's [19] study of breast, prostate, endometrial, head and neck, ovarian cancers and lymphoma found small but positive effects of exercise at long term follow up on multiple measures of quality of life. The efficacy of the interventions appeared greater with shorter duration treatments, and if exercise was supervised. Aerobic intensity predicted improvements in quality of life.

Psychological and behavioural interventions: Summary Findings

Only one of the reviews of psychological and behavioural interventions was classified as high quality: Huang's [27] meta-analysis of breast cancer patients showed that mindfulness-based stress reduction

For peer review only - http://bmjopen.bmj.com/site/about/guidelines.xhtml

programmes had a significant effect in improving overall quality of life. Duijts's [9] review, on the other hand, concluded that behavioural techniques such as problem solving, stress management and CBT did not significantly improve health-related quality of life. Nevertheless, Fors's [24] review of breast cancer patients showed CBT improved quality of life. No meta-analysis or overall effect sizes were reported due to heterogeneity. Further support for CBT came from Osborn's [17] review of group and individually delivered CBT for mixed cancers; individual interventions were more effective than group-based treatment. CBT showed both short-term [24] and long-term improvements in quality of life.[17] Five primary papers in one review assessed the effect of social and emotional support as an intervention, four of them finding no effect, and one reporting a significant improvement in quality of life on one measure.[24] There was no evidence that psychosocial education increased quality of life.[17 24]

Multidimensional and Multidisciplinary rehabilitation

Khan's [8] high quality review of breast cancer patients included just two studies, only one of which provided low level evidence that multidisciplinary rehabilitation improved participation and social activities. The other showed no significant effects. Mewes's [18] moderate quality review of breast and other cancers treated by inpatient multidisciplinary rehabilitation demonstrated no differences between multidimensional and single dimension interventions, with benefits of both on physical outcomes. Bourke's review of prostate cancer survivors examined the effectiveness of multidisciplinary approaches based on findings from three primary studies.[28] They concluded that such interventions showed small benefits for quality of life, typically when they involved a smaller number of health professionals, thus allowing more focused tailoring of the interventions.

For peer review only - http://bmjopen.bmj.com/site/about/guidelines.xhtml

Intervention modality

The effectiveness of online educational interventions was unclear. McAlpine's [15] review of lung, prostate, head and neck and a smaller number of mixed cancers showed equivocal findings. There were benefits to online education, message boards, but mixed effects for interactive websites, and worse outcomes from one study on email interventions. One interesting review was of expressive writing interventions, but this found no benefit on quality of life, although small effects would be undetected. [22] Individuals with low levels of emotional support appeared to benefit more than others.

Adverse Effects

Five reviews[11 12 15 23 26] included reports of adverse events. Of four studies in Buffart's[11] review, one reported back spasm in a yoga class in a patient with a history of back problems. In Cramer's[23] review of three studies reporting adverse events, there was one adverse event (back spasm) in 138 patients. McAlpine's[15] review included two studies that reported adverse effects of online support groups. One of these reported transient helplessness, anxiety, confusion and depression at six months; whilst the other showed poorer quality of life despite high levels of reported satisfaction. Zeng's[26] review of 25 trials found one study with reports of exercise related lymphedema. In Mishra's[12] review, six studies reported adverse effects including lymphoedema, gynaecological complications and influenza in the exercise group. One study reported back, knee and hip problems. Three participants in one study reported thrombosis and infection following exercise interventions. Another study found hip pain, sciatica, arm discomfort (n = 4), knee discomfort (n = 10), ankle discomfort (n = 3), and foot discomfort (n = 8) with asymptomatic ischaemia and conduction problems on ECG. A further study reported lung metastases, pulmonary embolism and palpitations. Another study reported soft tissue injury following exercise, and cholecystitis following stroke.

For peer review only - http://bmjopen.bmj.com/site/about/guidelines.xhtml

Cancer recurrence, although not a direct effect of interventions, was common and another reason to stop participation in the research.

DISCUSSION

Main findings

21 reviews were included and showed a lack of definitive and consistent evidence across 465 primary studies of which 362 were RCTs. In part this is explained by substantial variation in study designs and outcome measures used to indicate quality of life. All systematic reviews of physical activity demonstrated improved overall quality of life, but few studies assessed long-term outcomes beyond 3 months, and even fewer assessed outcomes beyond a year after the intervention. More focused research and a consistent approach are required to explore the effect on the subdomains of quality of life.[12] A higher quality review suggests that aerobic plus resistance training provide maximum improvements in quality of life.[10] There was more evidence of physical rather than psychological or other types of interventions.

One of the included reviews for psychological or behavioural interventions was of high quality.[27] CBT is effective for improving quality of life in the short and long term,[17 24] especially when provided as an individual intervention.[17] There is not much evidence to support comparative effectiveness of intervention modalities such as group versus individual, mono-dimensional versus multi-dimensional or multidisciplinary; further work is needed to examine these different approaches. Given the accessibility of social media and its popularity, the findings that email contact was related to poorer quality of life need further investigation; although interactive websites were beneficial, the overall findings about digital interventions were equivocal.

For peer review only - http://bmjopen.bmj.com/site/about/guidelines.xhtml

Limitations

The current review has some limitations in the methodology. Studies not in English and grey literature were not included due to time constraints as the review was undertaken as a part of a programme development grant to inform the design of a future research programme application.

We encountered some methodological limitations in included reviews. Some used multiple outcomes and often had a very broad understanding of QoL and used diverse measures of quality of life. There was no consistent reporting standard.

We did not consider outcomes such as wellbeing or the multiple sub-domains of quality of life to avoid the risk of generating findings due to multiple testing in smaller sub-samples in underpowered anlayses. Some reviews included few primary papers.

We found little overlap between reviews (tabulation available on request), reflecting their specific inclusion and exclusion criteria and interest in very specific interventions and cancer types. We did not evaluate the methodological quality or bias of the original studies within each systematic review. Ten reviews planned to assess publication bias; three of these could not perform any specific tests of bias due to small samples.[8 23 27] Consequently seven studies tested for publication bias.[9 10 12 17 19 20 22] Three of these reported that publication bias was not significant.[10 20 22] Four reviews [9 12 17 19] reported significant publication bias suggesting caution in assuming there is definitive evidence for exercise and CBT.

The physical and psychosocial concerns of patients at different time periods of the cancer experience will vary greatly and interventions effective at one stage may not be suitable for another. Most reviews defined 'survivors' as those who had completed active treatment before the onset of the study. [10 13 14 16 18 19 23 24 26] Some specified a time frame, from immediately after surgery to 15 years after active treatment. [12] One review defined survival as being from diagnosis onwards. [17] Another

For peer review only - http://bmjopen.bmj.com/site/about/guidelines.xhtml

included terminal stages of cancer. [15] The majority of the reviews incorporated studies combining patients during and post treatment. [9 11-15 23-25] These differing definitions of living with and beyond cancer make comparison difficult, and a standardised approach to trials and reporting of studies is needed.

Interventions were offered to patients based on their diagnosis of cancer, rather than low quality of life, which may have led to underestimation of potential beneficial effects. Future research should consider the effectiveness of interventions targeting people living beyond all types of cancer, and with poor overall quality of life.

Conclusions

Systematic reviews of cancer patients and their QoL showed that effective interventions included physical activity, CBT and mindfulness-based stress reduction training. Personalised lifestyle interventions showed promise, as did social and emotional support. Educational and information provision appear ineffective, and there were few studies of electronic interventions. Currently, there is no standard study design, outcome selection, or reporting convention adopted across these reviews. No single intervention can be recommended to those patients with a poor quality of life following cancer treatment as interventions were not targeting poorer quality of life, but cancer survivors in general.

For peer review only - http://bmjopen.bmj.com/site/about/guidelines.xhtml

Acknowledgements, competing interests, funding and all other required statements

This review was funded by NIHR-Programme Development Grant: RP-DG-1212-10014

The authors declare that they do not have any conflicts of interest.

The lead and corresponding authors (guarantors) affirm that the manuscript is an honest, accurate, and transparent account of the study being reported; that no important aspects of the study have been omitted; and that any discrepancies from the study as planned (and, if relevant, registered) have been explained. All authors had access to the full data set, and the work was undertaken independently of the funders and sponsor.

Contributor Statement:

KB as PI for the review, designed the review, and prepared the review section for the original grant application, which overall was led by PDW. Input on design was provided by all authors (KB, MD, JD, RR, LJ, LB, AM, TC, MT, ST, AK, PW) and PPI experts (Miriam Harris, Adrienne Morgan, and Louisa Smalley) in steering groups during preparation of the funding application and throughout the project; more specific additional input to design was provided by PW, SC. MD and JD were research fellows employed on the grant, and collected the papers, ran the searches and performed the first extraction under supervision by KB. MD and JD undertook the preliminary charting and extraction. EH (a PPI expert) and EM conducted the AMSTAR ratings, the final data extraction and edited the draft, under the supervision of KB. KB reviewed all data and checked and completed extraction of the data and identified relevant effect estimates, and led on writing the paper, edited consecutive drafts of the MS, and the produced the final draft. All authors (KB, EM, EH, MD, JD, RR, LJ, LB, AM, TC, MT, ST, AK, PW) contributed to the reviewing consecutive drafts of the paper for content, the presentation, and discussion about the findings and interpretation at each stage of the review process, as well as the structure of the paper. All authors (KB, EM, EH, MD, JD, RR, LJ, LB, AM, TC, MT, ST, AK, PW) commented on and approved the final version. We thank Miriam Harris, Adrienne Morgan, and Louisa Smalley for helpful analysis and comments in the design, planning and delivery of the research including this review, and in the construction of SURECAN dissemination plans and the design of a future trial.

The Corresponding Author has the right to grant on behalf of all authors and does grant, on behalf of all authors, a worldwide licence to the Publishers and its licensees in perpetuity, in all forms, formats and media (whether known now or created in the future), to i) publish, reproduce, distribute, display and store the Contribution, ii) translate the Contribution into other languages, create adaptations, reprints, include within collections and create summaries, extracts and/or, abstracts of the Contribution, iii) create any other derivative work(s) based on the Contribution, iv) to exploit all subsidiary rights in the Contribution, v) the inclusion of electronic links from the Contribution to third party material where-ever it may be located; and, vi) licence any third party to do any or all of the above.

Data Sharing Statement

No additional data are available.

For peer review only - http://bmjopen.bmj.com/site/about/guidelines.xhtml

Annex1: Full search strategy (supplementary file)

For peer review only

For peer review only - http://bmjopen.bmj.com/site/about/guidelines.xhtml

REFERENCES

1. Cancer Research UK C. Cancer Survival Statistics 2015 [Available from: http://www.cancerresearchuk.org/health-professional/cancer-statistics/survival.
2. Jarrett N, Scott I, Addington-Hall J, et al. Informing future research priorities into the psychological and social problems faced by cancer survivors: A rapid review and synthesis of the literature. *European Journal of Oncology Nursing* 2013;17(5):510-20. doi: http://dx.doi.org/10.1016/j.ejon.2013.03.003
3. Miller KD, Triano LR. Medical issues in cancer survivors--a review. *Cancer J* 2008;14(6):375-87. doi: 10.1097/PPO.0b013e31818ee3dc [published Online First: 2008/12/09]
4. MacmillanCancerSupport. Worried Sick: the emotional impact of cancer http://www.macmillan.org.uk/documents/getinvolved/campaigns/campaigns/impact_of_cancer_english.pdf: Macmillan Cancer Support 89 Albert Embankment London SE1 7UQ; 2006 [
5. NHS Choices. Complementary and alternative medicine [Available from: http://www.nhs.uk/Livewell/complementary-alternative-medicine/Pages/complementary-and-alternative-medicine.aspx accessed October 2016.
6. Shea BJ, Grimshaw JM, Wells GA, et al. Development of AMSTAR: a measurement tool to assess the methodological quality of systematic reviews. *BMC Med Res Methodol* 2007;7:10. doi: 10.1186/1471-2288-7-10 [published Online First: 2007/02/17]
7. Mays N, Pope C, Popay J. Systematically reviewing qualitative and quantitative evidence to inform management and policy-making in the health field. *Journal of health services research & policy* 2005;10 Suppl 1:6-20. doi: 10.1258/1355819054308576 [published Online First: 2005/08/02]
8. Khan F, Amatya B, Ng L, et al. Multidisciplinary rehabilitation for follow-up of women treated for breast cancer. *Cochrane Database Syst Rev* 2012;12:Cd009553. doi: 10.1002/14651858.CD009553.pub2 [published Online First: 2012/12/14]
9. Duijts SF, Faber MM, Oldenburg HS, et al. Effectiveness of behavioral techniques and physical exercise on psychosocial functioning and health-related quality of life in breast cancer patients and survivors--a meta-analysis. *Psychooncology* 2011;20(2):115-26. doi: 10.1002/pon.1728 [published Online First: 2010/03/26]
10. Fong DYT, Ho JWC, Hui BPH, et al. Physical activity for cancer survivors: meta-analysis of randomised controlled trials. *BMJ (Clinical research ed)* 2012;344 doi: 10.1136/bmj.e70
11. Buffart LM, van Uffelen JG, Riphagen, II, et al. Physical and psychosocial benefits of yoga in cancer patients and survivors, a systematic review and meta-analysis of randomized controlled trials. *BMC Cancer* 2012;12:559. doi: 10.1186/1471-2407-12-559 [published Online First: 2012/11/28]
12. Mishra SI, Scherer RW, Geigle PM, et al. Exercise interventions on health-related quality of life for cancer survivors. *Cochrane Database of Systematic Reviews* 2012(8) doi: 10.1002/14651858.CD007566.pub2
13. Galvão DA, Newton RU. Review of exercise intervention studies in cancer patients. *Journal of clinical oncology : official journal of the American Society of Clinical Oncology* 2005;23(4):899-909. doi: 10.1200/jco.2005.06.085 [published Online First: 2005/02/01]
14. Culos-Reed SN, Mackenzie MJ, Sohl SJ, et al. Yoga & cancer interventions: a review of the clinical significance of patient reported outcomes for cancer survivors. *Evidence-based complementary and alternative medicine : eCAM* 2012;2012:642576. doi: 10.1155/2012/642576 [published Online First: 2012/11/06]
15. McAlpine H, Joubert L, Martin-Sanchez F, et al. A systematic review of types and efficacy of online interventions for cancer patients. *Patient Education and Counseling* 2015;98(3):283-95.

For peer review only - http://bmjopen.bmj.com/site/about/guidelines.xhtml

16. Spence RR, Heesch KC, Brown WJ. Exercise and cancer rehabilitation: a systematic review. *Cancer Treat Rev* 2010;36(2):185-94. doi: 10.1016/j.ctrv.2009.11.003 [published Online First: 2009/12/08]
17. Osborn RL, Demoncada AC, Feuerstein M. Psychosocial interventions for depression, anxiety, and quality of life in cancer survivors: meta-analyses. *Int J Psychiatry Med* 2006;36(1):13-34. [published Online First: 2006/08/25]
18. Mewes JC, Steuten LMG, M.J IJ, et al. Effectiveness of multidimensional cancer survivor rehabilitation and cost-effectiveness of cancer rehabilitation in general: A systematic review. *Oncologist* 2012;17(12):1581-93.
19. Ferrer RA, Huedo-Medina TB, Johnson BT, et al. Exercise interventions for cancer survivors: a meta-analysis of quality of life outcomes. *Annals of behavioral medicine : a publication of the Society of Behavioral Medicine* 2011;41(1):32-47. doi: 10.1007/s12160-010-9225-1 [published Online First: 2010/10/12]
20. Gerritsen JK, Vincent AJ. Exercise improves quality of life in patients with cancer: a systematic review and meta-analysis of randomised controlled trials. *British journal of sports medicine* 2016;50(13):796-803. doi: 10.1136/bjsports-2015-094787 [published Online First: 2016/01/01]
21. Smits A, Lopes A, Das N, et al. The effect of lifestyle interventions on the quality of life of gynaecological cancer survivors: A systematic review and meta-analysis. *Gynecol Oncol* 2015;139(3):546-52. doi: 10.1016/j.ygyno.2015.10.002 [published Online First: 2015/10/07]
22. Zachariae R, O'Toole MS. The effect of expressive writing intervention on psychological and physical health outcomes in cancer patients—a systematic review and meta-analysis. *Psycho-Oncology* 2015;24(11):1349-59. doi: 10.1002/pon.3802
23. Cramer H, Lange S, Klose P, et al. Yoga for breast cancer patients and survivors: a systematic review and meta-analysis. *BMC Cancer* 2012;12:412. doi: 10.1186/1471-2407-12-412 [published Online First: 2012/09/20]
24. Fors EA, Bertheussen GF, Thune I, et al. Psychosocial interventions as part of breast cancer rehabilitation programs? Results from a systematic review. *Psychooncology* 2011;20(9):909-18. doi: 10.1002/pon.1844 [published Online First: 2010/09/08]
25. Spark LC, Reeves MM, Fjeldsoe BS, et al. Physical activity and/or dietary interventions in breast cancer survivors: a systematic review of the maintenance of outcomes. *Journal of cancer survivorship : research and practice* 2013;7(1):74-82. doi: 10.1007/s11764-012-0246-6 [published Online First: 2012/11/28]
26. Zeng Y, Huang M, Cheng AS, et al. Meta-analysis of the effects of exercise intervention on quality of life in breast cancer survivors. *Breast cancer (Tokyo, Japan)* 2014;21(3):262-74. doi: 10.1007/s12282-014-0521-7 [published Online First: 2014/02/27]
27. Huang HP, He M, Wang HY, et al. A meta-analysis of the benefits of mindfulness-based stress reduction (MBSR) on psychological function among breast cancer (BC) survivors. *Breast cancer (Tokyo, Japan)* 2016;23(4):568-76. doi: 10.1007/s12282-015-0604-0 [published Online First: 2015/03/31]
28. Bourke L, Boorjian SA, Briganti A, et al. Survivorship and improving quality of life in men with prostate cancer. *Eur Urol* 2015;68(3):374-83. doi: 10.1016/j.eururo.2015.04.023 [published Online First: 2015/05/06]

For peer review only - http://bmjopen.bmj.com/site/about/guidelines.xhtml

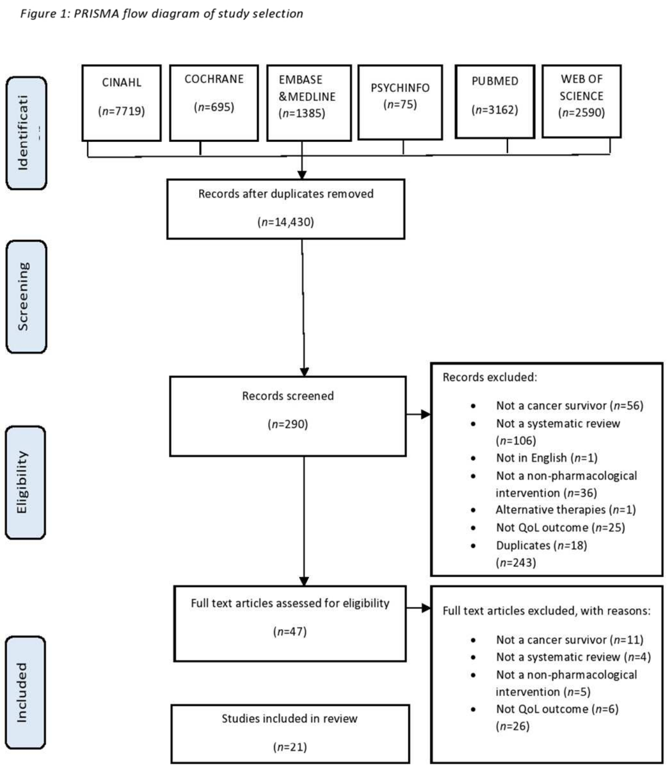

Figure 1: PRISMA Flowchart

104x148mm (300 x 300 DPI)

ONLINE SUPPLMENT: Excluded papers from the full paper search

Author	Date	Reference	Reason for exclusion
Kirshbaum et al.	2007	Kirshbaum MN. A review of the benefits of whole body exercise during and after treatment for breast cancer. *Journal of clinical nursing*. 2007.	This review was excluded as it was not a systematic review.
Badr et al.	2013	Badr H, Krebs P. A systematic review and meta-analysis of psychosocial interventions for couples coping with cancer. *Psycho-Oncology.* 2013.	This review was excluded as it was not cancer survivors, who have completed active treatment.
Jones et al.	2006	Jones LW, Demark-Wahnefried W. Diet, exercise, and complementary therapies after primary treatment for cancer. *The lancet oncology.* 2006.	This review was excluded as it was not systematic.
Burden et al.	2014	Burden S, Gibson DJ, Todd C, Gratton EK, Pilling M, Lal S. Dietary interventions for adult cancer survivors. *The Cochrane Library.* 2014.	This review was excluded as it was a protocol paper. We emailed the authors however, we were unable to find paper.
Keesing et al.	2015	Martin TA, Moran-Kelly RM, Roberts LM, Powe JG, Farrell SN, Singleton J. Effectiveness of individualized survivorship care plans on quality of life of adult female breast cancer survivors: a systematic review (provisional abstract). *JBI Database of systematic reviews and implementation reports.* 2015.	This paper was not a non-pharmacological psychosocial intervention.
Luckett et al.	2011	Luckett T, Britton B, Clover K, Rankin NM. Evidence for interventions to improve psychological outcomes in people with head and neck cancer: a systematic review of the literature. *Supportive care in cancer*. 2011.	This review was excluded as it was not cancer survivors, who have completed active treatment.
Friendenreich and Courneya	1996	Friedenreich CM, Courneya KS. Exercise as rehabilitation for cancer patients (structured abstract). *Clinical Journal of Sport Medicine*. 1996.	This review was excluded as it was a structured abstract only. We emailed the authors however, we were unable to find paper
Cheng et al.	2014	Cheng KK, Lim YT, Koh ZM, Tam WW. Home-based multidimensional survivorship programmes for breast cancer survivors. *The Cochrane Library.* 2014.	This review was excluded as it was a protocol paper. We emailed the authors however, we were unable to find paper.

For peer review only - http://bmjopen.bmj.com/site/about/guidelines.xhtml

de Boer et al.	2011	De Boer AG, Taskila T, Tamminga S, Frings-Dresen M, Feuerstein M, Verbeek J. Interventions to en-hance return-to-work for cancer patients. *Cochrane Database Syst Rev.* 2011.	This paper was excluded as QOL was not main outcome and it was not cancer survivors who had completed active treatment.
Solloway et al.	2016	Yan JH, Pan L, Zhang XM, Sun CX, Cui GH. Lack of efficacy of Tai Chi in improving quality of life in breast cancer survivors: a systematic review and meta-analysis (provisional abstract). *Asian Pacific journal of cancer prevention: APJCP*. 2014.	This review was excluded as it was a provisional abstract only. We emailed the authors however, we were unable to find paper
Ledesma et al.	2009	Ledesma D, Kumano H. Mindfulness-based stress reduction and cancer: a meta-analysis. *Psycho-Oncology.* 2009.	This review was excluded as it was not cancer survivors, who have completed active treatment.
Scott et al.	2013	Scott DA, Mills M, Black A, Cantwell M, Campbell A, Cardwell CR, Porter S, Donnelly M. Multidimensional rehabilitation programmes for adult cancer survivors. *The Cochrane Library.* 2013.	This paper was excluded as QOL was not main outcome
Khan et al.	2013	Khan F, Amatya B, Ng L, Drummond K, Olver J. Multidisciplinary rehabilitation after primary brain tumour treatment. *Cochrane Database syst rev.* 2013.	This review was excluded as it was not cancer survivors, who have completed active treatment.
Budhrani et al.	2014	Budhrani P. Optimal Timing of Mindfulness-Based Stress Reduction in Cancer: Research Synthesis and State of the Science. *The Journal of Alternative and Complementary Medicine.* 2014.	We were unable to access the full paper. Authors confirmed this was a presentation rather than a paper.
Oldervoll et al.	2004	Oldervoll LM, Kaasa S, Hjermstad MJ, Lund JÅ, Loge JH. Physical exercise results in the improved subjective well-being of a few or is effective rehabilitation for all cancer patients? (provisional abstract). *European Journal of Cancer.* 2004.	This review was excluded as it was not cancer survivors, who have completed active treatment. It included only two primary papers with cancer survivors, and not analysed separately.
Casellas-Grau et al.	2014	Casellas-Grau A, Font A, Vives J. Positive psychology interventions in breast cancer. A systematic review. *Psycho-Oncology*. 2014.	This review was excluded as it was not cancer survivors, who have completed active treatment.

For peer review only - http://bmjopen.bmj.com/site/about/guidelines.xhtml

Hulbert-Williams et al.	2015	Hulbert-Williams NJ, Storey L, Wilson KG. Psychological interventions for patients with cancer: psychological flexibility and the potential utility of Acceptance and Commitment Therapy. *European journal of cancer care.* 2015.	This review was excluded as it was not cancer survivors, who have completed active treatment.
Harder et al.	2012	Harder H, Parlour L, Jenkins V. Randomised controlled trials of yoga interventions for women with breast cancer: a systematic literature review. *Supportive care in cancer.* 2012.	This review was excluded as it was not cancer survivors, who have completed active treatment.
De Backer et al.	2009	De Backer IC, Schep G, Backx FJ, Vreugdenhil G, Kuipers H. Resistance training in cancer survivors: a systematic review (provisional abstract). *International journal of sports medicine.* 2009.	This paper was excluded as QOL was not main outcome.
Archer et al.	2015	Archer S, Buxton S, Sheffield D. The effect of creative psychological interventions on psychological outcomes for adult cancer patients: a systematic review of randomised controlled trials. *Psycho-Oncology.* 2015.	This review was excluded as it was not cancer survivors, who have completed active treatment.
Piet et al.	2012	Piet J, Würtzen H, Zachariae R. The effect of mindfulness-based therapy on symptoms of anxiety and depression in adult cancer patients and survivors: A systematic review and meta-analysis. *Journal of Consulting and Clinical Psychology.* 2012.	This paper was excluded as QOL was not main outcome.
Cramp et al.	2010	Cramp F, James A, Lambert J. The effects of resistance training on quality of life in cancer: a systematic literature review and meta-analysis. *Supportive care in cancer*. 2010.	This review was excluded as it was not cancer survivors, who have completed active treatment.
Stan et al.	2012	Stan DL, Collins NM, Olsen MM, Croghan I, Pruthi S. The evolution of mindfulness-based physical interventions in breast cancer survivors. *Evidence-Based Complementary and Alternative Medicine*. 2012.	This paper was excluded as it is not a systematic review
Bouma et al.	2015	Bouma G, Admiraal JM, de Vries EG, Schröder CP, Walenkamp AM, Reyners AK. Internet-based support programs to alleviate psychosocial and physical	This review was excluded as it was not cancer survivors, who have completed active treatment.

For peer review only

		symptoms in cancer patients: a literature analysis. *Critical reviews in oncology/hematology.* 2015.	
Bourke et al.	2016	Bourke L, Smith D, Steed L, Hooper R, Carter A, Catto J, Albertsen PC, Tombal B, Payne HA, Rosario DJ. Exercise for men with prostate cancer: a systematic review and meta-analysis. *European urology.* 2016.	This review was excluded as it was not cancer survivors, who have completed active treatment.
Post et al.	2016	Post KE, Flanagan J. Web based survivorship interventions for women with breast cancer: An integrative review. European *Journal of Oncology Nursing*. 2016.	This paper was excluded as QOL was not main outcome.

For peer review only

Annex1: Full search strategy

Component 1: Population

#1. Neoplasms (MeSH term) or cancer or cancers or cancerous or carcinoma* or neoplas* or tumor* or tumour* or malignan*

Component 2: Intervention

#2. Counseling (MeSH term) or psychotherapy (MeSH term) or "cognitive therapy" (MeSH term) or "self-help groups" (MeSH term) or "mind body therapies" (MeSH term) or "behavior therapy" (MeSH term) or psychotherapy, group (MeSH term) or meditation, (MeSH term) or "mindfulness" (MeSH term) behaviour therapies, cognitive (MeSH term)

#3. (counsel*:ti,ab or psychoeducat*:ti,ab or educat*:ti,ab or coping*:ti,ab or psychological*:ti,ab or psychosocial*:ti,ab or psychotherap*:ti,ab or psychoanalytic*:ti,ab) AND (therap*:ti,ab or treatment*:ti,ab or outcome*:ti,ab or intervention*:ti,ab)

#4. (social: ti,ab or peer: ti,ab or group: ti,ab) AND (support: ti,ab)

#5. self:ti,ab AND help:ti,ab

#6. (cognitive:ti,ab or behav*:ti,ab) AND (treatment*:ti,ab or therap*:ti,ab)

#7. "CBT":ti,ab

#8. (Family:ti,ab or couple:ti,ab) AND (therap*:ti,ab)

#9. meditation:ti,ab or mindfulness:ti,ab

#10. #2 or#3or#4or#5or#6or#7or#8+or#9

Component 3: Outcome

#11. "quality of life" (MeSH term) or "well being": ti,ab or "QoL" (all fields) or "quality of life": ti,ab

FULL PICO:

#1 AND #11 AND #12

Filters: Humans, English language, Reviews, Age group

For peer review only

RISMA Checklist

	#	Checklist item
	1	Identify the report as a systematic review, meta-analysis, or both.
ary	2	Provide a structured summary including, as applicable: background; objectives; data sources; study eligibility criteria, participants, and interventions; study appraisal and synthesis methods; results; limitations; conclusions and implications of key findings; systematic review registration number.
	3	Describe the rationale for the review in the context of what is already known.
	4	Provide an explicit statement of questions being addressed with reference to participants, interventions, comparisons, outcomes, and study design (PICOS).
tration	5	Indicate if a review protocol exists, if and where it can be accessed (e.g., Web address), and, if available, provide registration information including registration number.
	6	Specify study characteristics (e.g., PICOS, length of follow-up) and report characteristics (e.g., years considered, language, publication status) used as criteria for eligibility, giving rationale.
es	7	Describe all information sources (e.g., databases with dates of coverage, contact with study authors to identify additional studies) in the search and date last searched.
	8	Present full electronic search strategy for at least one database, including any limits used, such that it could be repeated.
	9	State the process for selecting studies (i.e., screening, eligibility, included in systematic review, and, if applicable, included in the meta-analysis).
ocess	10	Describe method of data extraction from reports (e.g., piloted forms, independently, in duplicate) and any processes for obtaining and confirming data from investigators.
	11	List and define all variables for which data were sought (e.g., PICOS, funding sources) and any assumptions and simplifications made.
vidual studies	12	Describe methods used for assessing risk of bias of individual studies (including specification of whether this was done at the study or outcome level), and how this information is to be used in any data synthesis.

For peer review only

RISMA Checklist

es	13	State the principal summary measures (e.g., risk ratio, difference in means).
s	14	Describe the methods of handling data and combining results of studies, if done, including measures of consistency (e.g., I^2) for each meta-analysis.

Page 1 of 2

	#	Checklist item
studies	15	Specify any assessment of risk of bias that may affect the cumulative evidence (e.g., publication bias, selective reporting within studies).
s	16	Describe methods of additional analyses (e.g., sensitivity or subgroup analyses, meta-regression), if done, indicating which were pre-specified.
	17	Give numbers of studies screened, assessed for eligibility, and included in the review, with reasons for exclusions at each stage, ideally with a flow diagram.
ics	18	For each study, present characteristics for which data were extracted (e.g., study size, PICOS, follow-up period) and provide the citations.
studies	19	Present data on risk of bias of each study and, if available, any outcome level assessment (see item 12).
al studies	20	For all outcomes considered (benefits or harms), present, for each study: (a) simple summary data for each intervention group (b) effect estimates and confidence intervals, ideally with a forest plot.
s	21	Present results of each meta-analysis done, including confidence intervals and measures of consistency.

For peer review only

RISMA Checklist

studies	22	Present results of any assessment of risk of bias across studies (see Item 15).
s	23	Give results of additional analyses, if done (e.g., sensitivity or subgroup analyses, meta-regression [see Item 16]).
nce	24	Summarize the main findings including the strength of evidence for each main outcome; consider their relevance to key groups (e.g., healthcare providers, users, and policy makers).
	25	Discuss limitations at study and outcome level (e.g., risk of bias), and at review-level (e.g., incomplete retrieval of identified research, reporting bias).
	26	Provide a general interpretation of the results in the context of other evidence, and implications for future research.
	27	Describe sources of funding for the systematic review and other support (e.g., supply of data); role of funders for the systematic review.

Li[illegible]erati A, Tetzlaff J, Altman DG, The PRISMA Group (2009). Preferred Reporting Items for Systematic Reviews and Meta-Analyses: The PRISMA State
do[illegible]10.1371/journal.pmed1000097

For more information, visit: **www.prisma-statement.org**.

For peer review only

BMJ Open

A Review of Systematic Reviews of non-pharmacological interventions to improve quality of life in cancer survivors.

Journal:	*BMJ Open*
Manuscript ID	bmjopen-2017-015860.R2
Article Type:	Research
Date Submitted by the Author:	30-Aug-2017
Complete List of Authors:	Duncan, Morvwen; Barts and The London School of Medicine & Dentistry Queen Mary University of London, Centre for Psychiatry at the Wolfson Institute of Preventive Medicine<br>Moschopoulou, Elisavet; Barts and The London School of Medicine & Dentistry Queen Mary University of London, Centre for Psychiatry at the Wolfson Institute of Preventive Medicine<br>Herrington, Eldrid; Barts and The London School of Medicine & Dentistry Queen Mary University of London, Centre for Psychiatry at the Wolfson Institute of Preventive Medicine<br>Deane, Jennifer; Barts and The London School of Medicine & Dentistry Queen Mary University of London, Centre for Psychiatry at the Wolfson Institute of Preventive Medicine<br>Roylance, Rebecca ; University College London<br>Jones, Louise; University College Medical School, Marie Curie Palliative Care Unit, UCL Mental Health Sciences Unit<br>Bourke, Liam; University of Sheffield Medical School<br>Morgan , Adrienne ; Queen Mary University of London - Charterhouse Square Campus, Barts Cancer Institute<br>Chalder, Trudie; King's College London, Psychological Medicine<br>Thaha, Mohamed; Queen Mary's University of London, National Centre for Bowel Research & Surgical Innovation<br>Taylor, Stephanie; Barts and The London School of Medicine & Dentistry Queen Mary University of London, Centre for Psychiatry at the Wolfson Institute of Preventive Medicine<br>Korszun, Ania; Barts and The London School of Medicine and Dentistry, Psychiatry<br>White, Peter; Barts and the London School of Medicine, Queen Mary University of London, Centre for Psychiatry<br>Bhui, Kamaldeep; Barts and The London School of Medicine & Dentistry Queen Mary University of London, Centre for Psychiatry at the Wolfson Institute of Preventive Medicine
**Primary Subject Heading**:	Oncology
Secondary Subject Heading:	Public health, Patient-centred medicine
Keywords:	Adult oncology < ONCOLOGY, Quality of life, Psychosocial interventions

For peer review only

A Review of Systematic Reviews of non-pharmacological interventions to improve quality of life in cancer survivors.

Morvwen Duncan, Elisavet Moschopoulou, Eldrid Herrington, Jennifer Deane, Rebecca Roylance, Louise Jones, Liam Bourke, Adrienne Morgan, Trudie Chalder, Mohamed A. Thaha, Stephanie Taylor, Ania Korszun, Peter White, *Kamaldeep Bhui on behalf of SURECAN Investigators.

§Morvwen Duncan

Research Assistant

Academic Psychological Medicine, Wolfson Institute of Preventive Medicine, Barts and the London School of Medicine and Dentistry, Queen Mary University of London, EC1A 7BE

§Elisavet Moschopoulou

Postgraduate Researcher

Centre for Psychiatry, Barts and The London School of Medicine and Dentistry, Queen Mary University of London, Old Anatomy Building, Charterhouse Square, London EC1M 6BQ

Eldrid Herrington

Honorary Senior Research Fellow

Blizard Institute, National Bowel Research Centre, Queen Mary University of London, 2 Newark Street, London E1 2AT

Public Patient representative

Department of Colorectal Surgery, The Royal London Hospital, Barts Health NHS Trust, Whitechapel, London E1 1BB

Jennifer Deane

Research Assistant

Academic Psychological Medicine, Wolfson Institute of Preventive Medicine, Barts and the London School of Medicine and Dentistry, Queen Mary University of London, EC1A 7BE

Rebecca Roylance

Consultant Medical Oncologist and Honorary Senior Lecturer

University College Hospitals NHS Foundation Trust and UCLH Biomedical Research Centre, 149 Tottenham Court Road, London W1T 7DN

Louise Jones

Clinical Senior Lecturer

Marie Curie Palliative Research Department, Division of Psychiatry, UCL, 149 Tottenham Court Road, London W1T 7NF

Liam Bourke

Reader in Clinical Science

For peer review only - http://bmjopen.bmj.com/site/about/guidelines.xhtml

Sheffield Hallam University, Howard Street, Sheffield S1 1WB.

Adrienne Morgan

Honorary Senior Lecturer

Centre for Tumour Biology, Barts Cancer Institute - Queen Mary University of London, Old Anatomy Building, London EC1M 6BQ

Trudie Chalder

Professor of Cognitive Behavioural Psychotherapy

Department of Psychological Medicine, King's College London, Denmark Hill, King's College, London, SE59RJ

Mohamed A Thaha

Senior Lecturer & Consultant in Colorectal Surgery

Blizard Institute, National Bowel Research Centre,
Barts and the London School of Medicine & Dentistry, Queen Mary University of London, 2 Newark Street, London E1 2AT.
Department of Colorectal Surgery, The Royal London Hospital, Barts Health NHS Trust, Whitechapel, London E1 1BB

Stephanie Taylor

Professor in Public Health and Primary Care

Centre for Primary Care and Public Health, Blizard Institute, Barts and The London School of Medicine and Dentistry, London, E1 2AB

Ania Korszun

Professor of Education and Psychiatry

Wolfson Institute of Preventive Medicine, Barts and the London School of Medicine and Dentistry, Queen Mary University of London, EC1M 6BQ

Peter White

Professor of Psychological Medicine

Wolfson Institute of Preventive Medicine, Barts and the London School of Medicine and Dentistry, Queen Mary University of London, EC1A 7BE

* Kamaldeep Bhui

Professor of Cultural Psychiatry & Epidemiology

Wolfson Institute of Preventive Medicine, Barts and the London School of Medicine and Dentistry, Queen Mary University of London, EC1A 7BE

& SURECAN Research Group

*Correspondence to: Kamaldeep Bhui, k.s.bhui@qmul.ac.uk, 020 7882 2012

For peer review only - http://bmjopen.bmj.com/site/about/guidelines.xhtml

§ Joint first authors

Key Words: *Cancer, quality of life, interventions*

Word Count:

Paper excluding abstract, tables and references: 4795

Abstract: 296

For peer review only

For peer review only - http://bmjopen.bmj.com/site/about/guidelines.xhtml

ABSTRACT

Objectives

Over two million people in the UK are living with and beyond cancer. A third report diminished quality of life.

Design

A review of published systematic reviews to identify effective non-pharmacological interventions to improve the quality of life of cancer survivors

Data Sources

Databases searched until May 2017 included PubMed, Cochrane Central, EMBASE, MEDLINE, Web of Science, the Cumulative Index to Nursing and Allied Health Literature (CINAHL) and Psych INFO.

Study selection

Published systematic reviews of randomised trials of non-pharmacological interventions for people living with and beyond cancer were included; included reviews targeted patients aged over 18. All participants had already received a cancer diagnosis. Interventions located in any healthcare setting, home or online were included. Reviews of alternative therapies or those non-English reports were excluded. Two researchers independently assessed titles, abstracts, the full text of papers, and independently extracted the data.

Outcomes

The primary outcome of interest was any measure of global (overall) quality of life.

Analytic methods

Quality assessment (AMSTAR) and narrative synthesis, evaluating effectiveness of non-pharmacological interventions and their components.

For peer review only - http://bmjopen.bmj.com/site/about/guidelines.xhtml

For peer review only

Results

Of 14,430 unique titles, 21 entered the review of reviews. There was little overlap in the primary papers across these reviews. 13 reviews covered mixed tumour groups, seven focused on breast cancer, and one focused on prostate cancer. Face-to-face interventions were often combined with online, telephone and paper-based reading materials. Interventions included physical, psychological or behavioural, multidimensional rehabilitation and online approaches. Yoga specifically, physical exercise, more generally, cognitive behavioural therapy (CBT) and mindfulness-based stress reduction (MBSR) programmes showed benefit in terms of quality of life.

Conclusions

Exercise-based interventions were effective in the short (less than 3-8 months) and long term. CBT and MBSR also showed benefits, especially in the short term. The evidence for multidisciplinary, online, and educational interventions was equivocal.

For peer review only

ARTICLE SUMMARY

Strengths and limitations of this study

- This is a systematic review of reviews and evidence synthesis of non-pharmacological interventions in cancer survivors.
- Longer term studies are needed and studies of greater methodological quality that adopt similar reporting standards.
- Definitions of survivor varied and more studies are needed for different types of cancer, and specifically for patients who have poor quality of life.
- More studies are needed that investigate educational, online and multidisciplinary team based interventions.
- This review has some limitations in the methodology. Studies not in English and grey literature were not included. This was a review of reviews: we did not review individual studies focussed on specific cancers or stage and we did not re-assess the quality of the primary studies included in each review.

INTRODUCTION

For peer review only - http://bmjopen.bmj.com/site/about/guidelines.xhtml

Advances in public awareness, early detection and improved treatments mean that more people are now living with and beyond cancer. For example, Cancer Research UK reports that 50% of people diagnosed with cancer in England and Wales survive 10 years or more, and survival rates have doubled over the last 40 years.[1] This group of survivors includes people at various stages of active treatment, and those in remission, who are gradually restoring their social and occupational roles.

A significant proportion of cancer survivors experience poor quality of life.[2] The main causes of poor quality of life include depression, anxiety, distress, fear of recurrence, lower levels of social support; impacts on relationships, family, and social function; psychological and social needs, and problems coping.[2 3] The process of diagnosis and treatment is traumatic and disruptive. It is not unusual for cancer patients to experience distress. Common experiences for those living with and beyond cancer include reduced physical ability, fatigue, changes in sexual activity and developing other medical conditions that affect function for many years.[2 3] If a person is suffering from fatigue, depression or anxiety they are understandably less motivated to visit friends or engage in social activities; the strain on marital relationships may lead to a loss of support: 25% of people who experience difficulties have broken up with their partner as a result of cancer.[3 4] Thus, the effects of cancer extend beyond the diagnostic and active treatment phases. This review aims to gather the evidence for practitioners, patients and their carers about effective non-pharmacological interventions to improve quality of life in cancer survivors. We sought to summarise the effectiveness of non-pharmacological interventions in cancer survivors as part of a NIHR funded programme development grant to inform the design and delivery of a full programme grant.

METHODS

For peer review only - http://bmjopen.bmj.com/site/about/guidelines.xhtml

This review of reviews examined existing systematic reviews of non-pharmacological interventions that include information on quality of life of those living with and beyond cancer.

Inclusion and exclusion criteria

The study included any systematic reviews that explicitly reported randomised controlled trials. Inclusion criteria were organised in accordance with the PICO reporting structure (see Table 1). The population of interest was people living with and beyond cancer, who were aged 18 years or more, and who had received their cancer diagnosis as adults.

We defined non-pharmacological interventions as those that did not involve any drug or medicine, but they could include educational, behavioural, psychosocial approaches or physical activity; we excluded complementary and alternative therapies as defined by the NHS Choices resource.[5] However, we included physical activity and psychological approaches that were part of yoga based interventions after consulting with patients in the development of the review. Comparators were not specified for the purpose of the inclusion criteria of the review of reviews, but comparators reported in the original reviews were considered in the analysis.

The primary outcome was quality of life (QoL) defined by physical, psychological and social functioning. We reported on studies that used an established and validated measure of global or overall QoL; some of these are cancer specific. In the literature, the terms 'Quality of Life' and 'Health Related QoL' are used interchangeably; therefore both are included under the term 'QoL' in this review. The study settings included any healthcare venue, such as hospital inpatient or outpatient services, community services and also included home and remote e-technology based interventions.

For peer review only - http://bmjopen.bmj.com/site/about/guidelines.xhtml

For peer review only

Table 1: Application of the PICO search strategy

**Population**	Participants living beyond cancer, who have completed active treatment with curative intent; aged 18 or more who received their cancer diagnosis in adulthood.
**Intervention**	Non-pharmacological interventions. Psychological, social and physical activity, excluding complementary and alternative therapies or medicines. Including yoga interventions with meditation, activity or mindfulness.
**Outcomes**	Quality of life.
**Setting**	Any healthcare setting: hospital (in-patient or outpatient), community or remote (e.g. using e-technology).
**Study Design**	Systematic reviews that had explicitly searched for RCTs. To be classified as a systematic review the following criteria were met:<br>- clear inclusion criteria<br>- a systematic search strategy<br>- a screening procedure to identity relevant studies<br>- systematic data extraction and analysis procedures for RCTs

Data sources

We searched the following databases: PubMed, Cochrane Central, EMBASE, MEDLINE, Web of Science, The Cumulative Index to Nursing and Allied Health Literature (CINAHL) and Psych INFO. The final search was from inception to May 2017 and is shown in Annex 1. We consulted experts in the field to assess completeness of the list of identified reviews, and where necessary, contacted authors to secure the full text versions.

Study selection

For peer review only - http://bmjopen.bmj.com/site/about/guidelines.xhtml

Two authors (MD, JD) independently screened all titles and abstracts of studies identified by the search strategy against inclusion and exclusion criteria, and, when eligibility was determined, the full text was read. Discrepancies around inclusion were resolved by discussion or in consultation with a third author when required (KB). We searched the reference lists of all included reviews to identify any further relevant reviews. The research team was not blinded to authors. Citations were downloaded and managed in an Endnote library.

For peer review only

Data extraction

Two authors (EM, EH) independently extracted data from each of the eligible reviews into a purpose built, pre-designed, structured template. The data extraction forms were then summarised in a table and reviewed independently by a third reviewer (KB) Extracted data included the following information:

- Publication details: author, year, title, journal, country, format of publication.
- Study characteristics: number of primary studies, total number of participants, range of publication dates, gender, age range of participants and socioeconomic data, primary cancer site, length of time since final cancer treatment and type of treatment.
- Intervention design and evaluation: setting, description of the intervention and its components: physical components, psychosocial components, educational components; duration of intervention, follow up, number of treatment contacts, type of practitioner providing treatment, mode of delivery of intervention, and any outcomes.
- Documents: Availability of treatment manuals.
- Results: Main outcome measures, secondary outcome measures, narrative findings, adherence levels, patient satisfaction, effect sizes against intervention components.

Assessment of methodological quality of included reviews

The methodological quality of the systematic reviews was evaluated using AMSTAR[6], a measurement tool for the assessment of multiple systematic reviews that has good reliability and validity (Table 2). The AMSTAR checklist used can be found here: https://amstar.ca/Amstar_Checklist.php.

Data analysis and narrative synthesis

The intervention components were listed, followed by a narrative synthesis.[7] This included understanding components of the interventions, exploring patterns of findings across studies and within primary reviews, and giving greater weight to studies of higher quality in the interpretation of the findings, especially if there were contradictions between the findings of reviews. Ultimately, the purpose was to put into text format the key findings from the most robust evidence available, to guide treatment and future research recommendations. The synthesis set out reported effect sizes across studies, means and standard deviation. Meta-analysis was not undertaken, due to heterogeneity of methods, outcomes, and absence of reported effect sizes (10 reviews did not provide effect sizes). The publications were segmented into those reporting meta-analyses to which the greatest weighting was given in the synthesis; some reviews did not undertake or report meta-analyses but rather reported each study, trends and the range of effect sizes; a third group reported no effect sizes but provided narrative statements.

Patient and Public Involvement

Patients and carers (and respective organisations) were involved in the design and development of the programme development grant application (from which this review is one output). Patients and carers attended all the steering group meetings and were an integral part of the research team, commenting on and critiquing the inclusion and exclusion criteria, outcome selection, and the acceptability and likely value of interventions. As part of the steering group, they received and commented on study progression, emergent findings, and reports. They are integral to the dissemination plans including sharing the publication, but also helping craft lay summaries of the

For peer review only - http://bmjopen.bmj.com/site/about/guidelines.xhtml

overall research project and key findings. A Public-Patient representative (EH) performed the data extraction together with research and clinical colleagues and co-authored and edited the review. Public Patient representatives were also part of the steering group and informed the design and delivery of the review.

RESULTS

Study selection

Electronic database searches yielded 14,430 unique reviews. From this 290 were included from the title search, followed by 47 from the abstract search. After scrutinising the full texts, 21 of eligible published reviews entered this review (Figure 1). The 26 studies excluded studies are listed in an online supplementary file. The quality scores are shown in Table 2.

For peer review only

For peer review only - http://bmjopen.bmj.com/site/about/guidelines.xhtml

Figure 1: PRISMA flow diagram of study selection

FIGURE 1 ABOUT HERE

Table 2: AMSTAR, tool for the assessment of multiple systematic reviews

Review	AMSTAR Score*	Quality Rating
Bourke et al, 2015	3	Low
Buffart et al, 2012	6	Moderate
Cramer et al, 2012	9	High
Culos-Reed et al, 2012	3	Low
Duijts et al, 2010	4	Moderate
Ferrer et al, 2011	8	High
Fong et al, 2012	8	High
Fors et al, 2011	5	Moderate
Galvão et al, 2005	2	Low
Gerritsen and Vincent 2015	6	Moderate
Huang et al, 2015	8	High
Khan et al, 2012	10	High
McAlpine et al, 2015	5	Moderate
Mewes et al, 2012	5	Moderate
Mishra et al, 2012	10	High
Osborn et al, 2006	7	Moderate
Smits et al, 2015	8	High
Spark et al, 2013	6	Moderate
Spence et al, 2010	5	Moderate
Zachariae et al. 2015	5	Moderate
Zeng et al, 2014	6	Moderate

**The maximum score on AMSTAR is 11 and scores of 0-3 indicate that the review is of low quality, 4-7 of moderate quality; and of 8-11 as high quality*

For peer review only - http://bmjopen.bmj.com/site/about/guidelines.xhtml

Study characteristics

The types of interventions, settings, cancer type, measures of quality of life, and the key narrative findings are reported in Table 3.

Participants

The number of patients included in the reviews ranged from 262[8] to 7164.[9] 13 reviews covered mixed tumour groups,[10-22] seven specifically focused on breast cancer,[8 9 23-27] and one on prostate cancer.[28]

Intervention Type and Components

Face-to-face delivery of interventions was often combined with online delivery (three reviews)[9 24 28]; others included telephone communication (five reviews)[9 11 23 25 26] and printed information (two reviews).[11 25] Four reviews included interventions that provided supplementary compact discs, manuals or video tools.[11 23 24 28] Two reviews were from inpatient rehabilitation.[8 18] None of the reviews reported the use of structured manuals, and interventions were often not fully described or broken down into different components, nor was there attention to a mechanism or theory of change.

Ten of the reviews focused on physical interventions,[10 12 13 16 19-21 25 26 28] and three focused on yoga; [11 14 23] four reviews were of psychosocial or behavioural interventions; [9 17 24 27] and one review focused on online interventions including connecting patients and online education (see Tables 3 & 4).[15] One review compared multi-dimensional versus mono-dimensional interventions [18] and one tested multidisciplinary rehabilitation models. [8] Finally, one review focused on the effects of expressive writing. [22] The duration and frequency of the interventions varied greatly from a single 20 minute session [17] to 60 weekly sessions. [10]

For peer review only - http://bmjopen.bmj.com/site/about/guidelines.xhtml

The most common component of physical interventions was aerobic exercise, [9 10 12 13 16 19 26] and resistance/strength training. [9 10 12 13 16 26] Psychological education [8 9 17 18 24] and cognitive behaviour therapy [9 17 18 24] were the most utilised psychological and educational interventions. Peer support was often used as a psychological and a behavioural intervention. [8 9 15] Components of the interventions were thematically organised into two groups (see Table 4 for a more detailed itemisation): biological or physical actions (19 types of activity or diet change) and psychological, behavioural or educational (24 types of intervention about mind and body including cognitive behavioural therapy, mindfulness-based stress reduction, psychosexual therapy, supporting existing coping methods, emotional support, relaxation, psychotherapy and psychosocial therapy, and interventions focusing on social support, guided imagery, self-management, use of peer support, bibliotherapy, telephone and web-based interventions, return to work interventions).

For peer review only - http://bmjopen.bmj.com/site/about/guidelines.xhtml

For peer review only

Table 3: Characteristics of included reviews

Review	Aims of review	Number of primary studies	Participants	Definition of 'survivor'	Setting	Intervention, duration and frequency	Outcome - QoL measures	Narrative findings
Buffart et al, 2012	Systematic review of RCTs and meta-analysis of the effects of yoga in cancer patients and survivors.	16 publications/13 RCTs	744 breast cancer patients and 39 lymphoma cancer patients during and after treatment. Mean age range: 44-63 years.	Patients during and after treatment.	Face to face, with supplementary booklets and audiotapes of exercises for home practice	All included a yoga program led by experienced yoga instructors with physical poses (asanas), breathing techniques, (pranayama), and relaxation or meditation (savasana or dhanya). Programme duration: 6 weeks to 6 months.	FACT-G, SF-36, EORTC QLQ-C30, EORTC QLQ-C30, FLIC	Yoga has strong beneficial effects on distress, anxiety and depression, moderate effects on fatigue, general HRQoL, emotional function and social function, small effects on functional well-being, and no significant effects on physical function and sleep disturbances.
Bourke et al, 2015	To evaluate the evidence from RCTs of supportive interventions designed to improve prostate cancer-specific quality of life.	20 RCTs	2,654 prostate cancer survivors	Patients during and after treatment.	Group or face to face, online or with supplementary audiotapes	Lifestyle interventions including exercise interventions, diet interventions or a combination of exercise and diet. Multidisciplinary group education or online education and support. Enhanced standard care interventions and cognitive behavioural interventions. Varied durations and follow up frequencies.	FACT-P, QLQ-PR25, EPIC, EPIC-26, UCLA-PCI, PCa-QoL	Supervised and individually tailored patient-centred interventions such as lifestyle programmes are beneficial.

For peer review only - http://bmjopen.bmj.com/site/about/guidelines.xhtml

Cramer et al, 2012	To systematically assess and meta-analyse the evidence for the effects of yoga on HRQoL nd psychological health in breast cancer patients and survivors.	12 RCTs were included in the qualitative synthesis and 10 of them were included in the meta-analysis	742 breast cancer patients during or after treatment. Mean age range: 44-63 years.	Those who had completed active treatment before the onset of the study.	Face to face, with supplementary audio and video tools or telephone calls.	Yoga interventions including Iyengar yoga, Yoga of Awareness, Viniyoga, restorative yoga, yoga based on Patanjali's yoga tradition, Yoga in Daily Life, integrated yoga and hatha yoga. Duration: 1 week to 6 months. Frequency varied from daily sessions to weekly.	FACT-G, FACT-B, FACIT-Sp, SF-36, SF-12, FLIC, EORTC QLQ-C30	There is moderate evidence for the short-term effect of yoga on global HRQoL. However these short-term effects could not be clearly distinguished from bias.
Culos-Reed et al, 2012	To determine the clinical significance of patient-reported outcomes from yoga interventions conducted with cancer survivors.	13 studies/ 7 RCTs	474 mixed cancer patients. The majority were breast cancer patients during and after treatment. RCTs: sample size in the treatment group at time 2 ranged from 13-45 patients. Mean age range: 46–60 years.	Patients both on and off treatment.	Face to face	Yoga styles included hatha, integral, iyengar, tibetan, viniyoga, and vivekananda. Duration: 6 to 26 weeks. Frequency varied from 5 times per week to weekly and classes were 60–90 minutes.	SF-36, EORTC QLQ-C30, FACT-B, FACT-G, SF-12, NHP	Yoga for cancer survivors results in clinically significant improvements in overall HRQoL, as well as in its mental and emotional domains.

For peer review only

Duijts et al, 2011	Evaluate the effect of behavioural techniques and physical exercise on psychosocial functioning and HRQoL in breast cancer patients and survivors.	56 RCTs	>7,000 breast cancer patients, including non-metastatic and metastatic patients during and after treatment. Participants' ages were not specified.	Patients during and after treatment.	Face to face, online or by telephone, individually or at group level	Behavioural techniques included psycho-education, problem solving, stress management, CBT, relaxation techniques, social and emotional support.<br>Physical interventions included yoga, self-management exercise protocol, aerobic or resistance exercise training and dance movement.<br>Intervention duration varied from 1-56 weeks of 3-56 sessions.	SIP, CARES, ABS, EORTC QLQ-C30, FACT-B, FACT-G, FACT-F, FACT-An, FLIC, SF-12, SF-36, QoL-BC, GHQ, SDS, IFS-CA, VAS	No significant effect of behavioural techniques on HRQoL.<br>Physical exercise produced statistically significant but moderate effects on HRQoL.
Ferrer et al, 2011	To examine the efficacy of exercise interventions in improving quality of life in cancer survivors, as well as features that may moderate such effects.	78 studies/ 43 RCTs	3,629 participants: 54% breast cancer, 8% prostate cancer, 2% colorectal cancer, 1% each featured endometrial, head–neck, lymphoma, and ovarian cancer survivors, and 32% included mixed diagnosis. 2,432 patients participated in the RCTs.<br>Mean age was 55 years.	Survivor was defined as post diagnosis.	Supervised or unsupervised	Interventions were designed to affect exercise behaviour by comparing low vs high exercise intensity.<br>36% used trained intervention leaders; 56% featured supervised exercise sessions.<br>The mean level of targeted aerobic METs was 4.2 (SD=2.2), and the mean level of targeted resistance METs was 2.5 (SD=2.2).<br>Duration: 8-26 weeks. The mean length of intervention session was 51.1 mins and the mean number of sessions per intervention was 22.8.	EORTC QLQ-30, SF-36, FACTIT, Quality of Life Index, FACT-G, FACT-An, FACT-B, FACT H&N, FACT-P, FLIC, CARES-SF, Rotterdam QOL, WHOQOL-BREF.	There was a positive effect of physical interventions on QoL, sustained for delayed follow-up assessment.<br>Efficacy increased as the length of intervention decreased, and if exercise was supervised.<br>Targeted aerobic intensity significantly predicted QoL improvements as a quadratic trend.<br>Targeted aerobic METs predicted intervention efficacy.<br>Number of sessions, targeted resistance METs, training of facilitators, and inclusion of flexibility content were not significantly related to QoL outcomes.

For peer review only - http://bmjopen.bmj.com/site/about/guidelines.xhtml

Fong et al, 2012	To systematically evaluate the effects of physical activity in adult patients after completion of main treatment related to cancer.	34 RCTs	3,769 participants; 65% included breast cancer only, 9% colorectal cancer only, 3% endometrial cancer only, and 27% mixed diagnosis. Mean age range: 39-74 years.	Patients who have completed their main cancer treatment but might be undergoing hormonal treatment.	Face to face	Exercise interventions included aerobic exercise, resistance or strength training. Duration: 3 to 60 weeks. Frequency ranged from daily to once a week.	FACT G, FACT-B, FACT-C, EORTC, SF-36	Physical activity was shown to be associated with clinically important positive effects on quality of life. Aerobic plus resistance training was significantly more effective than aerobic training alone on general QoL.
Fors et al, 2011	To determine the effectiveness of psychoeducation, cognitive behavioural therapy (CBT) and social support interventions used in the rehabilitation of breast cancer patients.	18 RCTs	3,272 breast cancer patients, during and post treatment. Age range not specified.	Patients who have finished surgery and adjuvant treatment.	Online, face to face or by telephone or by using print material, individually or in a group	Psychoeducation, CBT and social and emotional support. Duration ranged from 2 weeks – 6 months.	FACT-B, FACT-G, EORTC-QLQ-C30, QoL-BC, QLI, EuroQoL-5D, QoQ-C33 Global	Psychoeducation showed inconsistent results during and after primary treatment. CBT after primary treatment (6-12 weeks) led to improved QoL. CBT during primary treatment had inconsistent results.

For peer review only - http://bmjopen.bmj.com/site/about/guidelines.xhtml

Galvao et al, 2005	To present an overview of exercise interventions in cancer patients during and after treatment and evaluate dose-training response considering type, frequency, volume, and intensity of training along with physiological outcomes.	26 studies/ 9 RCTs	1,186 mixed cancer patients during and post treatment. 458 patients participated in the RCTs. Age range: 14 - 65 years.	Patients during and after treatment.	Face to face	Exercise interventions included a cardiovascular exercise programme and mixed training (cardio, resistance and flexibility exercises). Intensity level when provided was described as between 60-80% maximum heart rate (MHR). Programme duration was 4-28 weeks. Frequency ranged from twice a week to 5 times per week.	Modified Rotterdam QoL Survey.	Contemporary resistance training provides anabolic effects that counteract side effects of cancer treatments, to improve quality of life.
Gerritsen and Vincent, 2015	To evaluate the effectiveness of exercise in improving QoL in patients with cancer, during and after treatment.	16 RCTs	1,845 mixed, breast, lymphoma, colorectal, prostate and lung cancer patients. Aged: 18-79 years	Patients during or after treatment.	Home-based or outdoors, supervised or unsupervised	Exercise modalities included walking, cycling, strength training, swimming, stability training and elliptical training ranging from twice a week to five times a week. The duration ranged from 3 weeks to 16 months.	EORTC-QLQ, FACT-An, FACT-B, FACT-C, FACT-G, FACT-P, SF-36, MCS/PCS	Exercise has a direct positive impact on cancer patients' QoL, during and following medical intervention

For peer review only - http://bmjopen.bmj.com/site/about/guidelines.xhtml

Huang et al, 2015	Meta-analysis to evaluate the benefits of mindfulness-based stress reduction on psychological distress among breast cancer survivors.	9 studies/ 4 RCTs	964 breast cancer survivors. 812 patients participated in the RCTs. Mean age range: 49 - 57.5	Women diagnosed with breast cancer.	Setting not specified	8-week mindfulness based stress reduction program. One study used a 6 week formula.	FACT-B	Mindfulness based stress reduction programmes showed a positive effect in improving psychological function and overall QoL of breast cancer survivors.
Khan et al, 2012	To assess the effects of organised multidisciplinary rehabilitation during follow-up in women treated for breast cancer.	2 RCTs	262 breast cancer patients after treatment. All women were older than 49 years except for two.	At least 12 months after completion of definitive cancer treatment.	Group-based inpatient programme or inpatient programme together with a home-based programme.	Multidisciplinary rehabilitation programme incorporating medical input, psychology and physiotherapy or psychology-based education, exercise, peer support group activity and medical input. Duration: 3 to 10 weeks of 3 sessions per week.	Local QoL measure, EORTC QLQ-C30	There was 'low level' evidence that multidisciplinary rehabilitation can improve QoL over 12 months. Not possible to suggest optimal frequency, or one type of intervention over another.

For peer review only - http://bmjopen.bmj.com/site/about/guidelines.xhtml

McAlpine et al, 2015	To examine the evidence-based literature surrounding the use of online resources for adult cancer patients.	14 studies/ 9 RCTs	2,351 lung, prostate, breast, head and neck and mixed cancer patients. The sample size for the RCTs was 1,121 patients and their mean age ranged from 49.5 - 67.2 years.	Survivors are defined as patients who have had a cancer diagnosis in the past, including those currently receiving active treatment, those in remission or cured and those who are in the terminal stages of disease.	A variety of online platforms were used including email, online educational resources, online support groups or message boards, cancer information websites and interactive websites.	Three interventions: (i) Linking patients to their treating team of clinicians<br>(ii) Connecting patients with each other<br>(iii) Educational resources.<br><br>Duration: 4 weeks to 12 months.	FACT-B, SF-12, EORTC QLQ-C30, EQ-5D, EPIC-26, 15DHRQoL, bespoke QoL measure	The overall benefit of online interventions for cancer patients is unclear.<br>Although there is significant promise, the few interventions that have been rigorously analysed demonstrate mixed efficacy, often of limited duration.
Mewes et al, 2012	To systematically review the evidence on the effectiveness of multidimensional rehabilitation programs for cancer survivors and to critically review the cost-effectiveness studies of cancer rehabilitation.	16 studies originated from 11 trials (11 RCTs, 3 pre-test-post-test, 1 quasi experimental, 1 longitudinal)	2,175 mixed cancer patients, predominantly breast. RCTs included from 21 to 199 participants. Age range not specified.	Patients with any type of cancer who finished primary treatment with an expected survival duration of at least 1 year. Hormone therapy could still be ongoing.	Face to face in an inpatient setting	Multidimensional rehabilitation defined as consisting of two or more rehabilitation interventions directed at the ICF dimensions. Interventions typically included exercise, CBT, psychotherapy, education and return to work interventions.<br>Programme duration: 4 to 15 weeks.	EORTC QLQ-C30, RAND-36, FACT-G, FACT-B, SF-12	Effect sizes for QoL were in the range of -0.12 (95% CI:-0.45 to -0.20) to 0.98 (95% CI, 0.69 to 1.29).<br><br>Multi and mono dimension interventions were equally effective.

For peer review only - http://bmjopen.bmj.com/site/about/guidelines.xhtml

Mishra et al, 2012	To evaluate the effectiveness of exercise on overall HRQoL and HRQoL domains among adult post-treatment cancer survivors.	40 trials / 38 RCTs	3,694 mixed cancer patients during and post-treatment were randomised. Over 50% included breast cancer patients only. Mean age range: 39 to 68 years	Participants who have completed treatment	Settings included a gym, community centre, yoga studio, or university or hospital facility. Home-based interventions were included.	Exercise was defined as physical activity causing an increase in energy expenditure in a systematic manner in terms of frequency, intensity, and duration. Included prescribed, active exercise formats of aerobic, resistance , stretching or aerobic/resistance combinations. Some interventions included modules in psychological or behavioural education. Duration ranged from 3 weeks to one year. Frequency varied between daily to once per week. Sessions lasted from 20 to more than 90 minutes.	EORTC QLQ-C30, FACT-G, FACT-B, FACT-F, FACT-An, FACT-Lym, FACIT-F, CARES-SF, QoL Index, SF-36, Neck Dissection Impairment Index for QoL for head and neck cancer survivors.	Exercise has a positive impact on QoL with improvements in global QoL.
Osborn et al, 2006	To investigate the effects of CBT and patient education (PE) on commonly reported problems (i.e. depression, anxiety, pain, physical functioning and quality of life) in adult cancer survivors.	15 RCTs	1,492 mixed cancer patients. Age range: 18-84 years.	Defined as beyond the time of diagnosis	In a group or individually, face to face	Interventions included; Group or Individual CBT, Patient Education. CBT intervention duration ranged from 3 – 55 weeks. Frequency varied from 1 hour per week to 2 hours per week. PE duration ranged from one 20-minute session to 6 weekly one-hour sessions.	FACT	QoL was improved at short-term and long - term follow up after CBT. Patient Education was not related to improved outcomes. Individual interventions were more effective than group.

For peer review only - http://bmjopen.bmj.com/site/about/guidelines.xhtml

For peer review only

Smits et al, 2015	To evaluate the effectiveness of lifestyle intervention in improving QoL of endometrial and ovarian cancer survivors.	8 studies / 3 RCTs	413 survivors of endometrial and ovarian cancer were included in the analysis. 153 survivors were included in the RCTs. Age range not specified.	Adults diagnosed with endometrial cancer having completed primary treatment (surgery, chemotherapy or radiotherapy)	Home-based, individually or group-based	Physical activity, behavioural change, nutritional, counselling interventions<br>The duration varied from 12 weeks to 12 months.	FACT-G, FACT-F, FACT-O, SF-36 and QLACS	The review did not show improvements in global QoL. The authors concluded that lifestyle interventions have the potential to improve QoL in this population.
Spark et al, 2013	To determine the proportion of physical activity and/or dietary intervention trials in breast cancer survivors that assessed post-intervention maintenance of outcomes, the proportion of trials that achieved successful post-intervention maintenance of outcomes, and the sample,	16 studies originated from 10 RCTs	1,536 breast cancer survivors during or after treatment. Age range not specified.	Not specified	Interventions included face to face contact, printed information and telephone counselling or home-based delivery	Interventions were described as physical activity and/or dietary behaviour change aiming to increase aerobic fitness, strength, physical activity.<br>Most interventions lasted 1-4 months, with some lasting longer than 6 months.	Measures not specified	More research is needed to identify the best ways of supporting survivors to make and maintain these lifestyle changes. QoL-specific outcomes from three studies not reported.

For peer review only - http://bmjopen.bmj.com/site/about/guidelines.xhtml

	intervention, and methodological characteristics common among trials that achieved successful post intervention maintenance of outcomes.							
Spence et al, 2009	To summarise the literature on the health effects of exercise during cancer rehabilitation and to evaluate the methodological rigor of studies in this area.	13 studies originated from 10 trials, 4 of which were RCTs	327 mixed cancer patients, mostly breast cancer patients. The sample size for the RCTs was 245 patients and their mean age ranged from 18 to 65 years.	Patients who had recently completed treatment and had reported no plans for additional treatment. 'Recently completed' was defined as having completed treatment no more than 12 months prior to enrolment.	Interventions were either supervised exercise programmes or home-based, unsupervised exercise programmes. One study employed exercise physiologists to prescribe individually-tailored exercise programmes.	Most interventions were aerobic or resistance-training exercise programmes. Most studies prescribed cycling or walking ergometers for the aerobic component. Studies incorporating resistance training prescribed either exercises using machines or resistance bands. Duration varied from 2 weeks to 14 weeks with a frequency of daily exercise to two or three sessions per week.	Cancer Rehabilitation Evaluation System	The findings from this review suggest that exercise can provide a variety of benefits for cancer survivors during the rehabilitation period, including an improved QoL.

For peer review only

Zachariae et al, 2015	To evaluate the effectiveness of expressive writing for improving psychological and physical health in cancer patients and survivors.	16 RCTs	1,797 cancer patients or survivors. Breast cancer, ovarian, renal, prostate, colorectal and mixed cancers. Age range not specified.	Not specified	Lab or home-based	Expressive writing interventions requiring participants to disclose their emotions in sessions. The duration of the intervention ranged from 3 – 4 sessions, which were daily, weekly or bi-weekly.	FACT-B, FACT-G, FACT-BMT, QLQ-C30	The review did not support the general effectiveness of expressive writing in cancer patients and survivors.
Zeng et al, 2014	To examine the effectiveness of exercise intervention on the quality of life of breast cancer survivors.	25 studies included in the qualitative synthesis, 19 studies included in meta-analysis. 22 RCTs	1,073 breast cancer patients aged 18 years or over.	Individuals who had completed active cancer treatment.	Face to face, by telephone	Interventions included any type of exercise - aerobic, resistance or combination of aerobic and resistance, yoga, tai chi, aerobic and strength training, aerobic and resistance training and stretching. The duration of the intervention ranged from 4 to 52 weeks. Time per session varied from 15 to 90 mins, 1 to 5 times per week.	Generic QoL measures: SF-36, FACT-G, EORTC-QLQ-C30. Cancer site-specific QoL measures: FACT-B, EORTC QLQ BR23.	The review found consistent positive effects of exercise interventions in overall QoL and certain QoL domains. There was a small to moderate effect of interventions on site-specific QoL. Single type of exercise intervention general aerobic, yoga or tai chi had significant differences in QoL score changes.

For peer review only - http://bmjopen.bmj.com/site/about/guidelines.xhtml

Abbreviations: QoL = Quality of Life; HRQoL = Health-related Quality of Life; 15DHRQoL = 15-dimensional health related quality of life; ABS = Affects balance scale; CARES = Cancer rehabilitation evaluation system; CARES-SF = Cancer Rehabilitation Evaluation System Short Form; EORTC QLQ-C30 = European Organisation for Research and Treatment of Cancer Quality of Life Questionnaire C30; EORTC QLQ-C33 = European Organization for Research and Treatment of Cancer Quality of Life Questionnaire C33; EPIC = Expanded Prostate Cancer Index Composite; EPIC-26 = Expanded Prostate Cancer Index Composite Short Form; EQ-5D, EuroQol-5D ''feeling thermometer"; FACIT-F = Functional Assessment of Chronic Illness—Fatigue; FACIT-Sp = Functional Assessment of Chronic Illness—Spiritual well-being; FACT-An = Functional Assessment of Cancer Therapy—Anaemia scale; FACT-B = Functional Assessment of Cancer Therapy—Breast Cancer; FACT-C = Functional Assessment of Cancer Therapy—Colorectal; FACT-F = Functional Assessment of Cancer Therapy—Fatigue; FACT-G: Functional Assessment of Cancer Therapy—General; FACT H&N = Functional Assessment of Cancer Therapy—Head & Neck; FACTIT = Functional Assessment of Chronic Illness Therapy; FACT-Lym = Functional Assessment of Cancer Therapy—Lymphoma; FACT-O = Functional Assessment of Cancer Therapy—Ovarian; FACT-P = Functional Assessment of Cancer Therapy—Prostate; FLIC= Functional Living Index for Cancer; GHQ = General health questionnaire; IFS-CA = Inventory of functional status—cancer; MCS/PCS = Mental Component Score/Physical Component Score; Modified Rotterdam QoL Survey; Neck Dissection Impairment Index for QoL for head and neck cancer survivors; NHP = Nottingham Health Profile; PCa-QoL = Prostate Cancer Quality of Life Instrument; QLACS = Quality of Life in Adult Cancer Survivors; QLI = Quality of life index; QLQ-PR25 = European Organisation for Research and Treatment of Cancer Quality of Life Questionnaire-Prostate Module; QoL-BC = quality of life questionnaire—breast cancer; RAND-36 = 36-Item Short Form Health Survey; SDS = Symptom Distress Scale; SF-12 = Medical Outcomes Study Short-Form Health Survey 12; SF-36 = Medical Outcomes Study Short-Form Health Survey 36; SIP = Sickness impact profile; UCLA-PCI = University of California, Los Angeles, Prostate Cancer Index; VAS = Visual analogue scale; WHOQOL-BREF = World Health Organization Quality of Life Assessment.

For peer review only - http://bmjopen.bmj.com/site/about/guidelines.xhtml

Table 4: Components of the interventions by study

	Cramer et al, 2012[23]	Fong et al, 2012[10]	Buffart et al, 2012[11]	Khan et al, 2012[8]	Mishra et al, 2012[12]	Culos-Reed et al, 2012[14]	Bourke et al, 2015[28]	Duijts et al, 2011[9]	Ferrer et al, 2011[19]	Fors et al, 2011[24]	Galveo et al, 2005[13]	Gerritsen and Vincent 2015[20]	Huang et al, 2015[27]	McAlpine et al, 2015[15]	Mewes et al, 2012[18]	Osborn et al, 2006[17]	Smits et al, 2015[21]	Spark et al, 2013[25]	Spence et al, 2009[16]	Zachariae et al, 2015[22]	Zeng et al. 2014[26]
**PHYSICAL**																					
Aerobic		●			●		●	●	●		●						●		●		●
Aerobic and Resistance					●		●														●
Resistance					●		●				●										●
Aquatic exercise					●																
Cardiovascular programme											●										●
Cycling					●						●	●							●		
Dance movement								●													
Enhanced Standard Care							●														
Exercise not specified				●											●		●	●			
Expressive writing																				●	
METs targeted									●												
Dietary intervention				●			●										●	●			
Pilates					●																
Resistance/strength training		●			●			●			●	●					●		●		●
Running					●																
Self-management exercise								●													
Stretching/Flexibility exercises											●								●		●
Swimming												●									
Tai Chi					●																●
Treadmill																			●		
Walking					●			●			●	●							●		
Weight training								●													
Yoga/meditation	●		●		●	●		●													●
Qigong					●																
**PSYCHOLOGICAL, EDUCATIONAL & BEHAVIOURAL**																					
Body mind								●													
Cognitive behavioural							●	●													

For peer review only - http://bmjopen.bmj.com/site/about/guidelines.xhtml

	Cramer et al, 2012[23]	Fong et al, 2012[10]	Buffart et al, 2012[11]	Khan et al, 2012[8]	Mishra et al, 2012[12]	Culos-Reed et al, 2012[14]	Bourke et al, 2015[28]	Duijts et al, 2011[9]	Ferrer et al, 2011[19]	Fors et al, 2011[24]	Galveo et al, 2005[13]	Gerritsen and Vincent 2015[20]	Huang et al, 2015[27]	McAlpine et al, 2015[15]	Mewes et al, 2012[18]	Osborn et al, 2006[17]	Smits et al, 2015[21]	Spark et al, 2013[25]	Spence et al, 2009[16]	Zachariae et al, 2015[22]	Zeng et al. 2014[26]
stress therapy																					
Cognitive behavioural therapy								•		•					•	•	•				
Cognitive G therapy								•													
Combined psychosexual								•													
Comprehensive coping strategy								•													
Coping skills																					
Emotional support								•		•											
Group therapy							•	•								•	•				
Guided imagery								•													
Image consultant				•																	
Mindfulness based stress reduction programme													•								
Motivational interviewing																					
Problem solving training								•													
Progressive relaxation training																					
Psychotherapy															•						
Psychosocial therapy								•													
Return to work interventions															•						
Social support								•		•											
Stress management								•													
Health education								•							•	•					
Psychological education				•			•	•		•					•	•					
Peer support				•				•						•							
**MODE OF DELIVERY**																					
CD/manuals/videos			•				•			•											
Face to face				•	•		•	•									•	•			
Home based			•	•	•			•									•	•	•		
Inpatient setting				•											•						
Multidisciplinary rehabilitation programme				•			•								•						

For peer review only

	Cramer et al, 2012[23]	Fong et al, 2012[10]	Buffart et al, 2012[11]	Khan et al, 2012[8]	Mishra et al, 2012[12]	Culos-Reed et al, 2012[14]	Bourke et al, 2015[28]	Duijts et al, 2011[9]	Ferrer et al, 2011[19]	Fors et al, 2011[24]	Galveo et al, 2005[13]	Gerritsen and Vincent 2015[20]	Huang et al, 2015[27]	McAlpine et al, 2015[15]	Mewes et al, 2012[18]	Osborn et al, 2006[17]	Smits et al, 2015[21]	Spark et al, 2013[25]	Spence et al, 2009[16]	Zachariae et al, 2015[22]	Zeng et al. 2014[26]
Printed information			•															•			
Support from nurse or voluntary organisations							•	•		•											
Telephone	•		•					•										•			•
Web based							•	•		•				•							

For peer review only

For peer review only - http://bmjopen.bmj.com/site/about/guidelines.xhtml

Table 5: Reported effect size from meta-analyses in reviews

Authors	Intervention	Type of effect size reported	Reported effect size	Overall finding
Buffart et al, 2012◊	Yoga	SMD (7 studies)		
		General QoL	0.37,0.11-0.62	+
Cramer et al, 2012◊	Yoga	SMD (4 studies) Global QoL	0.62, 0.04 to 1.21;	+
Ferrer et al, 2011 * $	Exercise	SMD (78 studies)		
		All intervention groups (Immediate FU)	0.34, 0.24 to 0.43	+
		Intervention vs control, adjusted for baseline differences	0.24, 0.12 to 0.35	+
		Delayed FU		
		All intervention groups	0.42, 0.23 to 0.61	+
		Intervention vs Control adjusted for baseline	0.20, -0.058 to 0.46	+
Fong et al, 2012	Exercise	2 studies	3.4, 0.4 to 6.4	+
		9 studies	22.1, 16.8 to 27.4	+
Gerritsen and Vincent 2015	Exercise	SMD: intervention vs control	5.55, 3.19 to 7.9	+
Mishra et al, 2012 ◊	Exercise	SMD: baseline to after intervention (11 studies)	0.48, 0.16 to 0.81	+
		3-6 month follow up (181 participants)	0.14, -0.38 to 0.66	-
		6 month follow up (115 participants) (2 studies)	0.46, 0.09 to 0.84	+
Zeng et al, 2014	Exercise	Standardised Mean Difference (0verall) (6 studies)	0.70, 0.21, 1.19	+
		Cancer specific (10 studies)	0.38, 0.03 to 0.74	+
Duijts et al, 2011	Exercise	SMD (or Hedges g for small sample size, with adjustment) (27 studies)	0.298, 0.117 to 0.479, p = 0.001	+

For peer review only

For peer review only - http://bmjopen.bmj.com/site/about/guidelines.xhtml

	Behavioural intervention		0.045, -0.044 to 0.135, p=0.322	uncertain
Osborn et al, 2006	CBT	SMD Overall (11 studies)	0.91, 0.38 to 1.44, p<0.01	+
		Short term (<8wks)	1.45, 0.43 to 2.47	+
		Long term (>8wks)	0.26, 0.06 to 0.46	+
		Individual CBT (7 studies)	0.95, -0.367 to 1.536	+
		Individual vs Group CBT (1 study)	0.37, -0.02 to 0.75,	uncertain
	Patient Education	(1 study)	-0.04, -0.38 to 0.29,	-
Smits et al, 2015	Lifestyle interventions	SMD		+
		3 months	1.16, -5.91 to 8.23,	
		6 months	2.48, -4.63 to 9.58,	
Zachariae et al, 2015	Expressive writing	Hedges's *g*	0.09, -0.5 to 0.24,	+

* random effects assumption
◊ Reviews rated as high quality
$ findings sustained for random or fixed effects, random effects reported.

For peer review only

Overall Effectiveness of Interventions: meta-analysis findings

Meta-analyses were reported in 11 reviews and the effect sizes (as reported in the original reviews) are tabulated (Table 5). Of six publications providing meta-analyses of physical activity (not including yoga), all found convincing positive associations for studies testing response between 1 and 26 weeks post-treatment. Long term effects were not tested by all, although Fong and Zeng did show persistent effects at six months and a year respectively. [10 26] One review [19] showed uncertain outcomes at 3-6 months, although shorter and longer term outcomes were favourable. This review showed equivocal effects when the intervention group was compared with the control group, once adjusted for quality of life and covariates at baseline. The two meta-analyses of yoga interventions showed positive effects, [11 23] as did a review of CBT. [17] There was no evidence of benefit in quality of life following patient education [17] and behavioural interventions.[9]

For peer review only - http://bmjopen.bmj.com/site/about/guidelines.xhtml

Two reviews reported effect sizes from individual studies but did not undertake meta-analyses.[18 24] Mewes's review of multidimensional rehabilitation included ten studies, nine of which had global quality of life outcomes; of these, seven showed benefit with effect sizes ranging from 0.04 to 0.99 (no confidence intervals reported). [18] Fors's review included six RCTs only four of which included a quality of life measure; [24] two of these showed positive effect sizes (ranging from 0.56, 95%CI: 0.09 to 1.03; 95% CI: 0.63, 0.11 to 1.18); one showed improved and one a worsening of quality of life as a non-standardised mean score. Five reviews [8 13 15 16 25] did not report meta-analyses or effect sizes; mostly these provided mean change scores or narrative statements. On the whole these gave a mixed picture, often resorting to sub-group analysis by cancer type or different dimensions of quality of life.

Physical Activity: Summary Findings

Cramer's[23] high quality review of 6-12 weeks of yoga in breast cancer patients showed a large increase in general quality of life, a finding that was consistent with reviews by Buffart [11] and Culos-Reed, which scored lower on the AMSTAR. [14] Mishra's [12] high quality review of people with multiple cancers, 50% of whom had breast cancer, found that physical activity had a positive effect on global quality of life at three and six months follow up, as did Smits's high quality review of endometrial cancer and Gerritsen's moderate quality review of mixed cancers.[20 21] Fong's[10] high quality review of breast cancer, colorectal, endometrial and mixed cancers, similarly found physical interventions improved general quality of life on average at 13 weeks follow up (range 3-60 weeks). Bourke's review of prostate cancer found personalised lifestyle interventions helpful[28], and McAlpine's review of mixed cancers including prostate found benefit of activity following medication treatment.[15]

There was inconsistency across the reviews with regard to the types of exercise interventions that were most effective. Fong [10] found aerobic plus resistance training to be significantly more effective

For peer review only - http://bmjopen.bmj.com/site/about/guidelines.xhtml

than aerobic training alone on many aspects of quality of life. However, Zeng's [26] moderate quality review suggested that single types of exercise interventions (general aerobic, yoga or tai chi) were more effective at increasing quality of life at 4-52 weeks after intervention; half of the studies assessed interventions between 8-12 weeks. Duijts's [9] study of breast cancer patients found only small effects of physical activity on quality of life (at 8 -26 weeks after intervention); and Spence's [16] study of mixed but mostly breast cancer patients reported evidence that physical activity improved overall quality of life but only four of ten trials maintained the intervention and only a fifth of trials seemed to assess outcome at 3 months and beyond. Zeng's [26] review of breast cancer patients found small but positive benefits of physical activity on overall quality of life. Galvão's [13] review of mixed cancers gave preliminary evidence of positive benefits on a Modified Rotterdam QoL measure, but no overall effects were reported. However, Spark's [25] review of breast cancer patients showed that the impact of physical activity on quality of life was not convincing. Although Spark did not report effect sizes, two of the studies in that review included quality of life measures, both of which reported effect sizes in the original papers: one showed positive benefits on FACT-G and FACT-B at 8 months (effect sizes 9.8 to 13.4), but not at 24 months follow up; the other showed no significant effects on FACT-G overall, but when the cancer specific FACT-G was assessed at six month follow up, there was benefit (4.9, 0.2 to 9.6). Ferrer's [19] study of breast, prostate, endometrial, head and neck, ovarian cancers and lymphoma found small but positive effects of exercise at long term follow up on multiple measures of quality of life. The efficacy of the interventions appeared greater with shorter duration treatments, and if exercise was supervised. Aerobic intensity predicted improvements in quality of life.

Psychological and behavioural interventions: Summary Findings

Only one of the reviews of psychological and behavioural interventions was classified as high quality: Huang's [27] meta-analysis of breast cancer patients showed that mindfulness-based stress reduction

For peer review only - http://bmjopen.bmj.com/site/about/guidelines.xhtml

programmes had a significant effect in improving overall quality of life. Duijts's [9] review, on the other hand, concluded that behavioural techniques such as problem solving, stress management and CBT did not significantly improve health-related quality of life. Nevertheless, Fors's [24] review of breast cancer patients showed CBT improved quality of life. No meta-analysis or overall effect sizes were reported due to heterogeneity. Further support for CBT came from Osborn's [17] review of group and individually delivered CBT for mixed cancers; individual interventions were more effective than group-based treatment. CBT showed both short-term [24] and long-term improvements in quality of life.[17] Five primary papers in one review assessed the effect of social and emotional support as an intervention, four of them finding no effect, and one reporting a significant improvement in quality of life on one measure.[24] There was no evidence that psychosocial education increased quality of life.[17 24]

Multidimensional and Multidisciplinary rehabilitation

Khan's [8] high quality review of breast cancer patients included just two studies, only one of which provided low level evidence that multidisciplinary rehabilitation improved participation and social activities. The other showed no significant effects. Mewes's [18] moderate quality review of breast and other cancers treated by inpatient multidisciplinary rehabilitation demonstrated no differences between multidimensional and single dimension interventions, with benefits of both on physical outcomes. Bourke's review of prostate cancer survivors examined the effectiveness of multidisciplinary approaches based on findings from three primary studies.[28] They concluded that such interventions showed small benefits for quality of life, typically when they involved a smaller number of health professionals, thus allowing more focused tailoring of the interventions.

For peer review only - http://bmjopen.bmj.com/site/about/guidelines.xhtml

Intervention modality

The effectiveness of online educational interventions was unclear. McAlpine's [15] review of lung, prostate, head and neck and a smaller number of mixed cancers showed equivocal findings. There were benefits to online education, message boards, but mixed effects for interactive websites, and worse outcomes from one study on email interventions. One interesting review was of expressive writing interventions, but this found no benefit on quality of life, although small effects would be undetected. [22] Individuals with low levels of emotional support appeared to benefit more than others.

Adverse Effects

Five reviews[11 12 15 23 26] included reports of adverse events. Of four studies in Buffart's[11] review, one reported back spasm in a yoga class in a patient with a history of back problems. In Cramer's[23] review of three studies reporting adverse events, there was one adverse event (back spasm) in 138 patients. McAlpine's[15] review included two studies that reported adverse effects of online support groups. One of these reported transient helplessness, anxiety, confusion and depression at six months; whilst the other showed poorer quality of life despite high levels of reported satisfaction. Zeng's[26] review of 25 trials found one study with reports of exercise related lymphedema. In Mishra's[12] review, six studies reported adverse effects including lymphoedema, gynaecological complications and influenza in the exercise group. One study reported back, knee and hip problems. Three participants in one study reported thrombosis and infection following exercise interventions. Another study found hip pain, sciatica, arm discomfort (n = 4), knee discomfort (n = 10), ankle discomfort (n = 3), and foot discomfort (n = 8) with asymptomatic ischaemia and conduction problems on ECG. A further study reported lung metastases, pulmonary embolism and palpitations. Another study reported soft tissue injury following exercise, and cholecystitis following stroke.

For peer review only - http://bmjopen.bmj.com/site/about/guidelines.xhtml

Cancer recurrence, although not a direct effect of interventions, was common and another reason to stop participation in the research.

DISCUSSION

Main findings

21 reviews were included and showed a lack of definitive and consistent evidence across 465 primary studies of which 362 were RCTs. In part this is explained by substantial variation in study designs and outcome measures used to indicate quality of life. All systematic reviews of physical activity demonstrated improved overall quality of life, but few studies assessed long-term outcomes beyond 3 months, and even fewer assessed outcomes beyond a year after the intervention. More focused research and a consistent approach are required to explore the effect on the subdomains of quality of life.[12] A higher quality review suggests that aerobic plus resistance training provide maximum improvements in quality of life.[10] There was more evidence of physical rather than psychological or other types of interventions.

One of the included reviews for psychological or behavioural interventions was of high quality.[27] CBT is effective for improving quality of life in the short and long term,[17 24] especially when provided as an individual intervention.[17] There is not much evidence to support comparative effectiveness of intervention modalities such as group versus individual, mono-dimensional versus multi-dimensional or multidisciplinary; further work is needed to examine these different approaches. Given the accessibility of social media and its popularity, the findings that email contact was related to poorer quality of life need further investigation; although interactive websites were beneficial, the overall findings about digital interventions were equivocal.

For peer review only - http://bmjopen.bmj.com/site/about/guidelines.xhtml

Limitations

The current review has some limitations in the methodology. Studies not in English and grey literature were not included due to time constraints as the review was undertaken as a part of a programme development grant to inform the design of a future research programme application.

We encountered some methodological limitations in included reviews. Some used multiple outcomes and often had a very broad understanding of QoL and used diverse measures of quality of life. There was no consistent reporting standard.

We did not consider outcomes such as wellbeing or the multiple sub-domains of quality of life to avoid the risk of generating findings due to multiple testing in smaller sub-samples in underpowered analyses. Some reviews included few primary papers. We examined the sample sizes of RCTs included in reviews and whether there seemed to be any relationship with AMSTAR ratings. We found no obvious relationship, given AMSTAR scores refer to review quality rather than the quality of or sample size of individual RCTs. A review of primary RCTS might help to better understand and report robust findings from RCTs with large and adequate sample sizes, findings, which may otherwise be less visible in a review of reviews.

We found little overlap between reviews (tabulation available on request), reflecting their specific inclusion and exclusion criteria and interest in very specific interventions and cancer types. We did not evaluate the methodological quality or bias of the original studies within each systematic review. Ten reviews planned to assess publication bias; three of these could not perform any specific tests of bias due to small samples.[8 23 27] Consequently seven studies tested for publication bias.[9 10 12 17 19 20 22] Three of these reported that publication bias was not significant.[10 20 22] Four reviews [9 12 17 19] reported significant publication bias suggesting caution in assuming there is definitive evidence for exercise and CBT.

For peer review only - http://bmjopen.bmj.com/site/about/guidelines.xhtml

The physical and psychosocial concerns of patients at different time periods of the cancer experience will vary greatly and interventions effective at one stage may not be suitable for another. Most reviews defined 'survivors' as those who had completed active treatment before the onset of the study. [10 13 14 16 18 19 23 24 26] Some specified a time frame, from immediately after surgery to 15 years after active treatment. [12] One review defined survival as being from diagnosis onwards. [17] Another included terminal stages of cancer. [15] The majority of the reviews incorporated studies combining patients during and post treatment. [9 11-15 23-25] These differing definitions of living with and beyond cancer make comparison difficult, and a standardised approach to trials and reporting of studies is needed.

Interventions were offered to patients based on their diagnosis of cancer, rather than low quality of life, which may have led to underestimation of potential beneficial effects. Future research should consider the effectiveness of interventions targeting people living beyond all types of cancer, and with poor overall quality of life.

Conclusions

Systematic reviews of cancer patients and their QoL showed that effective interventions included physical activity, CBT and mindfulness-based stress reduction training. Personalised lifestyle interventions showed promise, as did social and emotional support. Educational and information provision appear ineffective, and there were few studies of electronic interventions. Currently, there is no standard study design, outcome selection, or reporting convention adopted across these reviews. No single intervention can be recommended to those patients with a poor quality of life following cancer treatment as interventions were not targeting poorer quality of life, but cancer survivors in general.

For peer review only - http://bmjopen.bmj.com/site/about/guidelines.xhtml

Acknowledgements, competing interests, funding and all other required statements

This review was funded by NIHR-Programme Development Grant: RP-DG-1212-10014

The authors declare that they do not have any conflicts of interest.

The lead and corresponding authors (guarantors) affirm that the manuscript is an honest, accurate, and transparent account of the study being reported; that no important aspects of the study have been omitted; and that any discrepancies from the study as planned (and, if relevant, registered) have been explained. All authors had access to the full data set, and the work was undertaken independently of the funders and sponsor.

Contributor Statement:

KB as PI for the review, designed the review, and prepared the review section for the original grant application, which overall was led by PDW. Input on design was provided by all authors (KB, EM, EH MD, JD, RR, LJ, LB, AM, TC, MT, ST, AK, PW) and PPI experts (Miriam Harris, Adrienne Morgan, and Louisa Smalley) in steering groups during preparation of the funding application and throughout the project; more specific additional input to design was provided by PW, SC. MD and JD were research fellows employed on the grant, and collected the papers, ran the searches and performed the first extraction under supervision by KB. MD and JD undertook the preliminary charting and extraction. EH (a PPI expert) and EM conducted the AMSTAR ratings, the final data extraction and edited the draft, under the supervision of KB. KB reviewed all data and checked and completed extraction of the data and identified relevant effect estimates, and led on writing the paper, edited consecutive drafts of the MS, and the produced the final draft. All authors (KB, EM, EH, MD, JD, RR, LJ, LB, AM, TC, MT, ST, AK, PW) contributed to the reviewing consecutive drafts of the paper for content, the presentation, and discussion about the findings and interpretation at each stage of the review process, as well as the structure of the paper. All authors (KB, EM, EH, MD, JD, RR, LJ, LB, AM, TC, MT, ST, AK, PW) commented on and approved the final version. We thank Miriam Harris, Adrienne Morgan, and Louisa Smalley for helpful analysis and comments in the design, planning and delivery of the research including this review, and in the construction of SURECAN dissemination plans and the design of a future trial. MD and EM are joint first authors given their respective contributions to the paper, both are agreed on this, as is KB.

The Corresponding Author has the right to grant on behalf of all authors and does grant, on behalf of all authors, a worldwide licence to the Publishers and its licensees in perpetuity, in all forms, formats and media (whether known now or created in the future), to i) publish, reproduce, distribute, display and store the Contribution, ii) translate the Contribution into other languages, create adaptations, reprints, include within collections and create summaries, extracts and/or, abstracts of the Contribution, iii) create any other derivative work(s) based on the Contribution, iv) to exploit all subsidiary rights in the Contribution, v) the inclusion of electronic links from the Contribution to

third party material where-ever it may be located; and, vi) licence any third party to do any or all of the above.

Data Sharing Statement

No additional data are available.

Annex1: Full search strategy (supplementary file)

For peer review only

For peer review only - http://bmjopen.bmj.com/site/about/guidelines.xhtml

REFERENCES

1. Cancer Research UK C. Cancer Survival Statistics 2015 [Available from: http://www.cancerresearchuk.org/health-professional/cancer-statistics/survival.
2. Jarrett N, Scott I, Addington-Hall J, et al. Informing future research priorities into the psychological and social problems faced by cancer survivors: A rapid review and synthesis of the literature. *European Journal of Oncology Nursing* 2013;17(5):510-20. doi: http://dx.doi.org/10.1016/j.ejon.2013.03.003
3. Miller KD, Triano LR. Medical issues in cancer survivors--a review. *Cancer J* 2008;14(6):375-87. doi: 10.1097/PPO.0b013e31818ee3dc [published Online First: 2008/12/09]
4. MacmillanCancerSupport. Worried Sick: the emotional impact of cancer http://www.macmillan.org.uk/documents/getinvolved/campaigns/campaigns/impact_of_cancer_english.pdf: Macmillan Cancer Support 89 Albert Embankment London SE1 7UQ; 2006 [
5. NHS Choices. Complementary and alternative medicine [Available from: http://www.nhs.uk/Livewell/complementary-alternative-medicine/Pages/complementary-and-alternative-medicine.aspx accessed October 2016.
6. Shea BJ, Grimshaw JM, Wells GA, et al. Development of AMSTAR: a measurement tool to assess the methodological quality of systematic reviews. *BMC Med Res Methodol* 2007;7:10. doi: 10.1186/1471-2288-7-10 [published Online First: 2007/02/17]
7. Mays N, Pope C, Popay J. Systematically reviewing qualitative and quantitative evidence to inform management and policy-making in the health field. *Journal of health services research & policy* 2005;10 Suppl 1:6-20. doi: 10.1258/1355819054308576 [published Online First: 2005/08/02]
8. Khan F, Amatya B, Ng L, et al. Multidisciplinary rehabilitation for follow-up of women treated for breast cancer. *Cochrane Database Syst Rev* 2012;12:Cd009553. doi: 10.1002/14651858.CD009553.pub2 [published Online First: 2012/12/14]
9. Duijts SF, Faber MM, Oldenburg HS, et al. Effectiveness of behavioral techniques and physical exercise on psychosocial functioning and health-related quality of life in breast cancer patients and survivors--a meta-analysis. *Psychooncology* 2011;20(2):115-26. doi: 10.1002/pon.1728 [published Online First: 2010/03/26]
10. Fong DYT, Ho JWC, Hui BPH, et al. Physical activity for cancer survivors: meta-analysis of randomised controlled trials. *BMJ (Clinical research ed)* 2012;344 doi: 10.1136/bmj.e70
11. Buffart LM, van Uffelen JG, Riphagen, II, et al. Physical and psychosocial benefits of yoga in cancer patients and survivors, a systematic review and meta-analysis of randomized controlled trials. *BMC Cancer* 2012;12:559. doi: 10.1186/1471-2407-12-559 [published Online First: 2012/11/28]
12. Mishra SI, Scherer RW, Geigle PM, et al. Exercise interventions on health-related quality of life for cancer survivors. *Cochrane Database of Systematic Reviews* 2012(8) doi: 10.1002/14651858.CD007566.pub2
13. Galvão DA, Newton RU. Review of exercise intervention studies in cancer patients. *Journal of clinical oncology : official journal of the American Society of Clinical Oncology* 2005;23(4):899-909. doi: 10.1200/jco.2005.06.085 [published Online First: 2005/02/01]
14. Culos-Reed SN, Mackenzie MJ, Sohl SJ, et al. Yoga & cancer interventions: a review of the clinical significance of patient reported outcomes for cancer survivors. *Evidence-based complementary and alternative medicine : eCAM* 2012;2012:642576. doi: 10.1155/2012/642576 [published Online First: 2012/11/06]
15. McAlpine H, Joubert L, Martin-Sanchez F, et al. A systematic review of types and efficacy of online interventions for cancer patients. *Patient Education and Counseling* 2015;98(3):283-95.

For peer review only - http://bmjopen.bmj.com/site/about/guidelines.xhtml

16. Spence RR, Heesch KC, Brown WJ. Exercise and cancer rehabilitation: a systematic review. *Cancer Treat Rev* 2010;36(2):185-94. doi: 10.1016/j.ctrv.2009.11.003 [published Online First: 2009/12/08]
17. Osborn RL, Demoncada AC, Feuerstein M. Psychosocial interventions for depression, anxiety, and quality of life in cancer survivors: meta-analyses. *Int J Psychiatry Med* 2006;36(1):13-34. [published Online First: 2006/08/25]
18. Mewes JC, Steuten LMG, M.J IJ, et al. Effectiveness of multidimensional cancer survivor rehabilitation and cost-effectiveness of cancer rehabilitation in general: A systematic review. *Oncologist* 2012;17(12):1581-93.
19. Ferrer RA, Huedo-Medina TB, Johnson BT, et al. Exercise interventions for cancer survivors: a meta-analysis of quality of life outcomes. *Annals of behavioral medicine : a publication of the Society of Behavioral Medicine* 2011;41(1):32-47. doi: 10.1007/s12160-010-9225-1 [published Online First: 2010/10/12]
20. Gerritsen JK, Vincent AJ. Exercise improves quality of life in patients with cancer: a systematic review and meta-analysis of randomised controlled trials. *British journal of sports medicine* 2016;50(13):796-803. doi: 10.1136/bjsports-2015-094787 [published Online First: 2016/01/01]
21. Smits A, Lopes A, Das N, et al. The effect of lifestyle interventions on the quality of life of gynaecological cancer survivors: A systematic review and meta-analysis. *Gynecol Oncol* 2015;139(3):546-52. doi: 10.1016/j.ygyno.2015.10.002 [published Online First: 2015/10/07]
22. Zachariae R, O'Toole MS. The effect of expressive writing intervention on psychological and physical health outcomes in cancer patients—a systematic review and meta-analysis. *Psycho-Oncology* 2015;24(11):1349-59. doi: 10.1002/pon.3802
23. Cramer H, Lange S, Klose P, et al. Yoga for breast cancer patients and survivors: a systematic review and meta-analysis. *BMC Cancer* 2012;12:412. doi: 10.1186/1471-2407-12-412 [published Online First: 2012/09/20]
24. Fors EA, Bertheussen GF, Thune I, et al. Psychosocial interventions as part of breast cancer rehabilitation programs? Results from a systematic review. *Psychooncology* 2011;20(9):909-18. doi: 10.1002/pon.1844 [published Online First: 2010/09/08]
25. Spark LC, Reeves MM, Fjeldsoe BS, et al. Physical activity and/or dietary interventions in breast cancer survivors: a systematic review of the maintenance of outcomes. *Journal of cancer survivorship : research and practice* 2013;7(1):74-82. doi: 10.1007/s11764-012-0246-6 [published Online First: 2012/11/28]
26. Zeng Y, Huang M, Cheng AS, et al. Meta-analysis of the effects of exercise intervention on quality of life in breast cancer survivors. *Breast cancer (Tokyo, Japan)* 2014;21(3):262-74. doi: 10.1007/s12282-014-0521-7 [published Online First: 2014/02/27]
27. Huang HP, He M, Wang HY, et al. A meta-analysis of the benefits of mindfulness-based stress reduction (MBSR) on psychological function among breast cancer (BC) survivors. *Breast cancer (Tokyo, Japan)* 2016;23(4):568-76. doi: 10.1007/s12282-015-0604-0 [published Online First: 2015/03/31]
28. Bourke L, Boorjian SA, Briganti A, et al. Survivorship and improving quality of life in men with prostate cancer. *Eur Urol* 2015;68(3):374-83. doi: 10.1016/j.eururo.2015.04.023 [published Online First: 2015/05/06]

For peer review only - http://bmjopen.bmj.com/site/about/guidelines.xhtml

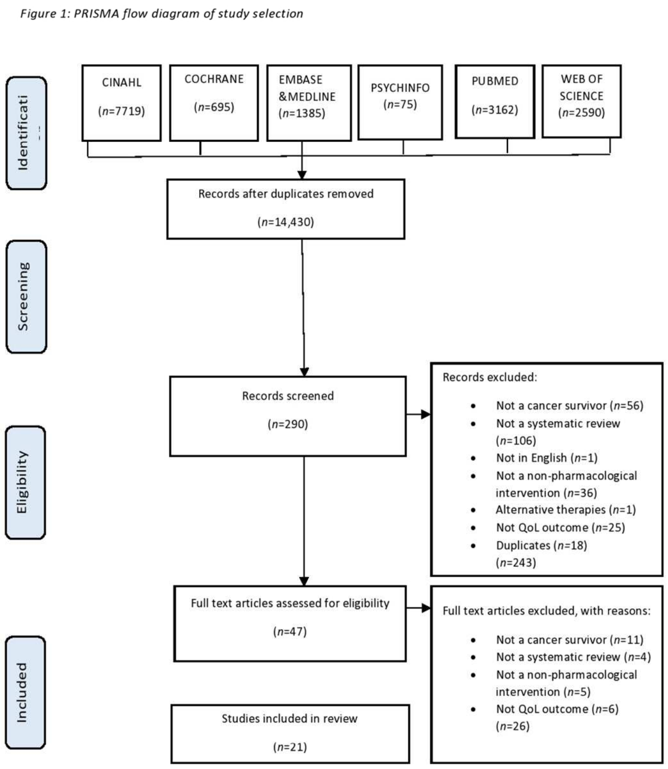

Figure 1: PRISMA Flowchart

104x148mm (300 x 300 DPI)

ONLINE SUPPLMENT: Excluded papers from the full paper search

Author	Date	Reference	Reason for exclusion
Kirshbaum et al.	2007	Kirshbaum MN. A review of the benefits of whole body exercise during and after treatment for breast cancer. *Journal of clinical nursing*. 2007.	This review was excluded as it was not a systematic review.
Badr et al.	2013	Badr H, Krebs P. A systematic review and meta-analysis of psychosocial interventions for couples coping with cancer. *Psycho-Oncology.* 2013.	This review was excluded as it was not cancer survivors, who have completed active treatment.
Jones et al.	2006	Jones LW, Demark-Wahnefried W. Diet, exercise, and complementary therapies after primary treatment for cancer. *The lancet oncology.* 2006.	This review was excluded as it was not systematic.
Burden et al.	2014	Burden S, Gibson DJ, Todd C, Gratton EK, Pilling M, Lal S. Dietary interventions for adult cancer survivors. *The Cochrane Library.* 2014.	This review was excluded as it was a protocol paper. We emailed the authors however, we were unable to find paper.
Keesing et al.	2015	Martin TA, Moran-Kelly RM, Roberts LM, Powe JG, Farrell SN, Singleton J. Effectiveness of individualized survivorship care plans on quality of life of adult female breast cancer survivors: a systematic review (provisional abstract). *JBI Database of systematic reviews and implementation reports.* 2015.	This paper was not a non-pharmacological psychosocial intervention.
Luckett et al.	2011	Luckett T, Britton B, Clover K, Rankin NM. Evidence for interventions to improve psychological outcomes in people with head and neck cancer: a systematic review of the literature. *Supportive care in cancer*. 2011.	This review was excluded as it was not cancer survivors, who have completed active treatment.
Friendenreich and Courneya	1996	Friedenreich CM, Courneya KS. Exercise as rehabilitation for cancer patients (structured abstract). *Clinical Journal of Sport Medicine*. 1996.	This review was excluded as it was a structured abstract only. We emailed the authors however, we were unable to find paper
Cheng et al.	2014	Cheng KK, Lim YT, Koh ZM, Tam WW. Home-based multidimensional survivorship programmes for breast cancer survivors. *The Cochrane Library.* 2014.	This review was excluded as it was a protocol paper. We emailed the authors however, we were unable to find paper.

For peer review only - http://bmjopen.bmj.com/site/about/guidelines.xhtml

de Boer et al.	2011	De Boer AG, Taskila T, Tamminga S, Frings-Dresen M, Feuerstein M, Verbeek J. Interventions to en-hance return-to-work for cancer patients. *Cochrane Database Syst Rev.* 2011.	This paper was excluded as QOL was not main outcome and it was not cancer survivors who had completed active treatment.
Solloway et al.	2016	Yan JH, Pan L, Zhang XM, Sun CX, Cui GH. Lack of efficacy of Tai Chi in improving quality of life in breast cancer survivors: a systematic review and meta-analysis (provisional abstract). *Asian Pacific journal of cancer prevention: APJCP*. 2014.	This review was excluded as it was a provisional abstract only. We emailed the authors however, we were unable to find paper
Ledesma et al.	2009	Ledesma D, Kumano H. Mindfulness-based stress reduction and cancer: a meta-analysis. *Psycho-Oncology.* 2009.	This review was excluded as it was not cancer survivors, who have completed active treatment.
Scott et al.	2013	Scott DA, Mills M, Black A, Cantwell M, Campbell A, Cardwell CR, Porter S, Donnelly M. Multidimensional rehabilitation programmes for adult cancer survivors. *The Cochrane Library.* 2013.	This paper was excluded as QOL was not main outcome
Khan et al.	2013	Khan F, Amatya B, Ng L, Drummond K, Olver J. Multidisciplinary rehabilitation after primary brain tumour treatment. *Cochrane Database syst rev.* 2013.	This review was excluded as it was not cancer survivors, who have completed active treatment.
Budhrani et al.	2014	Budhrani P. Optimal Timing of Mindfulness-Based Stress Reduction in Cancer: Research Synthesis and State of the Science. *The Journal of Alternative and Complementary Medicine.* 2014.	We were unable to access the full paper. Authors confirmed this was a presentation rather than a paper.
Oldervoll et al.	2004	Oldervoll LM, Kaasa S, Hjermstad MJ, Lund JÅ, Loge JH. Physical exercise results in the improved subjective well-being of a few or is effective rehabilitation for all cancer patients? (provisional abstract). *European Journal of Cancer.* 2004.	This review was excluded as it was not cancer survivors, who have completed active treatment. It included only two primary papers with cancer survivors, and not analysed separately.
Casellas-Grau et al.	2014	Casellas-Grau A, Font A, Vives J. Positive psychology interventions in breast cancer. A systematic review. *Psycho-Oncology*. 2014.	This review was excluded as it was not cancer survivors, who have completed active treatment.

For peer review only

Hulbert-Williams et al.	2015	Hulbert-Williams NJ, Storey L, Wilson KG. Psychological interventions for patients with cancer: psychological flexibility and the potential utility of Acceptance and Commitment Therapy. *European journal of cancer care.* 2015.	This review was excluded as it was not cancer survivors, who have completed active treatment.
Harder et al.	2012	Harder H, Parlour L, Jenkins V. Randomised controlled trials of yoga interventions for women with breast cancer: a systematic literature review. *Supportive care in cancer.* 2012.	This review was excluded as it was not cancer survivors, who have completed active treatment.
De Backer et al.	2009	De Backer IC, Schep G, Backx FJ, Vreugdenhil G, Kuipers H. Resistance training in cancer survivors: a systematic review (provisional abstract). *International journal of sports medicine.* 2009.	This paper was excluded as QOL was not main outcome.
Archer et al.	2015	Archer S, Buxton S, Sheffield D. The effect of creative psychological interventions on psychological outcomes for adult cancer patients: a systematic review of randomised controlled trials. *Psycho-Oncology.* 2015.	This review was excluded as it was not cancer survivors, who have completed active treatment.
Piet et al.	2012	Piet J, Würtzen H, Zachariae R. The effect of mindfulness-based therapy on symptoms of anxiety and depression in adult cancer patients and survivors: A systematic review and meta-analysis. *Journal of Consulting and Clinical Psychology.* 2012.	This paper was excluded as QOL was not main outcome.
Cramp et al.	2010	Cramp F, James A, Lambert J. The effects of resistance training on quality of life in cancer: a systematic literature review and meta-analysis. *Supportive care in cancer*. 2010.	This review was excluded as it was not cancer survivors, who have completed active treatment.
Stan et al.	2012	Stan DL, Collins NM, Olsen MM, Croghan I, Pruthi S. The evolution of mindfulness-based physical interventions in breast cancer survivors. *Evidence-Based Complementary and Alternative Medicine*. 2012.	This paper was excluded as it is not a systematic review
Bouma et al.	2015	Bouma G, Admiraal JM, de Vries EG, Schröder CP, Walenkamp AM, Reyners AK. Internet-based support programs to alleviate psychosocial and physical	This review was excluded as it was not cancer survivors, who have completed active treatment.

For peer review only

		symptoms in cancer patients: a literature analysis. *Critical reviews in oncology/hematology.* 2015.	
Bourke et al.	2016	Bourke L, Smith D, Steed L, Hooper R, Carter A, Catto J, Albertsen PC, Tombal B, Payne HA, Rosario DJ. Exercise for men with prostate cancer: a systematic review and meta-analysis. *European urology.* 2016.	This review was excluded as it was not cancer survivors, who have completed active treatment.
Post et al.	2016	Post KE, Flanagan J. Web based survivorship interventions for women with breast cancer: An integrative review. European *Journal of Oncology Nursing*. 2016.	This paper was excluded as QOL was not main outcome.

For peer review only

Annex1: Full search strategy

Component 1: Population

#1. Neoplasms (mesh term) or cancer or cancers or cancerous or carcinoma* or neoplas* or tumor* or tumour* or malignan*

Component 2: Intervention

#2. Counseling (mesh term) or psychotherapy (mesh term) or "cognitive therapy" (mesh term) or "self-help groups" (mesh term) or "mind body therapies" (mesh term) or "behavior therapy" (mesh term) or psychotherapy, group (mesh term) or meditation, (mesh term) or "mindfulness" (mesh term) behaviour therapies, cognitive (mesh term)

#3. (counsel*:ti,ab or psychoeducat*:ti,ab or educat*:ti,ab or coping*:ti,ab or psychological*:ti,ab or psychosocial*:ti,ab or psychotherap*:ti,ab or psychoanalytic*:ti,ab) AND (therap*:ti,ab or treatment*:ti,ab or outcome*:ti,ab or intervention*:ti,ab)
#4. (social: ti,ab or peer: ti,ab or group: ti,ab) AND (support: ti,ab)
#5. self:ti,ab AND help:ti,ab
#6. (cognitive:ti,ab or behav*:ti,ab) AND (treatment*:ti,ab or therap*:ti,ab)
#7. "CBT":ti,ab
#8. (Family:ti,ab or couple:ti,ab) AND (therap*:ti,ab)
#9. meditation:ti,ab or mindfulness:ti,ab
#10. #2 or#3or#4or#5or#6or#7or#8+or#9

Component 3: Outcome

#11. "quality of life" (mesh term) or "well being": ti,ab or "QoL" (all fields) or "quality of life": ti,ab

FULL PICO:

#1 AND #10 AND #11

Filters: Humans, English language, Reviews, Age group

For peer review only

RISMA Checklist

	#	Checklist item
	1	Identify the report as a systematic review, meta-analysis, or both.
ary	2	Provide a structured summary including, as applicable: background; objectives; data sources; study eligibility criteria, participants, and interventions; study appraisal and synthesis methods; results; limitations; conclusions and implications of key findings; systematic review registration number.
	3	Describe the rationale for the review in the context of what is already known.
	4	Provide an explicit statement of questions being addressed with reference to participants, interventions, comparisons, outcomes, and study design (PICOS).
tration	5	Indicate if a review protocol exists, if and where it can be accessed (e.g., Web address), and, if available, provide registration information including registration number.
	6	Specify study characteristics (e.g., PICOS, length of follow-up) and report characteristics (e.g., years considered, language, publication status) used as criteria for eligibility, giving rationale.
es	7	Describe all information sources (e.g., databases with dates of coverage, contact with study authors to identify additional studies) in the search and date last searched.
	8	Present full electronic search strategy for at least one database, including any limits used, such that it could be repeated.
	9	State the process for selecting studies (i.e., screening, eligibility, included in systematic review, and, if applicable, included in the meta-analysis).
ocess	10	Describe method of data extraction from reports (e.g., piloted forms, independently, in duplicate) and any processes for obtaining and confirming data from investigators.
	11	List and define all variables for which data were sought (e.g., PICOS, funding sources) and any assumptions and simplifications made.
vidual studies	12	Describe methods used for assessing risk of bias of individual studies (including specification of whether this was done at the study or outcome level), and how this information is to be used in any data synthesis.

For peer review only

RISMA Checklist

es	13	State the principal summary measures (e.g., risk ratio, difference in means).
s	14	Describe the methods of handling data and combining results of studies, if done, including measures of consistency (e.g., I^2) for each meta-analysis.

Page 1 of 2

	#	Checklist item
studies	15	Specify any assessment of risk of bias that may affect the cumulative evidence (e.g., publication bias, selective reporting within studies).
s	16	Describe methods of additional analyses (e.g., sensitivity or subgroup analyses, meta-regression), if done, indicating which were pre-specified.
	17	Give numbers of studies screened, assessed for eligibility, and included in the review, with reasons for exclusions at each stage, ideally with a flow diagram.
ics	18	For each study, present characteristics for which data were extracted (e.g., study size, PICOS, follow-up period) and provide the citations.
studies	19	Present data on risk of bias of each study and, if available, any outcome level assessment (see item 12).
al studies	20	For all outcomes considered (benefits or harms), present, for each study: (a) simple summary data for each intervention group (b) effect estimates and confidence intervals, ideally with a forest plot.
s	21	Present results of each meta-analysis done, including confidence intervals and measures of consistency.

For peer review only

RISMA Checklist

studies	22	Present results of any assessment of risk of bias across studies (see Item 15).
s	23	Give results of additional analyses, if done (e.g., sensitivity or subgroup analyses, meta-regression [see Item 16]).
nce	24	Summarize the main findings including the strength of evidence for each main outcome; consider their relevance to key groups (e.g., healthcare providers, users, and policy makers).
	25	Discuss limitations at study and outcome level (e.g., risk of bias), and at review-level (e.g., incomplete retrieval of identified research, reporting bias).
	26	Provide a general interpretation of the results in the context of other evidence, and implications for future research.
	27	Describe sources of funding for the systematic review and other support (e.g., supply of data); role of funders for the systematic review.

Liberati A, Tetzlaff J, Altman DG, The PRISMA Group (2009). Preferred Reporting Items for Systematic Reviews and Meta-Analyses: The PRISMA State
doi:10.1371/journal.pmed1000097

For more information, visit: www.prisma-statement.org.

For peer review only

For peer review only - http://bmjopen.bmj.com/site/about/guidelines.xhtml
